# Torpor enhances synaptic strength and restores memory performance in a mouse model of Alzheimer’s disease

**DOI:** 10.1038/s41598-021-94992-x

**Published:** 2021-07-29

**Authors:** Christina F. de Veij Mestdagh, Jaap A. Timmerman, Frank Koopmans, Iryna Paliukhovich, Suzanne S. M. Miedema, Maaike Goris, Rolinka J. van der Loo, Guido Krenning, Ka Wan Li, Huibert D. Mansvelder, August B. Smit, Robert H. Henning, Ronald E. van Kesteren

**Affiliations:** 1 Department of Molecular and Cellular Neurobiology, Center for Neurogenomics and Cognitive Research, VU University, Amsterdam, The Netherlands; 2 Department of Clinical Pharmacy and Pharmacology, University Medical Center Groningen, Groningen, The Netherlands; 3 Department of Integrative Neurophysiology, Center for Neurogenomics and Cognitive Research, VU University, Amsterdam, The Netherlands; 4 Department of Pathology and Medical Biology, University Medical Center Groningen, Groningen, The Netherlands; 5 Sulfateq B.V., Groningen, The Netherlands

**Keywords:** Hippocampus, Animal physiology

## Abstract

Hibernation induces neurodegeneration-like changes in the brain, which are completely reversed upon arousal. Hibernation-induced plasticity may therefore be of great relevance for the treatment of neurodegenerative diseases, but remains largely unexplored. Here we show that a single torpor and arousal sequence in mice does not induce dendrite retraction and synapse loss as observed in seasonal hibernators. Instead, it increases hippocampal long-term potentiation and contextual fear memory. This is accompanied by increased levels of key postsynaptic proteins and mitochondrial complex I and IV proteins, indicating mitochondrial reactivation and enhanced synaptic plasticity upon arousal. Interestingly, a single torpor and arousal sequence was also sufficient to restore contextual fear memory in an APP/PS1 mouse model of Alzheimer’s disease. Our study demonstrates that torpor in mice evokes an exceptional state of hippocampal plasticity and that naturally occurring plasticity mechanisms during torpor provide an opportunity to identify unique druggable targets for the treatment of cognitive impairment.

## Introduction

Hibernation is a state of inactivity during which animals undergo periods of extreme hypometabolism and hypothermia to escape energetically challenging environmental conditions^1^. During hibernation, bouts of hypometabolism (i.e., torpor) typically last several days to weeks, and are alternated with short periods of rapid restoration of metabolism to normal values (i.e., interbout arousals). Smaller species may use a different pattern of daily hibernation, during which they deploy 6–12 h of torpor on a daily basis, and effectively experience a full hibernation cycle of torpor and arousal within several hours^2,3^.

Previous studies in seasonal hibernators such as Syrian hamster and ground squirrel have demonstrated an exceptionally high degree of structural plasticity in the brain during hibernation, including extensive hippocampal dendritic retraction and changes in spine morphology and spine numbers^4^. These changes are paralleled by widespread hyper-phosphorylation of the microtubule-associated protein Tau, reaching levels that are pathological in humans and trigger formation of intracellular Tau aggregates as observed in Alzheimer’s disease (AD). Remarkably, arousal fully restores dendritic and synaptic integrity and reverses Tau hyper-phosphorylation and aggregation without post-hibernation damage^5–7^. This features hibernation-based plasticity as an interesting mechanism from which novel treatments can be derived for neurodegenerative diseases in which neuronal plasticity is impaired.

To date, mechanisms underlying torpor-associated plasticity in the brain are still poorly understood. Previous studies in seasonal hibernators failed to unambiguously identify effects of torpor on normal brain function. For instance, following hibernation, retention of pre-torpor memory has been reported to be either disrupted^8,9^, enhanced^10,11^, or unaffected^5,12^, possibly depending on the species and memory paradigm used. Given the rapid restoration of brain morphology and the extent of biochemical changes, arousal may be viewed as a period of exceptional neuroplasticity, and further insight into its underlying mechanisms may both advance our understanding of adult brain plasticity and aid the identification of treatment targets for neurodegenerative diseases^13,14^. The notion that laboratory mice are capable of daily torpor^15–18^ not only offers the opportunity to explore arousal-associated plasticity mechanisms in wildtype mice, but also allows to test torpor-derived interventions in mouse models of disease.

Here, we used fasting-induced torpor in mice to study arousal-associated structural, functional and molecular adaptations in the hippocampus and its effects on memory acquisition. We show that torpor in mice mainly acts on synaptic plasticity and mitochondria, and is associated with increased long-term potentiation (LTP) and memory performance after arousal in wildtype and in an APP/PS1 mouse model of AD. Thus, plasticity mechanisms during torpor may provide a unique opportunity to identify novel targets for the treatment of neurodegenerative diseases.

## Results

### Fasting-induced torpor in mice

**Figure 1:**
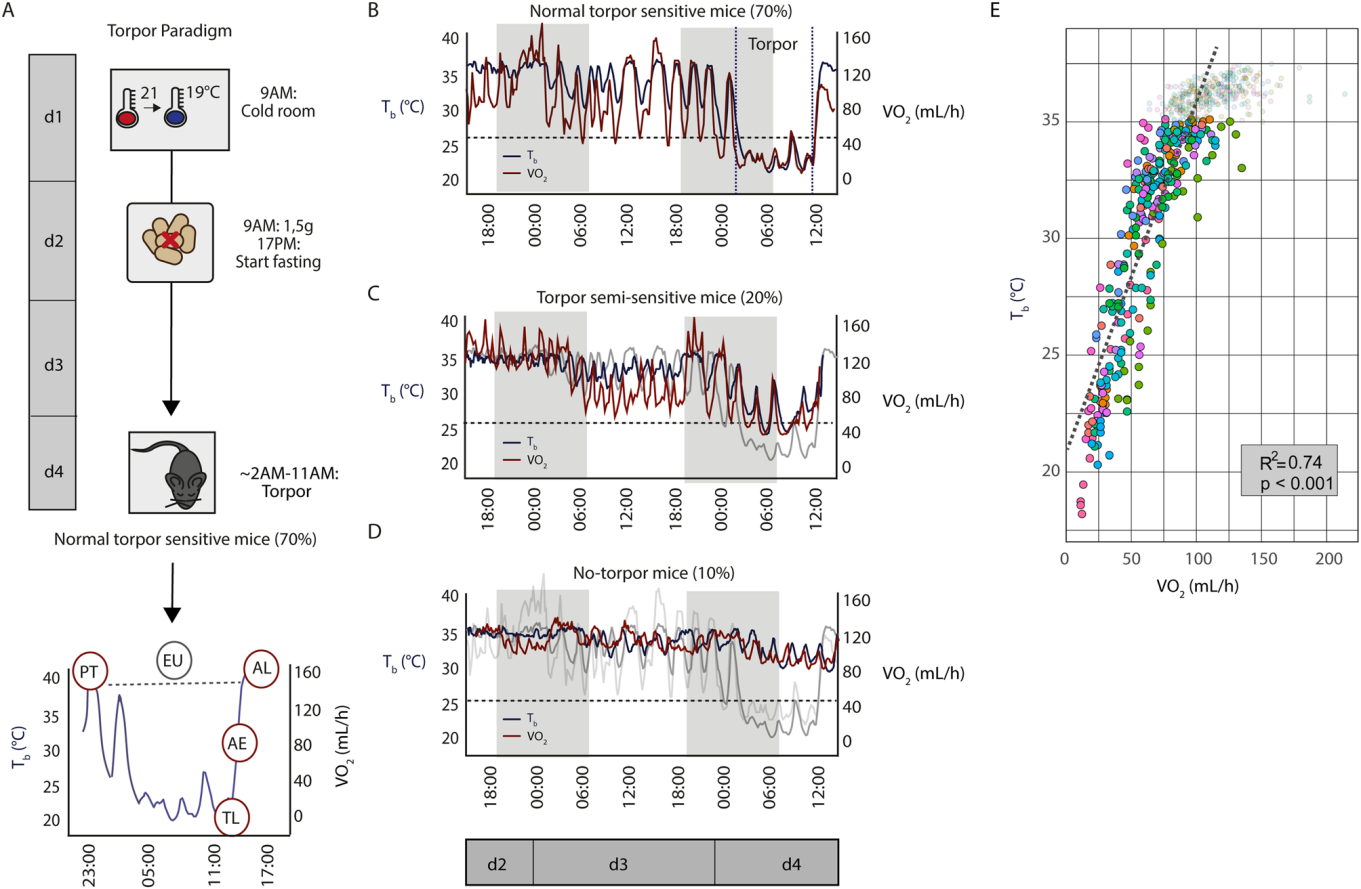
Torpor induction in mice. (**A**) Steady torpor is induced in mice using ambient temperature reduction on day 1 (T_a_ 21 → 19 °C) and food restriction on day 2 (1.5 g from 9:00AM till 17:00PM), followed by a fasting period of maximally 40 h. During the second night of fasting, ~ 70% of the mice enter torpor. Torpor stages were defined as pre-torpor (PT; T_b_ > 36 °C / VO_2_ > 120 mL/h); torpor late (TL; T_b_ < 26 °C and/or VO_2_ < 40 mL/h for at least 6 h); arousal early (AE; T_b_ of ~ 30 °C and VO_2_ of ~ 80 mL/h); arousal late (AL; T_b_ 37 °C and VO_2_ > 120 mL/h for 2 h). Euthermic (EU) animals were held at normal housing temperature and fed ad lib. (**B**) Representative core body temperature (T_b_) and metabolic rate (VO_2_) graphs of normal torpor sensitive mice (70%; *n* = 21/30 mice). An average torpor bout lasted 9 ± 0.65 h (*n* = 12) with T_b_ (blue line) dropping to ~ 21 °C and VO_2_ (red line) dropping to ~ 20 mL/h VO_2_. (**C**) Torpor semi-sensitive mice (20%; *n* = 6/30 mice) did not reach stable T_b_ < 26 °C, and (**D**) no-torpor mice (10%; *n* = 3/30 mice) do not enter torpor after two nights of fasting. Normal torpor graphs are shown in grey for reference. (**E**) T_b_ (°C) and VO_2_ (mL/h) were highly correlated (*n* = 12 mice; R^2^ = 0.74, *p* < 0.001).

We first established a method to induce stable torpor in mice. Torpor was induced by a reduction of ambient temperature (T_a_) from 21 to 19 °C for 96 h with a limitation of food availability to 1.5 g during the first inactive phase, followed by a maximum of 40 h of fasting (two times overnight till 12:00AM on day 4). Torpor was successfully induced when an animal’s core body temperature (T_b_) reached < 26 °C for at least 6 h. Different torpor phases were defined (Fig. 1A): pre-torpor (PT) is the phase just before hypothermia is instigated; torpor late (TL) is at the end of the hypothermic phase (T_b_ < 26 °C for at least 6 h); arousal early (AE) is half way through the arousal phase when T_b_ reaches ~ 30 °C; and arousal late (AL) is when mice are fully aroused and have reached a T_b_ > 36 °C for ~ 2 h. Euthermic (EU) control mice were maintained on food ad libitum at a T_a_ of ~ 21 °C and did not enter torpor. Metabolic measurements confirmed that lowered body temperature coincides with a lowered oxygen consumption (VO_2;_ mL/h) (Fig. 1B–D). The torpor paradigm induced steady and profound torpor in 70% of mice (normal torpor-sensitive mice; Fig. 1B), while 20% of the mice did not reach temperatures below 26 °C for more than 6 h, yet showed intermittent T_b_ drops and metabolic rate reduction (torpor semi-sensitive mice; Fig. 1C), and 10% did not enter torpor at all after two nights of fasting (no-torpor mice, used as metabolic control mice in this study; Fig. 1D). Since VO_2_ and T_b_ were always strongly correlated (Fig. 1E), only T_b_ was used to monitor animals in all subsequent experiments.

### Dendrite morphology and spine numbers are preserved during torpor in mice

**Figure 2:**
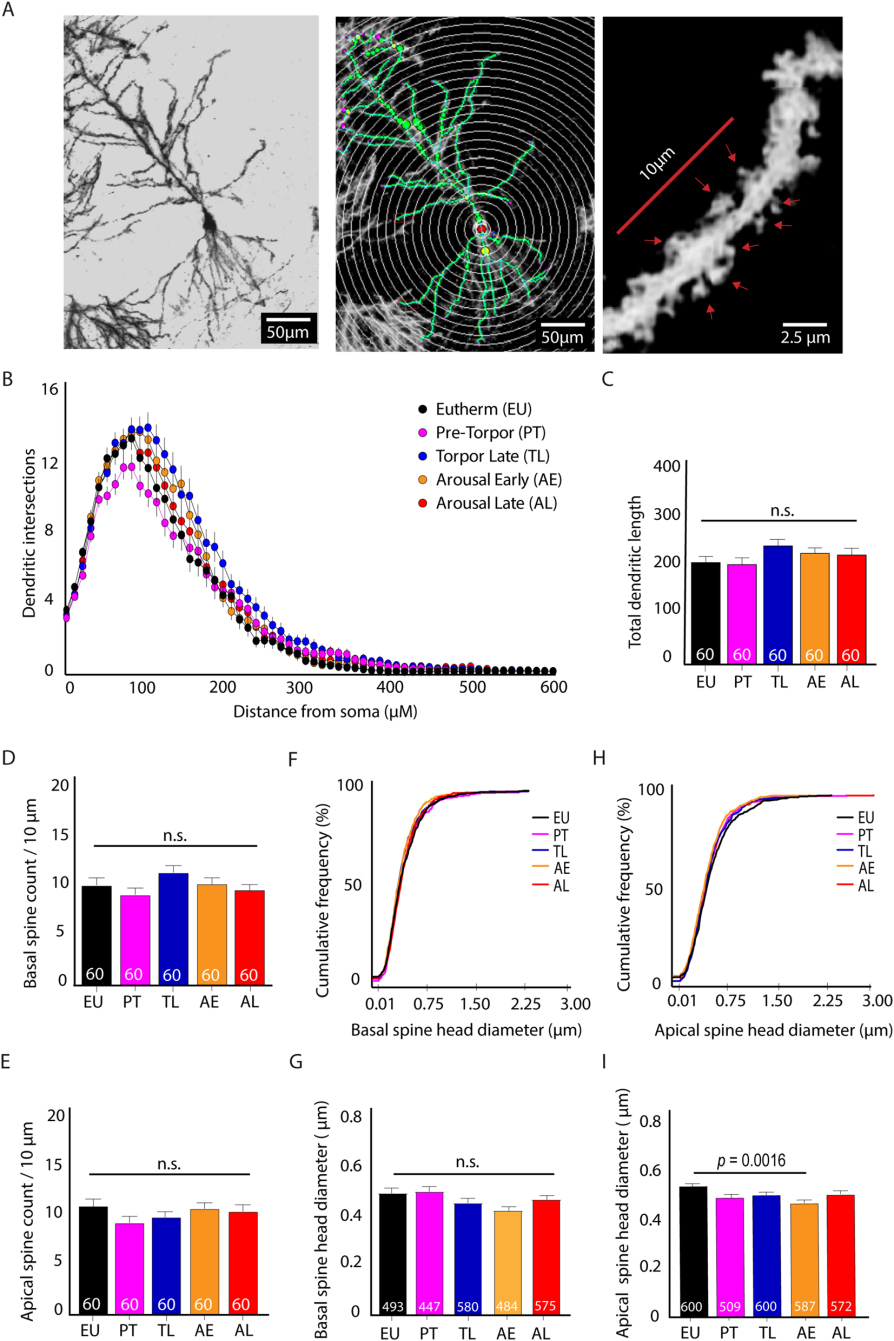
Torpor does not affect neuronal structural integrity in mice. (**A**) Representative images of a Golgi-Cox stained CA1 pyramidal neuron at 10 × magnification (left), a tracing (green) and Scholl analysis (white) of the same neuron (middle; 1 soma radius = 10 µm) and a 40 × magnification used for spine counting (red arrows). (**B**) Scholl analysis revealed no significant differences in dendritic complexity between torpor phases (EU, PT, TL, AE and AL; *n* = 10 neurons per animal from 6 animals per group; Two-way ANOVA F_4,296_ = 2.022, *p* = 0.0913). (**C**) Total dendritic length of CA1 pyramidal neurons did also not differ significantly between groups (One-way ANOVA F_4,295_ = 2.244, *p* = 0.63). (**D-E**) Basal (**D**) and apical (**E**) spine count did not differ significantly between groups either (One-way ANOVA F_4,295_ = 2.044, *p* = 0.22 and F_4,295_ = 1.077, *p* = 0.37, respectively). (**F-G**) Cumulative frequency distributions (**F**) and geometric means (**G**) of basal spine head diameters did not reveal significant differences among groups (Kruskall-Wallis *p* = 0.90; One-way ANOVA F_4,2574_ = 2.316, *p* = 0.055). (**H-I**) Cumulative frequency distributions (**H**) and geometric means (**I**) of apical spine head diameter revealed a significant decrease in spine head diameter at AE compared to EU in geometric means only (0.44 ± 0.01 µm vs 0.38 ± 0.010 µm; One-way ANOVA F_4,2863_ = 5.346, *p* = 0.0015; post-hoc Tuckey *p* = 0.0016), not in frequency distribution (Kruskall-Wallis *p* = 0.92). Spine head diameter was restored again in AL.

CA1 hippocampal pyramidal neuron structure during torpor were assessed using Golgi-Cox staining. In particular, we analyzed dendrite complexity (dendritic branching) and size (total dendritic length) as well as spine numbers and spine head diameter (Fig. 1A), as these are strongly affected in seasonal hibernators^4,5,19–21^. Scholl analysis of CA1 pyramidal neurons (Fig. 2A) showed no differences in dendritic branching between euthermic (EU) controls and mice at any hibernation stage (PT, TL, AE and AL) (Fig. 2B). Furthermore, neither total dendrite length, nor the number of basal or apical spines differed between groups (Fig. 2C–E). These data demonstrate that major structural changes such as dendritic retraction or spine loss, as observed in seasonal hibernators, are absent during or after a single torpor bout in mice. When basal and apical spine morphology were assessed, a minor reduction in spine head diameter was observed during torpor (Fig. 2F–I) which only reached significance for apical spines at AE compared to EU animals (Fig. 2I). Spine head diameters were restored again in AL animals.

### Long-term potentiation and contextual fear memory are transiently enhanced after torpor

**Figure 3:**
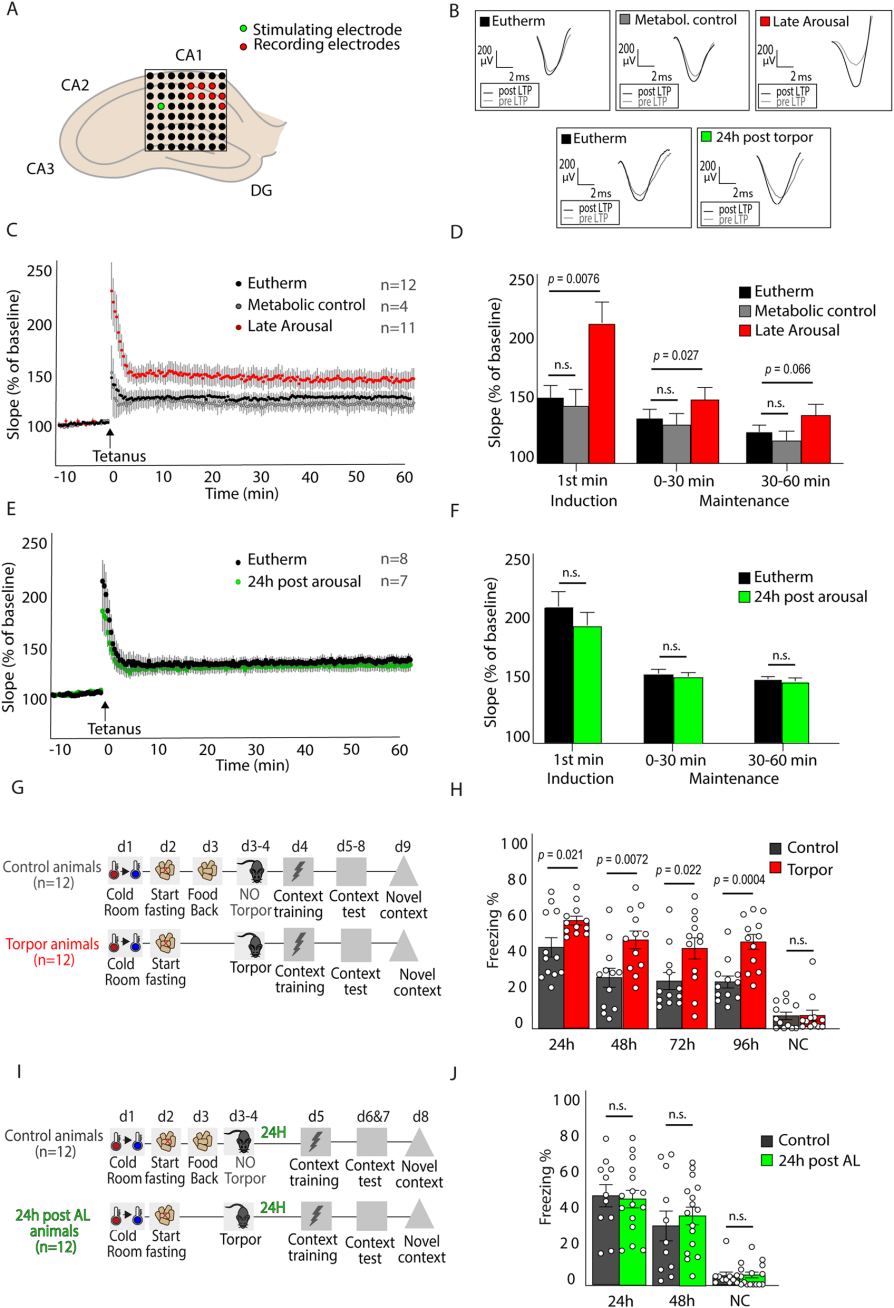
Hippocampal LTP and memory are enhanced during arousal. (**A**) LTP was measured using a 64-electrode grid. One electrode was used to stimulate the Schaffer collateral pathway and 6–8 electrodes were used to record field potentials in CA1. (**B**) Representative pre- and post-tetanus fEPSP traces of EU (*n* = 12), metabolic control (*n* = 4) and AL (*n* = 11) animals and of EU (*n* = 8) and 24 h post-arousal (*n* = 7) animals. (**C**) LTP was measured as the fEPSP slope as % of baseline. (**D**) A significant increase in fEPSP slope was observed in AL versus EU in the first minute after tetanus (AL: 219.1% ± 22.1%, EU: 153.4% ± 9.5%; One-way ANOVA F_2,25_ = 5.239, *p* = 0.0126) and for the first 30 min after tetanus (AL: 153.0% ± 9.6%, EU: 128.8% ± 5.7%; One-way ANOVA F_2,25_ = 3.513, *p* = 0.0452; post-hoc Fisher’s LSD *p* = 0.0076, *p* = 0.027 and *p* = 0.066 for 0–1 min, 0–30 min and 30–60 min, respectively). LTP in EU mice and metabolic control mice did not differ significantly. (**E**) LTP induction was separately determined for EU and 24 h post-arousal mice. (**F**) No significant differences in LTP were observed between EU and 24 h post-arousal mice (Student’s *t*-test;* p* > 0.05). (**G**) To test contextual fear memory, torpor mice and no-torpor control mice received a 0.7 mA footschock on day 4 (d4; the late arousal phase of the torpid mice) and were tested in the same context 24 h, 48 h, 72 h and 96 h later, and in a novel context (NC) on d9. (**H**) Freezing levels were significantly increased on all 4 time points for torpor mice compared with no-torpor control mice (24 h: control 44.6% ± 5.4% versus torpor 59.1% ± 2.4%; 48 h: 27.8% ± 5.2% versus 48.0% ± 5.8%; 72 h: 26.0% ± 4.7% versus 43.0% ± 5.8%; 96 h: 25.7% ± 3.3% versus 46.6% ± 4.1%; Student’s *t*-test *p* = 0.021, *p* = 0.0072, *p* = 0.022 and *p* = 0.0004 for 24 h, 48 h, 72 h and 96 h, respectively), indicating stronger fear memory in torpor mice lasting at least 4 days. Little freezing was measured in the NC (control 6.8% ± 2.1%; arousal 6.2% ± 3.0%). (**I,J**) Fear conditioning at 24 h after arousal showed no differences in freezing levels between 24 h post-AL mice and control mice at 24 h or 48 h after acquisition (Student’s *t*-test; *p* = 0.8518 and 0.5326 respectively).

The observed changes in hippocampal spine morphology during torpor and arousal, in particular the normalization of apical spine head diameter in AL, prompted us to test whether hippocampal synaptic transmission is altered during arousal. Field-stimulated long-term potentiation (LTP) was measured in the hippocampal CA1 region of AL, EU, metabolic control and 24 h post-torpor mice (Fig. 3A,B). Post-tetanic potentiation at 1 min after tetanus stimulation and up to 30 min after tetanus stimulation were significantly increased in AL compared to EU, while LTP in EU mice did not differ significantly from metabolic control mice (Fig. 3C,D). At 24 h post-torpor, no increase in LTP was observed anymore (Fig. 3E,F). These data show that late arousal features a transient state of increased synaptic plasticity in the hippocampus.

We next tested whether memory formation is also increased at the same time point at which we observed enhanced LTP. We chose a contextual fear memory paradigm which induces instant learning at AL or 24 h later in mice that underwent torpor (Fig. 3G–J). Control mice were food-restricted up to the evening before the torpor bout and then fed at the start of the dark phase (7:00 PM) to prevent torpor. After memory acquisition, freezing levels were measured every 24 h in both groups in the acquisition context up to 96 h (AL group) or 48 h (24 h post-torpor group) after acquisition (Fig. 3G,I). Contextual fear memory of AL mice showed a significant increase, which lasted for at least 4 days (Fig. 3H). In contrast, mice conditioned at 24 h post-torpor did not show an increase in freezing levels when tested 24 h and 48 h after conditioning (Fig. 3J). None of the groups showed substantial freezing in a novel context (Fig. 3H,J). Arousal and control animals did not show differences in activity upon context acquisition during fear conditioning (Fig. S1). These findings show that an increase in hippocampal LTP at AL is paralleled by a transient increase in hippocampus-dependent memory.

### Regulation of synaptic protein levels during torpor and arousal

**Figure 4:**
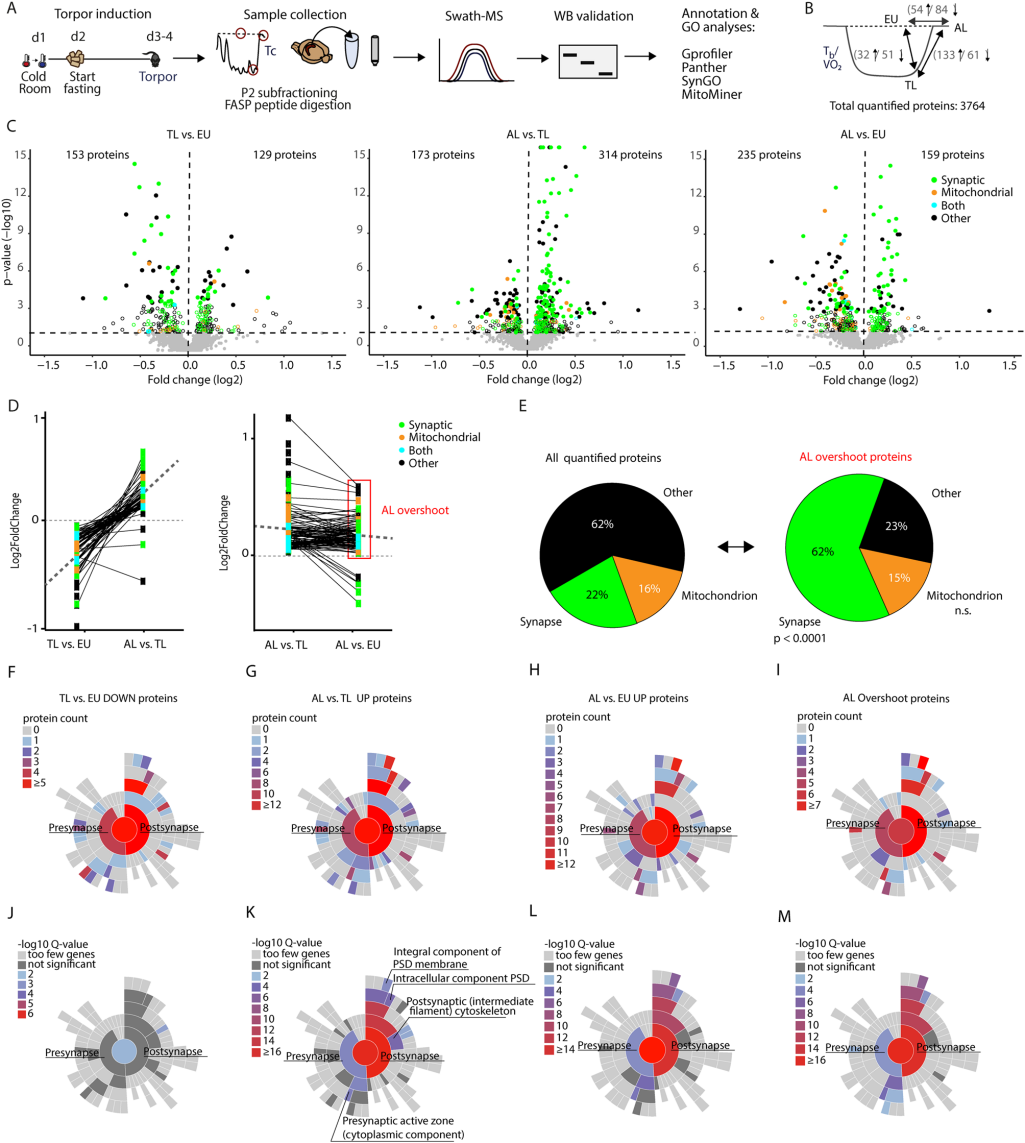
Changes in synaptic and mitochondrial protein levels during torpor and arousal. (**A**) After torpor induction, hippocampal protein samples were prepared from EU, TL and AL mice (*n* = 6 per group). P2 fractions were used for quantitative mass spectrometry. Significantly regulated proteins were validated with Western blotting, and ontology enrichment was used for functional interpretation. (**B**) In total, 3764 proteins were quantified. Focusing on three contrasts, TL versus EU, AL versus EU and AL versus TL, 138 (54 up and 84 down), 83 (32 up and 51 down) and 194 (133 up and 61 down) significantly regulated proteins were identified after multiple testing correction (FDR; *q* < 0.05). (**C**) Volcano plots showing the -10log *p*-value (y-axis) and the log2 fold change (x-axis) of all quantified proteins in the three different contrasts. Dashed lines indicate the *p* < 0.05 cutoff (Student’s *t*-test), FDR corrected significant proteins with *q* < 0.05 are depicted as filled dots. Colors indicate proteins that were annotated to the synapse (green), mitochondrion (orange), both (blue) or other cellular components (black). (**D**) Downregulated proteins in TL versus EU were typically upregulated in AL versus TL (left), and upregulated proteins in AL versus TL were typically also upregulated in AL versus EU (right). Dashed lines depict mean regulation. The red box marks ‘AL overshoot’ proteins (*n* = 99) that are upregulated from TL to AL, and in AL show significantly higher expression than in EU controls. (**E**) Pie charts showing a significant overrepresentation of the cellular component term *synapse* (green) in ‘AL overshoot’ proteins compared with all quantified proteins (Fisher’s Exact test; *p* < 0.001). *Mitochondrion* (orange) is the second most abundant term in this group, although not significantly enriched. (**F-M**) Functional annotation and enrichment of synaptic proteins were further determined with SynGO. Postsynaptic proteins in particular show relatively high counts in downregulated proteins in TL versus EU (**F**), in upregulated proteins in AL versus TL (**G**) and AL versus EU (**H**), and in ‘AL overshoot’ proteins (**I**). (**J-M**) Significant enrichment of synaptic SynGO terms was observed primarily in upregulated protein groups (AL vs TL, AL vs EU and ‘AL overshoot’; *q* < 0.05), and were predominantly postsynaptic in nature and associated with the postsynaptic density (PSD).

Protein levels in hippocampal P2 fractions enriched for synaptic proteins were quantified by differential expression analysis (DEA; see methods) in AL, TL and EU (Fig. 4A). The total number of detected proteins was 3764. Differentially regulated proteins were determined by pairwise comparisons between TL, AL and EU. After multiple testing correction we found 83 (32 up-; 51 downregulated) significantly regulated proteins in TL versus EU, 194 (133 up-; 61 downregulated) in AL versus TL and 138 (54 up-; 84 downregulated) in AL versus EU (FDR, *q* < 0.05) (Fig. 4B; Supplementary Dataset 1). Immunoblotting for 4 significantly regulated proteins confirmed the direction of regulation (Fig. S2).

For a broader comparison of differential protein expression in all three contrasts we plotted all regulated proteins with an uncorrected *p* value of < 0.05 (Student’s t-test) in volcano plots and annotated proteins according to cellular component GO terms (Fig. 4C). Two cellular components were visually overrepresented: the synapse and the mitochondrion. In general, significantly regulated proteins were mostly downregulated TL versus EU and mostly upregulated in AL versus TL. Moreover, of all proteins that were significantly downregulated from in TL versus EU, almost all (64/67) were upregulated again in AL versus TL (Fig. 4D, left panel), indicating that torpor and arousal are marked by a similar but opposite protein expression signatures. Interestingly, of the proteins that were upregulated in AL versus TL and that were also differentially expressed between AL and EU, almost all (99/105) were upregulated in AL versus EU (Fig. 4D, right panel), indicating an ‘overshoot’ in protein expression during AL above baseline (EU). These 99 ‘AL overshoot’ proteins are of particular interest to understand mechanisms of torpor-induced plasticity during AL, and were therefore separately tested these for enrichment of synaptic and mitochondrial proteins compared to all quantified proteins (Fig. 4E, Supplementary dataset 2). Cellular component annotation by Gprofiler^22^, Mitominer^23^ and SynGO^24^ showed that 65/105 proteins (62%) are annotated to the synapse, which is significantly more than the 22% synaptic proteins in all quantified proteins (Fisher’s exact test, *p* < 0.001). The second largest portion of regulated proteins is annotated to the mitochondrion, although not significantly different than in the total set of measured proteins (16% and 15%, respectively). These findings confirm what was also visually observed in the volcano plots (Fig. 4C).

### Arousal causes an overshoot in expression of plasticity promoting postsynaptic proteins

We next performed in-depth synaptic functional annotation using SynGO, a dedicated and curated synapse ontology database^24^. Cellular component (CC) annotation of downregulated proteins in TL versus EU (Table S1), upregulated proteins in AL versus TL (Table S2), upregulated proteins in AL versus EU (Table S3), and AL overshoot proteins (Table S4) revealed that most proteins are postsynaptic in all four groups (Fig. 4F–I; protein count). Moreover, upregulated proteins in AL versus TL and in AL versus EU, as well as AL overshoot proteins, were significantly enriched for postsynaptic ontology terms (Fig. 4J–M). Enrichment for presynaptic proteins was substantially less and included in particular some protein components of the presynaptic active zone. Likewise, annotation to biological processes (BP) showed enrichment for postsynaptic processes (Fig. S3, Tables S5-8).

Enriched SynGO terms predominantly contained AMPA and NMDA receptor subunits (GLUA1, GLUA2, GLUN1, GLUN2A and GLUN2B), AMPA/NMDA receptor auxiliary subunits (e.g. SHISA6, SHISA7 and CACNG8), postsynaptic density proteins (e.g. CAMK2A, CAMK2B, DLG2, DLG3, DLG4, DLGAP1, DLGAP2, DLGAP3, HOMER1, HOMER2, HOMER3, SHANK1, SHANK2, SHANK3, SYNGAP1) and neurofilament proteins (INA, NEFH, NEFL, NEFM). These proteins are well known organizers of postsynaptic signal transduction and plasticity, and are likely involved in the increased synaptic potentiation observed during arousal.

In contrast, downregulated proteins in TL versus EU showed overlap with proteins upregulated in AL but lacked significant enrichment for synaptic CC or BP SynGO terms, suggesting that other processes may be involved in entering torpor, diluting the effect of postsynaptic protein regulation. Upregulated proteins for TL versus EU, and downregulated proteins for AL versus TL and AL versus EU showed no apparent enrichment (data not shown). Taken together, a marked increase in postsynaptic plasticity protein expression is associated with arousal after torpor.

### Torpor rescues memory deficits in APP/PS1 mice

**Figure 5:**
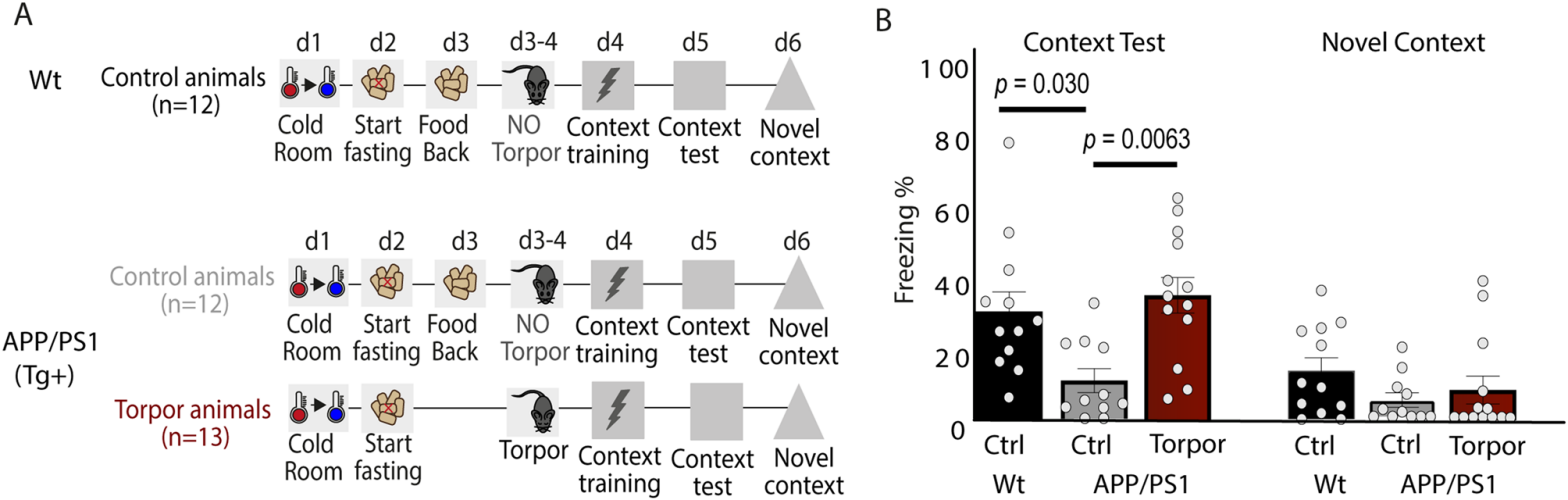
Torpor rescues memory deficits in APP/PS1 mice. (**A**) Torpor was induced in APP/PS1 mice. As controls, wildtype (Wt) and APP/PS1 mice were used that were refed to prevent torpor entry. All mice underwent fear conditioning on day 4 (d4) and context exposore on d5. On d6, they were tested in a novel context (NC). (**B**) Freezing levels were significantly diferent between groups (One-way ANOVA F_2,34_ = 4.646, *p* = 0.0164). Freezing levels were significantly lower in APP/PS1 control mice compared with wildtype control mice (14.3% ± 4.7% vs 30.7% ± 5.5%; post-hoc Fisher’s LSD; *p* = 0.030), confirming a memory impairment in APP/PS1 mice. Torpor rescued freezing in APP/PS1 mice up to wildtype levels (35.0% ± 4.7% vs 30.7% ± 5.5%; post-hoc Fisher’s LSD; *p* = 0.0063). Little freezing was observed in the NC (wildtype control: 21.3% ± 4.6%, APP/PS1 control 4.7% ± 2.0%, APP/PS1 torpor 8.5% ± 3.9%).

Given the effects of post-torpor arousal on synaptic plasticity and memory, we next tested whether torpor also rescues memory deficits in an APP/PS1 mouse model of AD, which shows decreased synaptic plasticity and a memory impairment at 6 months of age^25,26^. Control mice (APP/PS1 and wildtype) were food-restricted up to the evening prior to the expected torpor bout, but were prevented from entering torpor by re-feeding. Mice were exposed to contextual fear condition during AL and freezing levels were measured in the acquisition context 24 h after memory acquisition (Fig. 5A). Control APP/PS1 mice had significantly impaired contextual fear memory compared to wildtype controls, whereas APP/PS1 mice that underwent torpor showed a significant increase in freezing up to the level of wildtype control mice after memory acquisition in AL (Fig. 5B), indicating a rescue of contextual fear memory post-torpor. No substantial freezing was measured in a novel context.

## Discussion

We established a robust fasting paradigm that induces profound and steady torpor in the large majority of standard laboratory C57BL/6J mice. We show that, while torpor does not invoke major structural neuronal changes in hippocampus, it does affect synapses molecularly and functionally. In particular, arousal in mice is accompanied by increasing levels of proteins involved in postsynaptic organization and plasticity, which is paralleled by an enhancement of LTP and of contextual memory. Finally, torpor induction rescues memory in an APP/PS1 mouse model of AD. Together, these data identify potential mechanisms by which torpor induction enhances hippocampal synaptic transmission and memory performance, and may provide novel entry points for the treatment of reduced plasticity in neurodegenerative diseases.

Studying torpor requires robust experimental models and stable laboratory settings. Here, we used a stringent definition of torpor (T_b_ < 26 °C for > 6 h continuously), whereas most previous mouse studies defined torpor as temperatures below ~ 31 °C^11,15,27^. We excluded mice that displayed discontinuous bouts < 6 h at T_b_ < 26 °C, which allowed us to accurately identify ‘switch’ moments of entering and leaving torpor and exactly time the durations of arousal periods. Moreover, it also allowed us to reproducibly measure the impact of torpor on structural, physiological, molecular and behavioral plasticity.

An important finding was that our torpid mice did not show any signs of structural reorganization in the hippocampus other than a small but significant decrease in apical spine head diameter for AE mice versus EU mice, possibly indicating dematuration of synapses^28^. Spine head diameter was fully restored at AL, suggesting increased spine plasticity during arousal. These findings are in sharp contrast with the dendrite retraction and reduction in spine numbers reported in seasonal hibernators^4,20,21^. This may be related to the difference in core body temperature during torpor, with mice only reaching down to 20–21 °C, while seasonal hibernators typically reach 4 °C or lower. Another explanation may be the shorter duration of torpor in mice, lasting up to 10 h, whereas torpor in seasonal hibernators typically lasts at least several days and can last up to weeks^3^.

In spite of the absence of overt structural reorganization, torpor induction in mice provoked compelling molecular and functional adaptations at the synaptic level. We observed a substantial and significant increase in post-tetanic potentiation upon torpor induction during AL. In keeping with enhanced synaptic potentiation being the basis for associative learning and memory^29^, AL mice also showed enhanced performance in a hippocampus-dependent contextual fear memory test. This post-torpor increase in plasticity was transient and lasted less than one day, as LTP and fear memory did not differ from EU control animals when induced 24 h after arousal. Importantly, the absence of effects on LTP in fasted animals that did not enter torpor (no torpor metabolic controls) and on fear memory acquisition in animals conditioned for torpor but refed, implies that fasting and lower ambient temperature alone are not sufficient to increase plasticity. In that respect, it is important to mention that hibernation or torpor are not the same as sleep. If anything, hibernation induces a state of sleep deprivation, as normal brain function during hypothermia is halted and NREM activity is increased after torpor, similar to sleep deprivation^30,31^. Moreover, sleep deprivation is known to impair memory^32^. It thus seems very unlikely that sleep deprivation plays a role in the observed effects of torpor on LTP and memory.

It is of interest to speculate about the plasticity state of animals that are either in torpor (TL) or in early arousal (AE). Although memory tests are not possible at these stages, LTP measurements could provide an answer to this question. Assuming that energy preservation is the main functions of torpor, one might expect that LTP is reduced or even absent in TL and is reinstated during AE. Whether it then reaches a maximum already in AE or during AL remains an open question. Our spine measurements show that apical spine head diameter remains low until AL, suggesting the latter, but additional LTP measurements are required to confirm this.

In addition to a post-torpor increase in LTP and memory, we also identified many synaptic proteins that are downregulated during torpor and become subsequently upregulated again during arousal. The particular postsynaptic signature of these proteins, together with the elevated induction and maintenance of LTP during AL and the decrease and subsequent increase in spine size, suggest a local regulation of postsynaptic plasticity during AL. Indeed, we observed an upregulation during AL of AMPA and NMDA receptor subunits, AMPA and NMDA receptor auxiliary proteins and many PSD proteins. Also, several neurofilament proteins were regulated. All these proteins have been previously linked to the modulation of synaptic plasticity^33–36^, i.e. the ability of a synapse to strengthen or weaken over time in response to a stimulus^37^, which is considered the cellular basis of increased synaptic potentiation and memory performance. Our proteomic screening approach thus uniquely reveals a protein signature of increased synaptic plasticity following torpor.

At this stage the physiological benefit of this adjustment of synapse plasticity is not clear. One possibility is that the downregulation of AMPA/NMDA receptor subunits and auxiliary subunits during torpor protects the brain against excitotoxicity^38^ and limits energy consumption by reducing synaptic communication^20^, and that the temporary post-arousal boost in plasticity and memory are simply the result of an increased expression of these proteins required to prepare the brain for its normal function again. Alternatively, increased memory formation during arousal may in itself be important to maximize the animal’s adaptive response to a new post-torpor environment, offering an evolutionary advantage of crucial importance in animals that use torpor as an emergency strategy.

Interestingly, we found that, in addition to the prominent synaptic nature of torpor-regulated proteins, a substantial fraction of upregulated proteins is mitochondrial. Notably, many of these mitochondrial proteins were assigned specifically to electron transport chain complexes I and IV, previously shown to be involved in mitochondrial activity regulation during hibernation^40–42^. The significance of torpor-induced mitochondrial activation in neuronal protection and plasticity was demonstrated in a recent study showing that preservation of mitochondrial function confers the cell autonomous protection against cold-induced stress in arctic squirrel iPSC-derived neurons^39^. Using compound BAM15, which maintains proper mitochondrial respiration during cold-induced stress, human IPSC-derived neurons were protected from morphological changes and cell death, indicating that mitochondrial intervention can be a valuable strategy for neuroprotective purposes in humans. Our data offer he possibility to find novel links between arousal-induced mitochondrial activation and enhanced synaptic plasticity and identify novel targets for torpor-derived neuroprotective and plasticity-promoting treatment.

Brain hibernation mechanisms have previously been coined to reverse the molecular pathology in AD, specifically Tau hyper-phosphorylation^5,6^. In addition to this, we now demonstrate that memory deficits are also rescued by torpor in APP/PS1 mice, a well-established amyloid-beta generating AD model. Mitochondrial and synaptic dysfunction are hallmarks of AD^40–42^. The increased expression of both mitochondrial and synaptic proteins during arousal might thus have multiple beneficial effects in AD that together improve plasticity and memory. The fact that arousal acts on multiple systems in the cell, and does so in a natural manner, makes torpor an interesting phenomenon for the identification of disease-modifying entry points for AD and possibly other neurodegenerative diseases.

Recently it was shown that a hypometabolic state in mice is conferred by a distinct subset of glutamatergic neurons in the hypothalamus^18,43^. Notwithstanding the importance of these findings, it is debatable whether humans have a similar switch, whether turning on this switch would have similar beneficial effects as in hibernators, and if so, whether instigating a state of torpor in humans would be expedient. Our study therefore importantly adds to these findings by highlighting hippocampal adaptations that are downstream of torpor induction and directly impact on synaptic plasticity and memory. Thus, torpor-associated brain mechanisms, and not torpor itself, may hold keys for the treatment of brain diseases in which plasticity is impaired.

## Materials and methods

### Animals

All experiments with animals were approved by the Animal Welfare Body (IVD) of the VU University Amsterdam in compliance with the Dutch Central Committee for Animal Experiments (CCD #16427) ordination and reported in accordance with ARRIVE guidelines^44^. C57BL/6J (Charles River) and APP/PS1 (strain B6C3-Tg(APPswe,PSEN1dE9)85Dbo/J) mice were bread locally in the Amsterdam Animal Research Center (AARC) or were ordered from Charles River. Male animals were used in all experiments. All C57BL/6J mice were of 2 months + /− 1 week of age and APP/PS1 mice were 6 months + /− 2 weeks of age at the start of the experiment. Mice were single-housed on sawdust in standard Makrolon type 2 cages (~ 21 °C ambient temperature (T_a_) and ~ 50% humidity), enriched with cardboard nesting material and chewing wood and with food and water ad lib. Mice were kept on a 12:12 light–dark cycle, with lights on at 7:00 AM.

### Temperature logger implantation

Animals were injected s.c. with 0.05 mg/kg buprenorfine 30 min prior to surgery. Real-time readable temperature loggers (Anipill; Animals Monitoring, Hérouville, France) were implanted intra-peritoneally under full anesthesia (1.5–3% isoflurane in oxygen). Post-operation analgesia (buprenorfine 0.05 mg/kg) was provided. Animals were allowed to recover from surgery for at least 1 week before the start of the torpor paradigm.

### Torpor paradigm

To set-up and assess the torpor paradigm, animals were housed in calorimetric cages (TSE, Bad Homburg, Germany) to measure metabolic rate (VO_2_ in mL/h) in parallel with core body temperature (T_b_). In subsequent experiments, only T_b_ was measured. On day 1, animals were housed at an ambient temperature (T_a_) of 19 °C. On day 2, animals received a single food pellet of 1.5 g at 9:00. At 17:00, the remainder of the pellet was removed. Mice typically entered torpor the second night of fasting of the paradigm. Mice were euthanized at the following time points: the pre-torpor group; PT was sacrificed at ~ 22:00 on day 3. Pre-torpor is defined as the phase just before hypothermia and hypo-metabolism are instigated, when animals are still active and have a T_b_ > 36 °C and an oxygen consumption of > 120 mL/h VO_2_. The remainder of the animals were kept on food deprivation till 12:00 on day 4, and were sacrificed at: ~ 10:00 (torpor late group; TL), ~ 12:00 (arousal early group; AE) or ~ 16:00 (arousal late group; AL). All TL, AE and AL mice underwent a torpor bout of at least 6 h with a T_b_ < 26 °C. The early arousal phase was defined by a T_b_ of ~ 30 °C. The late arousal phase was demarcated by mice being fully aroused and having reached a T_b_ > 36 °C for ~ 2 h. AE and AL mice spontaneously exited torpor and reached normal T_b_ of > 36 °C with normal VO_2_ consumption of > 80 mL/h (Fig. S1B). Visually, AE mice showed increased motor function and awareness compared to torpid mice but were not yet fully active, whereas AL animals showed normal activity.

Euthermic (EU) control mice were maintained on food ad libitum at a T_a_ of ~ 21 °C and sacrificed at ~ 13:00 on day 4 of the torpor paradigm. Mice that did not enter torpor (torpor insensitive mice) were sacrificed at ~ 13:00 and used as metabolic controls in the LTP experiment. Torpor semi-sensitive mice, mice that lowered T_b_ less than 6 h or showed highly oscillating patterns, were removed from the experiments. All mice were euthanized using decapitation or perfusion under tribromoethanol anesthesia. The brain was processed as detailed below. APP/PS1 mice showed similar profound and steady torpor bouts after fasting and no differences with wildtype mice were observed. However, slightly lower (~ 60%) success rates were found in APP/PS1 mice and their wildtype control littermates, probably due to their higher body weight.

### Golgi-Cox staining

Brains were dissected and rinsed with double distilled water (ddH_2_O). Golgi-Cox staining was performed using the Rapid GolgiStain™ Kit (FD Neurotechnologies INC, Maryland, USA) based on the principles described in^45^. All steps were performed according to the manufacturer’s instructions. After the staining procedure, brains were snap frozen using − 80 °C isopentane and coronal sections were sliced at 100 µM using a cryostat (− 20 °C; Leica). Sections were kept in ddH_2_O and then rinsed 2 × with ddH_2_O (2 × 4 min). Thereafter they were put in working solution (prepared as described by manufacturer) for 10 min. After rinsing twice for 4 min with ddH_2_O, sections were dehydrated in 4 steps: 1 × 4 min 50% ethanol, 1 × 4 min 75% ethanol, 1 × 4 min 95%, 4 × 4 min 100% ethanol. Subsequently, sections were rinsed for 2 × 4 min in xylene solution and mounted on slides using Permount and kept in the dark. Subsequent imaging and image processing were performed blinded for the experimental conditions.

### Confocal microscopy

Neurons in the CA1 area of the hippocampus were imaged using confocal laser scanning microscopy (Zeiss, LSM510) with images acquired at 10 × (0.3 NA) and 40 × (1.3 NA; immersion oil objective). The crop function in the LSM510 software was used to focus on selected areas (2 × zoom for 10 × images; 4 × zoom for 40 × images). Z-stacks were generated and processed in Fiji software^46^. Inverted images were uploaded in Neuronstudio^47^ for automated analysis. Dendrite length and branching was determined using the Scholl analysis function in Neuronstudio on 10 × CA1 overview images. 40 × images were used for automated spine numbers and spine head diameter measurement on selected 10 µm sections of the most distal ends of the first oblique (apical) dendrite and of the basal dendrite that was closest to the oblique dendrite.

### Long-term potentiation

Field long-term potentiation (LTP) was recorded using a planar multi-electrode recording setup (MED64 system; Alpha Med Sciences, Tokyo, Japan). Animals were decapitated and brains were immediately placed in ice-cold slicing buffer (124 mM NaCl, 3.3 mM KCl, 1.2 mM KH_2_PO_4_, 7 mM MgSO_4_, 0.5 mM CaCl_2_, 20 mM NaHCO_3_ and 10 mM glucose; constantly gassed with 95% O_2_/5% CO_2_). Coronal hippocampal slices were cut using a vibrating microtome at 400 µM and then placed in a chamber containing artificial cerebrospinal fluid (aCSF: 124 mM NaCl, 3.3 mM KCl, 1.2 mM KHPO_4_, 1.3 mM MgSO_4_, 2.4 mM CaCl_2_, 20 mM NaHCO_3_ and 10 mM glucose; constantly gassed with 95% O_2_/5% CO_2_). Slices were left in the buffer for at least 30 min before recording. The slices were placed on an 8 × 8 multi-electrode array containing P5155 probes (Alpha Med Sciences; inter-electrode distance 150 µM) and 500 µL aCSF was added to the moist chamber which was constantly gassed with 95%O_2_/5% CO_2_. Correct placement of the array over the CA1 area was done using a microscope (SZ61, Olympus, Japan) and an image of the placement was acquired for all the recorded slices. Slices were held in place using a platinum harp. During recording, the chamber with the slice was constantly perfused with oxygenated aCSF at flow rate 2 mL/min at RT. From the 64 electrodes, one electrode on the afferent side of the CA1 area was chosen for stimulation. All other electrodes recorded, but only the electrodes that were in the direct lane of stimulation were used for analysis (5–8 electrodes per recording). Slices with LTP measurements below baseline level of 100% were excluded and numbers did not differ between groups (data not shown). After maintaining a stable baseline recording for at least 10 min, LTP was evoked by 3 × 100 Hz stimulation (tetanus) of 1 s separated by 20 s and the slope and amplitude were measured for 60 min. LTP was expressed as percentage of baseline. All data collection and processing were performed blinded.

### Contextual fear conditioning

Mice were handled for 2 min on 2 consecutive days prior to conditioning. Context training was performed in a plexiglass chamber with a stainless steel grid floor in a sound-proof setup (Noldus). Mice were placed in the context and after 2 min received a 2 s 0.7 mA shock. Mice were kept in the context training box for an additional 30 s. In between mice, the box was thoroughly cleaned using 70% ethanol. During training, white noise was present. Context-dependent memory was assessed 24 h, 48 h, 72 h and 96 h later in the same context (including white noise) by measuring freezing for 120 s. Lastly, a novel context (a triangular shaped plastic box without grid) was used to asses generalized fear 24 h after the context test. Mice were put in the novel context without white noise and freezing levels were measured for 120 s. Between mice, the novel context box was cleaned using 0.1% acetic acid. Freezing was recorded and analyzed using Ethovision XT software (Noldus). Freezing was defined as absence of movement, including nose movement, except for respiration or heartbeat movement, and expressed as percentage of time.

### Synapse enrichment for proteomics

Hippocampal tissue was homogenized in ice-cold homogenization buffer (0.32 M sucrose, 5 mM HEPES/NaOH, PH 7.4) containing 1 tablet of cOmplete™ EDTA-free Protease Inhibitor Cocktail per 50 mL (Sigma-Aaldrich) and 1 tablet of PhosStop per 10 mL (Sigma-Aaldrich). The homogenate was then centrifuged at 1000 g for 10 min at 4 °C. The supernatant was removed and centrifuged at 20,000 g for 20 min to obtain a synapse-enriched pellet (P2).

### Protein digestion

P2 protein concentrations were determined using Bradford assay and 25 µg protein per animal was used for FASP in-solution digestion as previously described^48^. In brief, samples were incubated with 120 µL reducing agent (2% SDS, 100 mM TRIS, 1.33 mM TBEP) at 55 °C for 1 h while shaking constantly at 900 rpm. Samples were then incubated with 2 µL methyl methanethiosulfonate for 15 min at RT while shaking. The samples were then loaded onto YM-30 filters (Microcon, Millipore) and 250 µL 8 M Urea in 100 mM Tris (pH 8.8) was added. The samples were washed by spinning the filters at 14,000 × g for 10 min followed by washing with fresh Urea for 4x. Finally, the samples were washed with 50 mM NH_4_HCO_3_. After the washing steps the samples were incubated with trypsin overnight in a humidified chamber at 37 °C. The peptides were eluted form the filter using 0.1% acetic acid, dried in a SpeedVac and stored at -20 °C.

### Liquid chromatography and mass spectrometry

Peptides were quantified by LC–MS/MS using an Ultimate 3000 LC system (Dionex, Thermo Scientific) coupled to a Triple TOF 5600 mass spectrometer (Sciex). Peptides were trapped on a 5 mm Pepmap 100 C18 column (300 µm i.d., 5 µm particle size, Dionex) and fractionated on a 200 mm Altima C18 column (100 µm i.d., 3 µm particle size). The concentration of acetonitrile in the mobile phase was increased from 5–18% in 88 min, 18–25% in 98 min, 25–40% in 108 min and to 90% in 2 min at a flow rate of 5 µL/min. Eluted peptides were electro-sprayed into the TripleTOF MS using a microspray needle of 5500 V. SWATH experiments consisted of a parent ion scan of 150 ms followed by a SWATH window of 8 Da with a scan time of 80 ms. It was stepped through the mass range between 450 and 770 m/z. The collision energy per window was based on the appropriate collision energy for 2 + ions centered upon the window with a spread of 15 eV.

### SWATH data analysis

SWATH data were searched against a spectral library of P2 biochemical subfractions from mouse hippocampus using Spectronaut 13.7^49^ with default settings. The resulting abundance values and qualitative scores for each peptide in the spectral library were exported for further downstream analysis. MS-DAP 0.2.5 (https://github.com/ftwkoopmans/msdap) was used for the interpretation of data quality and differential expression analysis (DEA). While importing the Spectronaut data report, fragment group MS2 total peak areas without Spectronaut normalization were selected to represent peptide intensity values and both proteins from the MaxQuant contaminant database and iRT peptides were removed from the dataset. Samples with demonstrable chromatographic aberrations, leading to substantially increased within-group coefficient of variation estimates, were highlighted in quality control figures and excluded from differential testing.

In each statistical contrast, peptides observed in both sample groups with Spectronaut confidence score ≤ 0.01 in at least 3 samples (biological replicates) were selected. Normalization was then applied to this data subset and finally MS-EmpiRe^50^ was used for differential testing. All data visualizations and MS-DAP parameters are included in the MS-DAP report (Supplementary Dataset 3). All raw protein data is provided in Supplemental Dataset 1.

### Immunoblotting

SDS sample-buffer (Laemmli) was added to P2 fractions and samples were heated to 96 °C for 5 min. 10 µg sample was loaded on a Criterion TGX stain-free gel (BioRad). Total protein loading was determined using protein activation imaging (Gel-Doc EZ system, BioRad) and analyses with ImageLab 6.0.1 (BioRad). After transfer onto a PVDF membrane (overnight, 40 V, 4 °C), blots were incubated with primary antibodies against SHISA6 (Genscript, rabbit, 1:500), CAMK2a (Thermo Scientific, mouse, 1:4000), GluA1 (Abcam, rabbit, 1:10.000), Tau-5 (Abcam, mouse, 1:500) overnight at 4 °C, and subsequently with secondary antibodies (Sigma-Aaldrich, Gt-antiRb and Gt-antiMS HRP; 1:10.000) 2 h at RT. Blots were scanned with an Odyssey Fc System (Li-Cor) after ECL incubation (2 min) and analyzed using ImageStudio Lite 5.3 (Li-Cor). Total protein loading was used for normalization.

### Statistics

GraphPad Prism 8.02 (for Windows, GraphPad Software, La Jolla, CA) was used for statistical analyses. For all statistical tests, a *p* or* q* (FDR correction in proteomics data) < 0.05 was considered significant. Error bars show the standard error of the mean (SEM). The number of neurons or animals used for statistical analysis are indicated in all graphs, either numerical or by showing all data points. For pairwise comparisons the Students *t*-test was used. For comparisons of three or more groups an ANOVA was used with a post-hoc Fisher’s LSD test (3 groups), Holm-Sidak post-hoc test (4 groups) or Tukey post-hoc test (5 groups or more). Inclusion criteria for deep and steady torpor are described above and in Fig. S1.

## Supplementary Information

Supplementary Information 1.

Supplementary Information 2.

Supplementary Information 3.

Supplementary Information 4.
