# Supplementary material for: Torpor enhances synaptic strength and restores memory performance in a mouse model of Alzheimer’s disease
Source: Sci Rep. 2021 Jul 29;11:15486. doi: 10.1038/s41598-021-94992-x (PMC8322095; doi:10.1038/s41598-021-94992-x)
Supplement: Supplementary file 1 — Supplementary Information 1. [file 41598_2021_94992_MOESM1_ESM.pdf]

Supplementary Information

Torpor enhances synaptic strength and restores memory performance in a mouse model of Alzheimer's disease

Christina F. de Veij Mestdagh[1,2], Jaap A. Timmerman[3], Frank Koopmans[1], Iryna Paliukhovich[1], Suzanne S. M. Miedema[1], Maaike Goris[2], Rolinka J. van der Loo[1], Guido Krenning[4,5], Ka Wan Li[1], Huibert D. Mansvelder[3], August B. Smit[1], Robert H. Henning[2] and Ronald E. van Kesteren[1]*

[1]Department of Molecular and Cellular Neurobiology, Center for Neurogenomics and Cognitive Research, VU University, Amsterdam, the Netherlands

[2]Department of Clinical Pharmacy and Pharmacology, University Medical Center Groningen, Groningen, the Netherlands

[3]Department of Integrative Neurophysiology, Center for Neurogenomics and Cognitive Research, VU University, Amsterdam, the Netherlands

[4]Department of Pathology and Medical Biology, University Medical Center Groningen, Groningen, the Netherlands

[5]Sulfateq B.V., Groningen, The Netherlands

*Corresponding author

E-mail: ronald.van.kesteren@vu.nl (REvK)

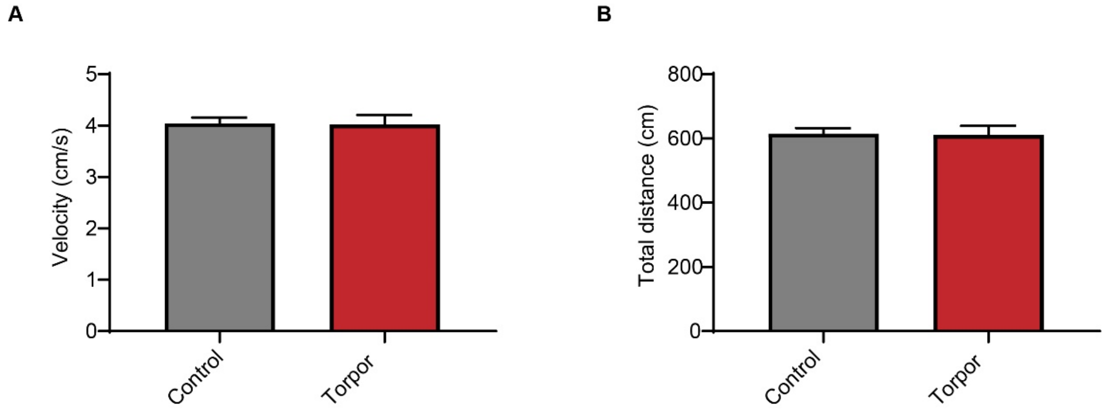

Figure S1: No differences in velocity or distance moved between control and arousal mice. (**A**) The mean velocity during memory acquisition in control mice vs arousal mice was not different (4.043 ± 0.11 vs 4.024 ± 0.18). (**B**) The same holds for total distance moved during 2 min of context exploration (614.9 ± 17.5 vs 611.3 ± 28.0).

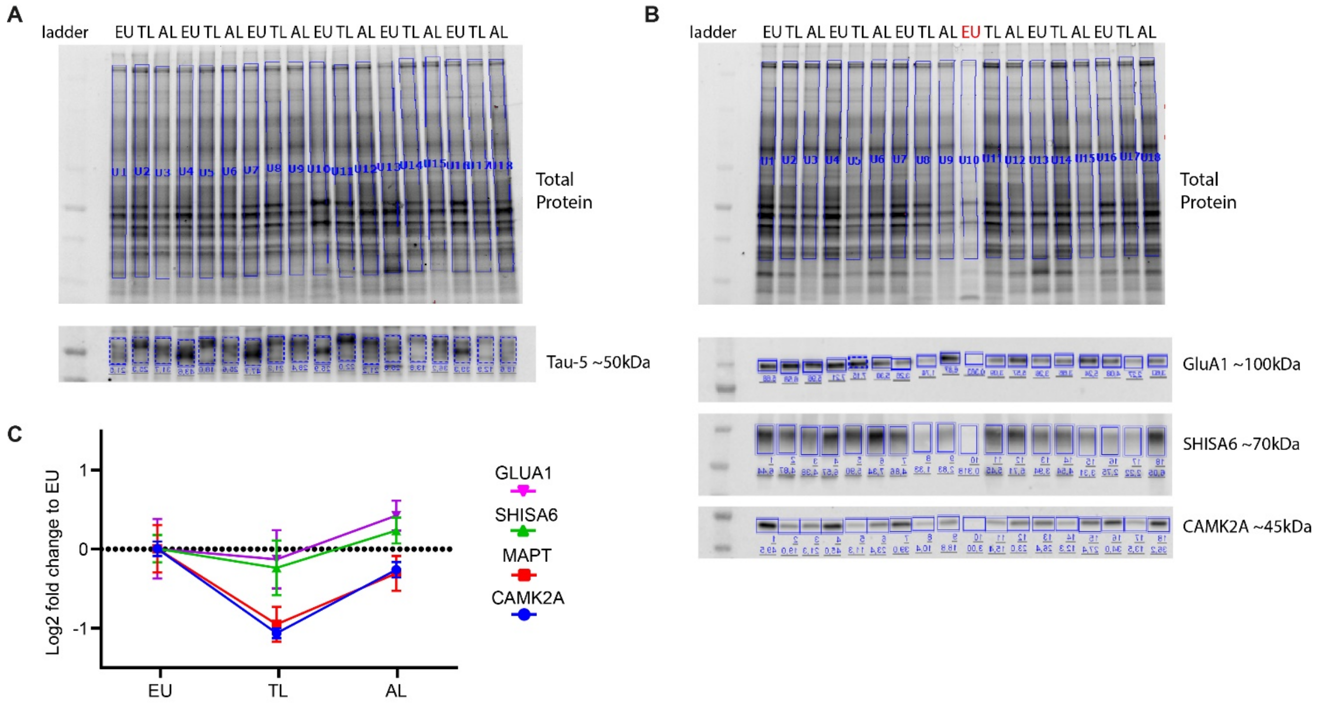

Figure S2: Immunoblotting confirms protein regulation measured by MS. (**A**) Gel and immunoblot images used for tau-5 protein level determination. (**B**) Gel and immunoblot images used for GluA1, SHISA6 and CAMK2A protein level determination. One EU sample (red) was excluded due to a loading error. (**C**) Quantification of immunoblot data for GluA1 (pink), SHISA6 (green), MAPT (blue) and CAMK2a (red) (n = 5-6 / group).

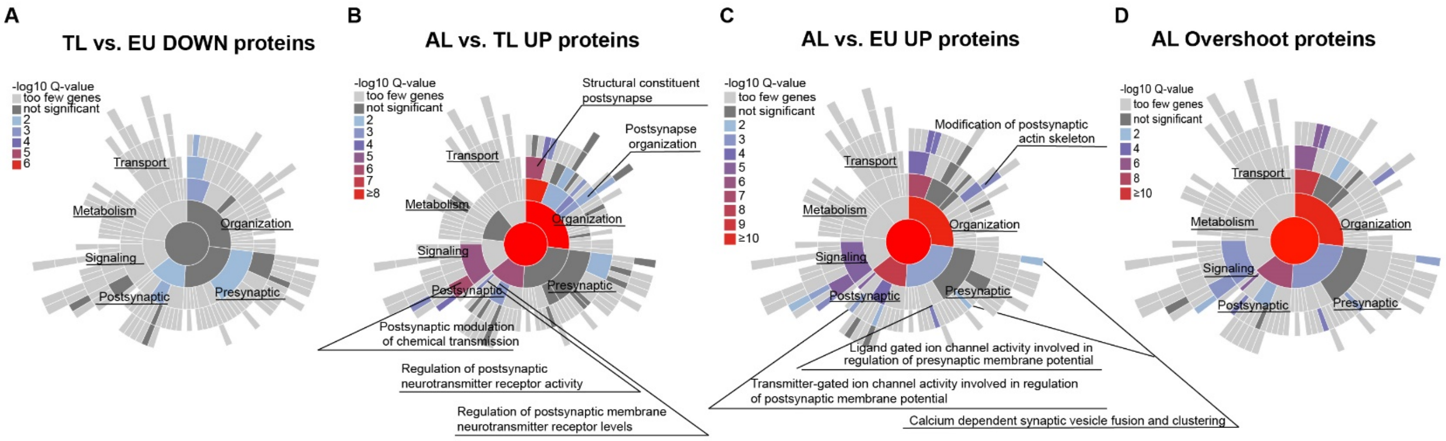

Figure S3: Specific down- and upregulation of synaptic plasticity proteins during torpor. Functional annotation and enrichment of synaptic proteins were determined with SynGO. (**A-D**) Postsynaptic and postsynaptic organization related Biological Process terms are significantly enriched in AL vs. TL, Al vs. EU and AL overshoot proteins. All enriched terms and annotated proteins are shown in tables S5-8.

Table S1: TL vs. EU enriched Cellular Component GO terms and annotated proteins

CC GO term	Annotated proteins
synapse	SLC6A11;BIN1;TPD52;QKI;SLC3A2;BCAS1;STXBP3;VAMP1;AAK1;CASKIN1;CAMK2A;NEFM;NEFL;SNAP47;BSN;PCLO;ATP2B2;SYT11;MYO6;ANXA5;RPH3A;SLC32A1;SYT2;SNAP25;KCNA1;ATP1A3;SNAP23;ADAM10;SPTBN1;INA;AGAP2;NEFH;DLGAP4;GPHN;CAMK2B;CTNND2;RAPGEF4;IQSEC1;DLG1;CNKSR2;PLPPR4;IQSEC3;CNTN2;NTRK3
postsynaptic intermediate filament cytoskeleton	INA;NEFM;NEFL;NEFH

Table S2: AL vs. TL enriched Cellular Component GO terms and annotated proteins

CC GO term	Annotated proteins
synapse	PABPC1;RPL8;RPL7;VCAN;ARPC2;CAPRIN1;PPFIA3;PPP3CA;RTN3;CLU;GPC1;ARFGAP1;BCAS1;RPL13;RPL13A;RPS28;RPS5;PPP1R9A;SPTBN2;KALRN;AAK1;ADD1;ADD2;ADD3;CASKIN1;PTK2B;PRKCB;FMR1;BAIAP2;CAMK2A;NEFM;NEFL;UNC13A;ARHGAP44;VPS11;RIMS1;BSN;PCLO;RIMBP2;ERC1;ERC2;IQSEC2;VDAC1;GRIA2;GRIA1;GRIN1;GRIN2B;MYO6;ATP6V1E1;ATP6V1G2;SYN1;SYN2;RPH3A;SNAP25;EPHA4;CACNA1E;PTPRS;GRIN2A;PTPRD;SNAP23;ADAM10;BEGAIN;FXR1;WASF1;SPTBN1;HNRNPK;INA;WASL;ARHGAP39;DNAJB1;MARK2;SPTB;SPTAN1;AGAP2;CTTN;ARHGEF2;HOMER1;NEFH;CTTNBP2;SYNE1;ITPKA;SYNPO;DLGAP3;DLGAP4;GABRA4;SHISA7;NLGN1;NLGN3;KCNB1;HOMER3;ABI1;SHANK2;SHANK3;SHANK1;CRTC1;CAMK2B;PPP1R9B;CTNND2;EIF4G2;HNRNPH1;DGKZ;RAPGEF4;AGAP3;CDKL5;SYNGAP1;IQSEC1;DLGAP1;ACTN2;DLG3;PSD;DLG2;DLG4;CNKSR2;PLPPR4;CACNG8;CNIH2;LRRC4B;LRRC4C;LRRC7;SHISA6;GRM5;NTRK3;DAGLA;PRRT1;TNC
presynapse	PPP1R9A;SPTBN2;KALRN;AAK1;ADD1;ADD2;ADD3;CASKIN1;PTK2B;PRKCB;FMR1;BAIAP2;CAMK2A;NEFM;NEFL;UNC13A;ARHGAP44;VPS11;RIMS1;BSN;PCLO;RIMBP2;ERC1;ERC2;IQSEC2;PPFIA3;VDAC1;GRIA2;GRIA1;GRIN1;GRIN2B;MYO6;ATP6V1E1;ATP6V1G2;SYN1;SYN2;RPH3A;SNAP25;EPHA4;CACNA1E;PTPRS;GRIN2A;PTPRD;RPL13;RPL13A;RPL7;RPL8;RPS28;RPS5
presynaptic active zone	UNC13A;ARHGAP44;VPS11;RIMS1;BSN;PCLO;RIMBP2;ERC1;ERC2;IQSEC2;PPFIA3;VDAC1;GRIA2;GRIA1;GRIN1;GRIN2B
presynaptic active zone cytoplasmic component	RIMS1;BSN;PCLO;RIMBP2;ERC1;ERC2;IQSEC2;PPFIA3
postsynapse	SNAP23;ADAM10;BEGAIN;FXR1;WASF1;CAPRIN1;RPL8;SPTBN1;HNRNPK;INA;PTK2B;WASL;ADD2;ADD1;ADD3;ARHGAP39;DNAJB1;MARK2;SPTB;SPTAN1;AGAP2;CTTN;ARHGEF2;FMR1;HOMER1;BAIAP2;CAMK2A;NEFM;NEFL;NEFH;CTTNBP2;PPP1R9A;MYO6;SYNE1;ITPKA;SPTBN2;SYNPO;DLGAP3;DLGAP4;GABRA4;SHISA7;NLGN1;NLGN3;KCNB1;HOMER3;ABI1;SHANK2;SHANK3;SHANK1;CRTC1;CAMK2B;PPP1R9B;CTNND2;EIF4G2;RPL7;HNRNPH1;ARHGAP44;DGKZ;KALRN;RAPGEF4;AGAP3;CDKL5;SYNGAP1;IQSEC2;IQSEC1;DLGAP1;ACTN2;DLG3;PSD;DLG2;DLG4;CNKSR2;GRIA1;PLPPR4;GRIA2;GRIN1;GRIN2B;CACNG8;CNIH2;LRRC4B;LRRC4C;LRRC7;SHISA6;GRIN2A;VDAC1;GRM5;EPHA4;CACNA1E;NTRK3;DAGLA;PRRT1;RPL13;RPL13A;RPS28;RPS5
postsynaptic cytosol	FMR1;HOMER1;BAIAP2;CAMK2A
postsynaptic cytoskeleton	INA;NEFM;NEFL;NEFH;CTTN;CTTNBP2;PPP1R9A;MYO6;SYNE1;ITPKA;SPTBN2
postsynaptic intermediate filament cytoskeleton	INA;NEFM;NEFL;NEFH
postsynaptic actin cytoskeleton	CTTN;CTTNBP2;PPP1R9A;MYO6;SYNE1;ITPKA
postsynaptic specialization	DLGAP3;DLGAP4;GABRA4;SHISA7;NLGN1;NLGN3;KCNB1;HOMER1;HOMER3;ABI1;SHANK2;SHANK3;SHANK1;CRTC1;CAMK2B;CAMK2A;PPP1R9A;PPP1R9B;MYO6;CTNND2;EIF4G2;RPL8;RPL7;HNRNPH1;DNAJB1;ARHGAP44;DGKZ;KALRN;RAPGEF4;AGAP3;CDKL5;SYNGAP1;BAIAP2;IQSEC2;IQSEC1;DLGAP1;ARHGEF2;ACTN2;DLG3;PSD;INA;PTK2B;DLG2;DLG4;CNKSR2;GRIA1;PLPPR4;GRIA2;GRIN1;GRIN2B;CACNG8;CNIH2;LRRC4B;LRRC4C;LRRC7;SHISA6;GRIN2A;VDAC1
integral component of postsynaptic specialization membrane	GABRA4;SHISA7;NLGN1;NLGN3;KCNB1
postsynaptic density	HOMER1;HOMER3;ABI1;SHANK2;SHANK3;SHANK1;CRTC1;CAMK2B;CAMK2A;PPP1R9A;PPP1R9B;MYO6;CTNND2;EIF4G2;RPL8;RPL7;HNRNPH1

	;DNAJB1;ARHGAP44;DGKZ;KALRN;RAPGEF4;AGAP3;CDKL5;SYNGAP1;BAIAP2;IQSEC2;IQSEC1;DLGAP1;ARHGEF2;ACTN2;DLG3;PSD;INA;PTK2B;DLG2;DLG4;CNKSR2;GRIA1;PLPPR4;GRIA2;GRIN1;GRIN2B;CACNG8;CNIH2;LRRC4B;LRRC4C;LRRC7;SHISA6;GRIN2A;VDAC1
postsynaptic density, intracellular component	CDKL5;SHANK3;SYNGAP1;BAIAP2;IQSEC2;IQSEC1;DLGAP1;ARHGEF2;ACTN2;DLG3;PSD;INA;PTK2B
postsynaptic density membrane	IQSEC2;ACTN2;DLG2;DLG4;CNKSR2;GRIA1;PLPPR4;GRIA2;GRIN1;GRIN2B;CACNG8;CNIH2;LRRC4B;LRRC4C;LRRC7;SHISA6;GRIN2A;VDAC1
integral component of postsynaptic density membrane	GRIA1;PLPPR4;GRIA2;GRIN1;GRIN2B;CACNG8;CNIH2;LRRC4B;LRRC4C;LRRC7;SHISA6;GRIN2A;VDAC1

Table S3: AL vs. TL enriched Cellular Component GO terms and annotated proteins

CC GO term	Annotated proteins
synapse	PPP3R1;CYFIP2;PABPC1;RPS9;NCAN;PTPRZ1;ARPC2;PPP3CA;PPP3CB;RTN3;GPC1;YWHAE;RPS5;RPS27;SYNJ1;PFN1;SPTBN2;KALRN;ADD1;ADD2;PTK2B;PRKCE;ANKS1B;RIMS1;BSN;PCLO;RIMBP2;ERC2;IQSEC2;VDAC1;GRIA2;GRIA1;GRIN1;GRIN2B;KCNMA1;DNM1;DMXL2;ATP6V1E1;ATP6V1G2;EPHA4;GRIN2A;NPTX1;BEGAIN;WASF1;ACTR2;ACTR3;SPTBN1;HNRNPK;CAMKV;HPCA;WASL;SPTB;SPTAN1;CTTN;ARHGEF2;HOMER1;DBN1;ITPKA;NLGN3;HOMER3;ABI1;SHANK2;SHANK1;PPP1R9B;CAPZB;SYNGAP1;DLGAP1;DLG3;DLG2;DLG4;PLPPR4;CACNG8;CNIH2;LRRC4B;LRRC7;SHISA6;SLC8A2;DAGLA;PRRT1;BCAN;TNC
presynapse	RPS27;SYNJ1;PFN1;SPTBN2;KALRN;ADD1;ADD2;PTK2B;PRKCE;ANKS1B;RIMS1;BSN;PCLO;RIMBP2;ERC2;IQSEC2;VDAC1;GRIA2;GRIA1;GRIN1;GRIN2B;KCNMA1;DNM1;DMXL2;ATP6V1E1;ATP6V1G2;EPHA4;GRIN2A;RPS5
presynaptic active zone	RIMS1;BSN;PCLO;RIMBP2;ERC2;IQSEC2;VDAC1;GRIA2;GRIA1;GRIN1;GRIN2B;KCNMA1
presynaptic active zone cytoplasmic component	RIMS1;BSN;PCLO;RIMBP2;ERC2;IQSEC2
postsynapse	BEGAIN;WASF1;PFN1;ACTR2;ACTR3;SPTBN1;HNRNPK;CAMKV;HPCA;PTK2B;WASL;ADD2;ADD1;SPTB;SPTAN1;CTTN;ARHGEF2;HOMER1;DBN1;ITPKA;SPTBN2;NLGN3;HOMER3;ABI1;SHANK2;SHANK1;ANKS1B;PPP1R9B;RPS27;KALRN;CAPZB;SYNGAP1;IQSEC2;DLGAP1;DLG3;DLG2;DLG4;GRIA1;PLPPR4;GRIA2;GRIN1;GRIN2B;CACNG8;CNIH2;LRRC4B;LRRC7;PTPRZ1;SHISA6;GRIN2A;VDAC1;EPHA4;SLC8A2;DAGLA;PRRT1;RPS5;RPS9
postsynaptic specialization	NLGN3;HOMER1;HOMER3;ABI1;SHANK2;SHANK1;ANKS1B;PPP1R9B;RPS27;DBN1;KALRN;CAPZB;SYNGAP1;IQSEC2;DLGAP1;ARHGEF2;DLG3;PTK2B;DLG2;DLG4;GRIA1;PLPPR4;GRIA2;GRIN1;GRIN2B;CACNG8;CNIH2;LRRC4B;LRRC7;PTPRZ1;SHISA6;GRIN2A;VDAC1
postsynaptic density	HOMER1;HOMER3;ABI1;SHANK2;SHANK1;ANKS1B;PPP1R9B;RPS27;DBN1;KALRN;CAPZB;SYNGAP1;IQSEC2;DLGAP1;ARHGEF2;DLG3;PTK2B;DLG2;DLG4;GRIA1;PLPPR4;GRIA2;GRIN1;GRIN2B;CACNG8;CNIH2;LRRC4B;LRRC7;PTPRZ1;SHISA6;GRIN2A;VDAC1
postsynaptic density, intracellular component	SYNGAP1;IQSEC2;ANKS1B;DLGAP1;ARHGEF2;DLG3;PTK2B
postsynaptic density membrane	IQSEC2;DLG2;DLG4;GRIA1;PLPPR4;GRIA2;GRIN1;GRIN2B;CACNG8;CNIH2;LRRC4B;LRRC7;PTPRZ1;SHISA6;GRIN2A;VDAC1
integral component of postsynaptic density membrane	GRIA1;PLPPR4;GRIA2;GRIN1;GRIN2B;CACNG8;CNIH2;LRRC4B;LRRC7;PTPRZ1;SHISA6;GRIN2A;VDAC1

Table S4: AL overshoot enriched Cellular Component GO terms and annotated proteins

CC GO term	Annotated proteins
synapse	PABPC1;RPS7;ARPC2;PPP3CA;RTN3;GPC1;RPS5;SPTBN2;KALRN ;ADD1;ADD2;PTK2B;RIMS1;BSN;PCLO;RIMBP2;ERC2;IQSEC2 ;VDAC1;GRIA2;GRIA1;GRIN1;GRIN2B;ATP6V1E1;ATP6V1G2;EPHA4 ;GRIN2A;BEGAIN;WASF1;SPTBN1;HNRNPK;WASL;SPTB;SPTAN1 ;CTTN;ARHGEF2;HOMER1;ITPKA;NLGN3;HOMER3;ABI1;SHANK2; SHANK1;PPP1R9B;SYNGAP1;DLGAP1;DLG3;DLG2;DLG4;PLPPR4 ;CACNG8;CNIH2;LRRC4B;LRRC7;SHISA6;DAGLA;PRRT1;TNC
presynapse	SPTBN2;KALRN;ADD1;ADD2;PTK2B;RIMS1;BSN;PCLO;RIMBP2; ERC2;IQSEC2;VDAC1;GRIA2;GRIA1;GRIN1;GRIN2B;ATP6V1E1; ATP6V1G2;EPHA4;GRIN2A;RPS5
presynaptic active zone	RIMS1;BSN;PCLO;RIMBP2;ERC2;IQSEC2;VDAC1;GRIA2;GRIA1 ;GRIN1;GRIN2B
presynaptic active zone cytoplasmic component	RIMS1;BSN;PCLO;RIMBP2;ERC2;IQSEC2
presynaptic active zone membrane	VDAC1;GRIA2;GRIA1;GRIN1;GRIN2B
integral component of presynaptic membrane	EPHA4;GRIA1;GRIA2;GRIN1;GRIN2A;GRIN2B
postsynapse	BEGAIN;WASF1;SPTBN1;HNRNPK;PTK2B;WASL;ADD2;ADD1;SPTB; SPTAN1;CTTN;ARHGEF2;HOMER1;ITPKA;SPTBN2;NLGN3;HOMER3 ;ABI1;SHANK2;SHANK1;PPP1R9B;KALRN;SYNGAP1;IQSEC2 ;DLGAP1;DLG3;DLG2;DLG4;GRIA1;PLPPR4;GRIA2;GRIN1;GRIN2B; CACNG8;CNIH2;LRRC4B;LRRC7;SHISA6;GRIN2A;VDAC1;EPHA4; DAGLA;PRRT1;RPS5;RPS7
postsynaptic specialization	NLGN3;HOMER1;HOMER3;ABI1;SHANK2;SHANK1;PPP1R9B;KALRN ;SYNGAP1;IQSEC2;DLGAP1;ARHGEF2;DLG3;PTK2B;DLG2;DLG4; GRIA1;PLPPR4;GRIA2;GRIN1;GRIN2B;CACNG8;CNIH2;LRRC4B; LRRC7;SHISA6;GRIN2A;VDAC1
postsynaptic density	HOMER1;HOMER3;ABI1;SHANK2;SHANK1;PPP1R9B;KALRN ;SYNGAP1;IQSEC2;DLGAP1;ARHGEF2;DLG3;PTK2B;DLG2;DLG4; GRIA1;PLPPR4;GRIA2;GRIN1;GRIN2B;CACNG8;CNIH2;LRRC4B; LRRC7;SHISA6;GRIN2A;VDAC1
postsynaptic density, intracellular component	SYNGAP1;IQSEC2;DLGAP1;ARHGEF2;DLG3;PTK2B
postsynaptic density membrane	IQSEC2;DLG2;DLG4;GRIA1;PLPPR4;GRIA2;GRIN1;GRIN2B ;CACNG8;CNIH2;LRRC4B;LRRC7;SHISA6;GRIN2A;VDAC1
integral component of postsynaptic density membrane	GRIA1;PLPPR4;GRIA2;GRIN1;GRIN2B;CACNG8;CNIH2;LRRC4B; LRRC7;SHISA6;GRIN2A;VDAC1

Table S5: TL vs. AL enriched Biological Process GO terms and annotated proteins

BP GO term	Annotated proteins
synaptic vesicle cycle	BSN;RAPGEF4;PCLO;SNAP25;STX1B;SYT2;CAMK2A;VAMP1;STXBP1; RPH3A;SLC32A1;BIN1;SYT11
process in the postsynapse	ATP2B2;KCNA1;CTNND2;GPHN;DLG1;ADAM10;CAMK2A;RAPGEF4; SNAP23;SNAP47;MYO6;IQSEC1
regulation of postsynaptic membrane neurotransmitter receptor levels	CTNND2;GPHN;DLG1;ADAM10;CAMK2A;RAPGEF4;SNAP23;SNAP47 ;MYO6;IQSEC1
structural constituent of synapse	PCLO;BSN;CAMK2B;INA;NEFL;NEFH;DLG1;CTNND2
structural constituent of postsynapse	CAMK2B;INA;NEFL;NEFH;DLG1;CTNND2
structural constituent of postsynaptic intermediate filament cytoskeleton	INA;NEFL;NEFH

Table S6: AL vs. TL enriched Biological Process GO terms and annotated proteins

BP GO term	Annotated proteins
process in the synapse	ERC1;ERC2;CACNA1E;RIMBP2;GRIA1;GRIA2;GRIN2B;GRIN2A;GRIN1;SYN1;SYN2;BSN;RAPGEF4;PCLO;ABI1;SNAP25;UNC13A;PPFIA3;RIMS1;PRKCB;CAMK2A;RPH3A;ATP6V1G2;ATP6V1E1;SYNPO;GABRA4;HOMER1;HOMER3;BEGAIN;DLGAP2;CNIH2;SHISA7;SHISA6;DLG3;CACNG8;CTNND2;AGAP3;DLG2;DLG4;IQSEC2;ADAM10;KALRN;SNAP23;MYO6;IQSEC1;SYNE1;DAGLA;PLCB1;PTPRD;BAIAP2;HNRNPK;PPP1R9A;GRM5;LRRC4C;PTPRS;CDKL5;SYNGAP1;SHANK1;SHANK3;DLGAP1;DLGAP3;ARHGAP44;PAFAH1B1;PPP3CA;INA;NEFL;NEFH;PTK2B;NLGN1;SPTBN2;CNKSR2;ARHGAP39;WASL;SHANK2;MARK2;CTTN;WASF1;ITPKA;SPTB;CTTNBP2;ACTN1;CAMK2B;LRRC4B;ADD2;SRCIN1;NTRK3;PSD;NLGN3;EPHA4;DGKZ;ARHGEF7;ROCK2;FMR1;RPL13;RPL13A;RPL7;RPL8;RPS28;RPS5
synaptic vesicle exocytosis	SNAP25;UNC13A;PPFIA3;RIMS1;RIMBP2;ERC1;ERC2;PRKCB;CACNA1E;CAMK2A;RPH3A
process in the postsynapse	SYNPO;GRIN1;GRIN2A;GRIN2B;GRIA2;GABRA4;GRIA1;HOMER1;HOMER3;BEGAIN;DLGAP2;CNIH2;SHISA7;SHISA6;DLG3;CACNG8;CTNND2;AGAP3;DLG2;DLG4;IQSEC2;ADAM10;CAMK2A;KALRN;RAPGEF4;SNAP23;MYO6;IQSEC1;SYNE1
regulation of postsynaptic neurotransmitter receptor activity	HOMER1;HOMER3;BEGAIN;DLGAP2;CNIH2;SHISA7;SHISA6
regulation of postsynaptic membrane neurotransmitter receptor levels	DLG3;CACNG8;CTNND2;AGAP3;DLG2;DLG4;IQSEC2;ADAM10;CAMK2A;KALRN;RAPGEF4;SNAP23;SHISA6;MYO6;IQSEC1;SYNE1
trans-synaptic signaling	DAGLA;PLCB1;PTPRD;BAIAP2;IQSEC2;HNRNPK;PPP1R9A;GRM5;LRRC4C;PTPRS;CDKL5;SYNGAP1;SHANK1;SHANK3;DLGAP1;DLGAP3;DLGAP2;ARHGAP44;PAFAH1B1;MYO6;SYNPO;PPP3CA;INA;NEFL;NEFH;IQSEC1;PTK2B
chemical synaptic transmission	BAIAP2;IQSEC2;HNRNPK;PPP1R9A;GRM5;LRRC4C;PTPRD;PTPRS;CDKL5;SYNGAP1;SHANK1;SHANK3;DLGAP1;DLGAP3;DLGAP2;ARHGAP44;PAFAH1B1;MYO6;SYNPO;PLCB1;PPP3CA;INA;NEFL;NEFH;IQSEC1;PTK2B
modulation of chemical synaptic transmission	BAIAP2;IQSEC2;HNRNPK;PPP1R9A;GRM5;LRRC4C;PTPRD;PTPRS;CDKL5;SYNGAP1;SHANK1;SHANK3;DLGAP1;DLGAP3;DLGAP2;ARHGAP44;PAFAH1B1
postsynaptic modulation of chemical synaptic transmission	SYNPO;PLCB1;PPP3CA;INA;NEFL;NEFH;IQSEC1;PTK2B
synapse organization	PTPRS;NLGN1;ADAM10;SPTBN2;CNKSR2;ARHGAP39;WASL;CDKL5;HNRNPK;SHANK2;SHANK3;RAPGEF4;MARK2;BAIAP2;PAFAH1B1;DLGAP3;ARHGAP44;CTTN;WASF1;ITPKA;SPTB;CTTNBP2;KALRN;RIMS1;PCLO;RIMBP2;BSN;ERC1;ERC2;ACTN1;CAMK2B;INA;NEFL;NEFH;SHANK1;DLGAP1;DLG4;DLG3;DLG2;CTNND2;LRRC4B;PTPRD;LRRC4C;ADD2;PPFIA3;SRCIN1;NTRK3;PSD;NLGN3;PTK2B;EPHA4;SYNGAP1;DGKZ;ABI1;ARHGEF7;PPP1R9A;ROCK2
postsynapse organization	ADAM10;SPTBN2;CNKSR2;ARHGAP39;WASL;CDKL5;HNRNPK;SHANK2;SHANK3;RAPGEF4;MARK2;BAIAP2;PAFAH1B1
regulation of postsynapse organization	CDKL5;HNRNPK;SHANK2;SHANK3;RAPGEF4;ADAM10;WASL;MARK2;BAIAP2;PAFAH1B1
modification of synaptic structure	DLGAP3;ARHGAP44;CTTN;WASF1;ITPKA;SPTB;CTTNBP2;BAIAP2;KALRN
modification of postsynaptic structure	ARHGAP44;CTTN;WASF1;ITPKA;SPTB;CTTNBP2;BAIAP2;KALRN
modification of postsynaptic actin cytoskeleton	WASF1;CTTN;ITPKA;SPTB;CTTNBP2;BAIAP2;KALRN
structural constituent of synapse	RIMS1;PCLO;RIMBP2;BSN;ERC1;ERC2;SPTBN2;ACTN1;CAMK2B;INA;NEFL;NEFH;SHANK3;SHANK1;DLGAP1;SHANK2;DLG4;DLG3;DLG2;CTNND2

structural constituent of active zone	RIMS1;PCLO;RIMBP2;BSN;ERC1;ERC2
structural constituent of postsynapse	SPTBN2;ACTN1;CAMK2B;INA;NEFL;NEFH;SHANK3;SHANK1;DLGAP1;SHANK2;DLG4;DLG3;DLG2;CTNND2
structural constituent of postsynaptic density	SHANK3;SHANK1;DLGAP1;SHANK2;DLG4;DLG3;DLG2;CTNND2
synapse assembly	ADD2;PPFIA3;PTPRD;PTPRS;SRCIN1;NTRK3;LRRC4B;PSD;NLGN1;NLGN3;SPTBN2;PTK2B
postsynaptic specialization assembly	NLGN1;NLGN3;SPTBN2;NTRK3;PTPRD;PTPRS;LRRC4B;PTK2B

Table S7: AL vs. TL enriched Biological Process GO terms and annotated proteins

BP GO term	Annotated proteins
process in the synapse	ERC2;RIMBP2;GRIA1;GRIA2;GRIN2B;GRIN2A;GRIN1;KCNMA1;BSN;PCLO;ABI1;RIMS1;ATP6V1G2;ATP6V1E1;DNM1;PPP3CB;SYNJ1;SLC8A2;HOMER1;HOMER3;BEGAIN;NPTX1;DLGAP2;CNIH2;SHISA6;YWHAE;DLG3;CACNG8;DLG2;DLG4;IQSEC2;KALRN;NBEA;PPP3R1;HPCA;DAGLA;PLCB1;HNRNPK;NCAN;SYNGAP1;SHANK1;DLGAP1;CAMKV;PFN1;PRKCE;ANKS1B;PPP3CA;PTK2B;SPTBN2;ACTR3;WASL;SHANK2;CTTN;WASF1;ITPKA;SPTB;ACTN1;LRRC4B;ADD2;NLGN3;EPHA4;BCAN;ROCK2;DBN1;RPS5;RPS27
process in the presynapse	ERC2;RIMBP2;GRIA1;GRIA2;GRIN2B;GRIN2A;GRIN1;KCNMA1;BSN;PCLO;ABI1;RIMS1;ATP6V1G2;ATP6V1E1;DNM1;PPP3CB;SYNJ1
ligand-gated ion channel activity involved in regulation of presynaptic membrane potential	GRIA1;GRIA2;GRIN2B;GRIN2A;GRIN1;KCNMA1
synaptic vesicle clustering	PCLO;ABI1;BSN
regulation of calcium-dependent activation of synaptic vesicle fusion	RIMS1;RIMBP2;ERC2
process in the postsynapse	SLC8A2;GRIN1;GRIN2A;GRIN2B;GRIA2;GRIA1;HOMER1;HOMER3;BEGAIN;NPTX1;DLGAP2;CNIH2;SHISA6;YWHAE;DLG3;CACNG8;DLG2;DLG4;IQSEC2;KALRN;NBEA;PPP3R1;HPCA;SYNJ1
transmitter-gated ion channel activity involved in regulation of postsynaptic membrane potential	GRIN1;GRIN2A;GRIN2B;GRIA2;GRIA1
regulation of postsynaptic neurotransmitter receptor activity	HOMER1;HOMER3;BEGAIN;NPTX1;DLGAP2;CNIH2;SHISA6
regulation of postsynaptic membrane neurotransmitter receptor levels	YWHAE;DLG3;CACNG8;NPTX1;DLG2;DLG4;IQSEC2;KALRN;NBEA;SHISA6;PPP3R1;HPCA;SYNJ1
neurotransmitter receptor localization to postsynaptic specialization membrane	NPTX1;DLG2;DLG4;IQSEC2;KALRN;NBEA
trans-synaptic signaling	DAGLA;PLCB1;IQSEC2;HNRNPK;NCAN;DNM1;SYNGAP1;SHANK1;DLGAP1;CAMKV;DLGAP2;PFN1;PRKCE;ANKS1B;PPP3R1;PPP3CA;PTK2B
chemical synaptic transmission	IQSEC2;HNRNPK;NCAN;DNM1;SYNGAP1;SHANK1;DLGAP1;CAMKV;DLGAP2;PFN1;PRKCE;ANKS1B;PLCB1;PPP3R1;PPP3CA;PTK2B
modulation of chemical synaptic transmission	IQSEC2;HNRNPK;NCAN;DNM1;SYNGAP1;SHANK1;DLGAP1;CAMKV;DLGAP2;PFN1
postsynaptic process involved in chemical synaptic transmission	ANKS1B;PLCB1;PPP3R1;PPP3CA;PTK2B
postsynaptic modulation of chemical synaptic transmission	PLCB1;PPP3R1;PPP3CA;PTK2B
synapse organization	NBEA;SPTBN2;ACTR3;WASL;HNRNPK;SHANK2;CTTN;CAMKV;WASF1;PFN1;ITPKA;SPTB;KALRN;RIMS1;PCLO;RIMBP2;BSN;ERC2;ACTN1;SHANK1;DLGAP1;DLG4;DLG3;DLG2;LRRC4B;ADD2;NLGN3;NPTX1;PTK2B;EPHA4;BCAN;SYNGAP1;ABI1;ROCK2;DBN1
modification of postsynaptic structure	CTTN;CAMKV;WASF1;PFN1;ITPKA;SPTB;KALRN
modification of postsynaptic actin cytoskeleton	WASF1;CTTN;PFN1;ITPKA;SPTB;KALRN
structural constituent of synapse	RIMS1;PCLO;RIMBP2;BSN;ERC2;SPTBN2;ACTN1;SHANK1;DLGAP1;SHANK2;DLG4;DLG3;DLG2
structural constituent of active zone	RIMS1;PCLO;RIMBP2;BSN;ERC2

structural constituent of postsynapse	SPTBN2;ACTN1;SHANK1;DLGAP1;SHANK2;DLG4;DLG3;DLG2
structural constituent of postsynaptic density	SHANK1;DLGAP1;SHANK2;DLG4;DLG3;DLG2

Table S8: AL overshoot enriched Biological Process GO terms and annotated proteins

BP GO term	Annotated proteins
process in the synapse	ERC2;RIMBP2;GRIA1;GRIA2;GRIN2B;GRIN2A;GRIN1;BSN;PCLO; ABI1;RIMS1;ATP6V1G2;ATP6V1E1;HOMER1;HOMER3;BEGAIN; DLGAP2;CNIH2;SHISA6;DLG3;CACNG8;DLG2;DLG4;IQSEC2 ;KALRN;DAGLA;PLCB1;HNRNPK;SYNGAP1;SHANK1;DLGAP1 ;PPP3CA;PTK2B;SPTBN2;WASL;SHANK2;CTTN;WASF1;ITPKA ;SPTB;ACTN1;LRRC4B;ADD2;NLGN3;EPHA4;ROCK2;RPS5
process in the presynapse	ERC2;RIMBP2;GRIA1;GRIA2;GRIN2B;GRIN2A;GRIN1;BSN ;PCLO;ABI1;RIMS1;ATP6V1G2;ATP6V1E1
ligand-gated ion channel activity involved in regulation of presynaptic membrane potential	GRIA1;GRIA2;GRIN2B;GRIN2A;GRIN1
synaptic vesicle clustering	PCLO;ABI1;BSN
regulation of calcium-dependent activation of synaptic vesicle fusion	RIMS1;RIMBP2;ERC2
process in the postsynapse	GRIN1;GRIN2A;GRIN2B;GRIA2;GRIA1;HOMER1;HOMER3; BEGAIN;DLGAP2;CNIH2;SHISA6;DLG3;CACNG8;DLG2;DLG4; IQSEC2;KALRN
transmitter-gated ion channel activity involved in regulation of postsynaptic membrane potential	GRIN1;GRIN2A;GRIN2B;GRIA2;GRIA1
regulation of postsynaptic neurotransmitter receptor activity	HOMER1;HOMER3;BEGAIN;DLGAP2;CNIH2;SHISA6
regulation of postsynaptic membrane neurotransmitter receptor levels	DLG3;CACNG8;DLG2;DLG4;IQSEC2;KALRN;SHISA6
trans-synaptic signaling	DAGLA;PLCB1;IQSEC2;HNRNPK;SYNGAP1;SHANK1;DLGAP1 ;DLGAP2;PPP3CA;PTK2B
chemical synaptic transmission	IQSEC2;HNRNPK;SYNGAP1;SHANK1;DLGAP1;DLGAP2 ;PLCB1;PPP3CA;PTK2B
modulation of chemical synaptic transmission	IQSEC2;HNRNPK;SYNGAP1;SHANK1;DLGAP1;DLGAP2
synapse organization	SPTBN2;WASL;HNRNPK;SHANK2;CTTN;WASF1;ITPKA;SPTB; KALRN;RIMS1;PCLO;RIMBP2;BSN;ERC2;ACTN1;SHANK1 ;DLGAP1;DLG4;DLG3;DLG2;LRRC4B;ADD2;NLGN3;PTK2B ;EPHA4;SYNGAP1;ABI1;ROCK2
modification of postsynaptic actin cytoskeleton	WASF1;CTTN;ITPKA;SPTB;KALRN
structural constituent of synapse	RIMS1;PCLO;RIMBP2;BSN;ERC2;SPTBN2;ACTN1;SHANK1 ;DLGAP1;SHANK2;DLG4;DLG3;DLG2
structural constituent of active zone	RIMS1;PCLO;RIMBP2;BSN;ERC2
structural constituent of postsynapse	SPTBN2;ACTN1;SHANK1;DLGAP1;SHANK2;DLG4;DLG3;DLG2
structural constituent of postsynaptic density	SHANK1;DLGAP1;SHANK2;DLG4;DLG3;DLG2
postsynaptic specialization assembly	NLGN3;SPTBN2;LRRC4B;PTK2B

Dataset S1 (separate file): Full mass-spectrometry data-table, including statistics**.**

Dataset S2 (separate file): AL overshoot proteins, relative fold changes and cellular compartment annotation.

Dataset S3 (separate file): MS-DAP report; Quality control mass-spectrometry.
