# Supplementary material for: Torpor enhances synaptic strength and restores memory performance in a mouse model of Alzheimer’s disease
Source: Sci Rep. 2021 Jul 29;11:15486. doi: 10.1038/s41598-021-94992-x (PMC8322095; doi:10.1038/s41598-021-94992-x)
Supplement: Supplementary file 4 — Supplementary Information 4. [file 41598_2021_94992_MOESM4_ESM.pdf]

MS-DAP: Mass Spectrometry Downstream Analysis Pipeline

version: beta 0.2.5 https://github.com/ftwkoopmans/msdap/

Contents

1 Quality control **2**

1.1 number of peptides and proteins 2

1.2 data completeness 7

1.3 abundance distributions 9

1.4 retention time 12

1.5 variation among replicates 28

1.6 PCA 36

2 Differential abundance analysis **42**

2.1 torpor_late vs arousal_late 42

2.2 torpor_late vs eutherm 47

2.3 arousal_late vs eutherm 52

3 Summary of differential testing **57**

4 log **58**

5 R command history **59**

6 R session info **60**

1 Quality control

The quality control figures in this section enable you to investigate reproducibility and global clustering of samples by visualizing:

- number of peptides/proteins detected in each sample
- dataset completeness
- local effects in HPLC peptide retention time per sample
- reproducibility of peptide quantification among replicates
- PCA of all samples to visualize clustering

The first set of quality control figures describes individual samples, thereafter group-level quality metrics are described and finally sample clustering is used to highlight structure in the entire dataset.

1.1 number of peptides and proteins

These plots show the number of (target) peptides that are 'detected' per sample. For DDA, 'detected' implies the peptide has a MS/MS identification. Peptides quantified through match-between-runs (MBR) are quantified but not detected/identified. In case of DDA, we also show the number of peptides quantified through MBR. For DIA, we refer to a peptide as 'detected' if the confidence score (for identification) is <= 0.01.

Samples in this plot are sorted by their experimental group, and then ordered and by their name within each group. This data is also available in the output table 'samples.xlsx'.

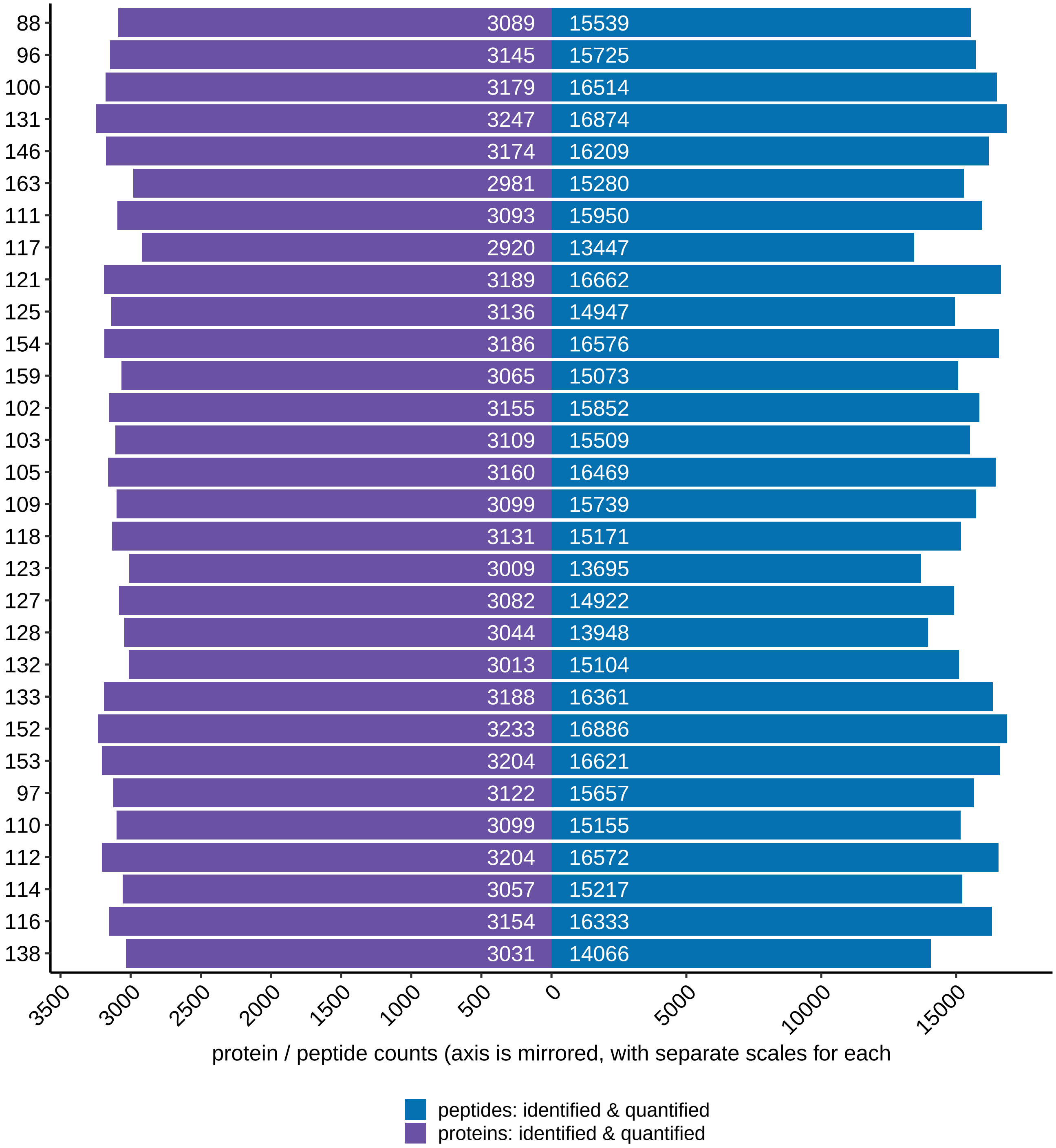
88
96
100
131
146
163
111
117
121
125
154
159
102
103
105
109
118
123
127
128
132
133
152
153
97
110
112
114
116
138
3089 15539
3145 15725
3179 16514
3247 16874
3174 16209
2981 15280
3093 15950
2920 13447
3189 16662
3136 14947
3186 16576
3065 15073
3155 15852
3109 15509
3160 16469
3099 15739
3131 15171
3009 13695
3082 14922
3044 13948
3013 15104
3188 16361
3233 16886
3204 16621
3122 15657
3099 15155
3204 16572
3057 15217
3154 16333
3031 14066
3500
3000
2500
2000
1500
1000
500
0
5000
10000
15000
protein / peptide counts (axis is mirrored, with separate scales for each
peptides: identified & quantified
proteins: identified & quantified

1.1.1 color-coding sample metadata

The number of detected peptides in a sample, as compared to other samples within a dataset, can be used as a measure for sample quality. Color-coding individual samples for metadata that you provided as input (e.g. experiment batch, sample handling order, gel lanes, etc.) allows visual inspection as to whether these relate to the rate of successful peptide detection.

The figure below aims to provide an overview of all sample metadata at a first glance. The y-axis shows all sample metadata. On each row all samples in the dataset are shown as a dot, each color-coded by the respective property shown on the y-axis (with minor vertical jitter for visual clarity). If there is a major effect of any experimental condition on the number of detected peptides, this is easily spotted as all outlier samples (extremes in number peptide detection, x-axis) will have the same color. The following figures expand this overview into a detailed figure for each sample property, those visualization are designed to further dive into each experimental condition and provide respective color legends.

Note that the visualization of sample metadata in this report depends on user-provided input; each column in the metadata input table (besides sample names) that contains more than 1 unique value is automatically used as a factor for color-coding all figures in this section. So the data used for color-coding is also available in the output table 'samples.xlsx'.

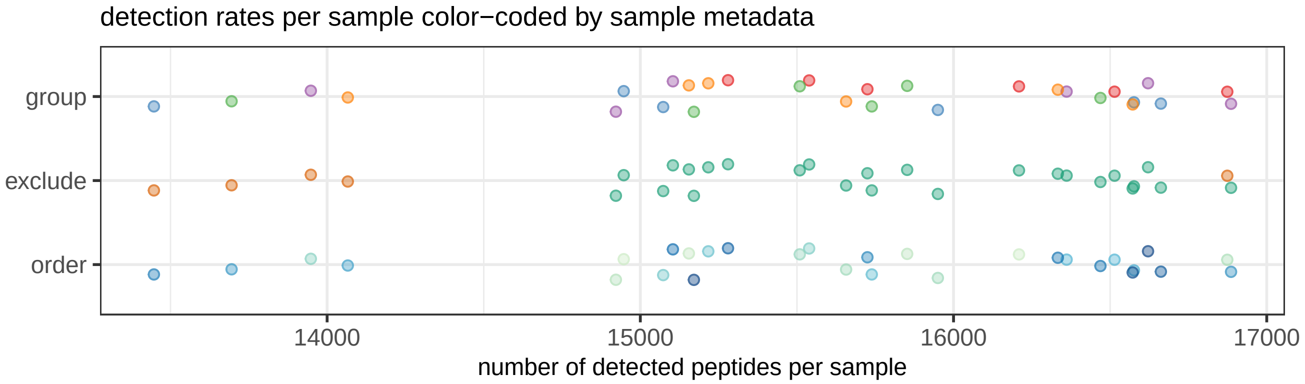

color-coding sample metadata, expanded

To further detail each sample property, each row in the above figure is now split into separate plots. Thus, a figure is generated for each experimental condition from the user-provided metadata (column in the samples table, it's name shown in the plot title).

The y-axis shows all unique variables, the x-axis the number of detected peptides and each dot is a sample. The vertical line indicates the median value for each row. Colors are consistent with the above plot.

For example: the first plot shows color-coding by the 'group' property, so each row represents a sample group. If samples in a particular group systematically yield fewer peptides than another group, a clear pattern will be visible.

Note that the *exclude* metadata is a user-provided indication of 'outlier samples' in the dataset that are excluded from downstream statistical analysis.

sample metadata used for color−coding: group

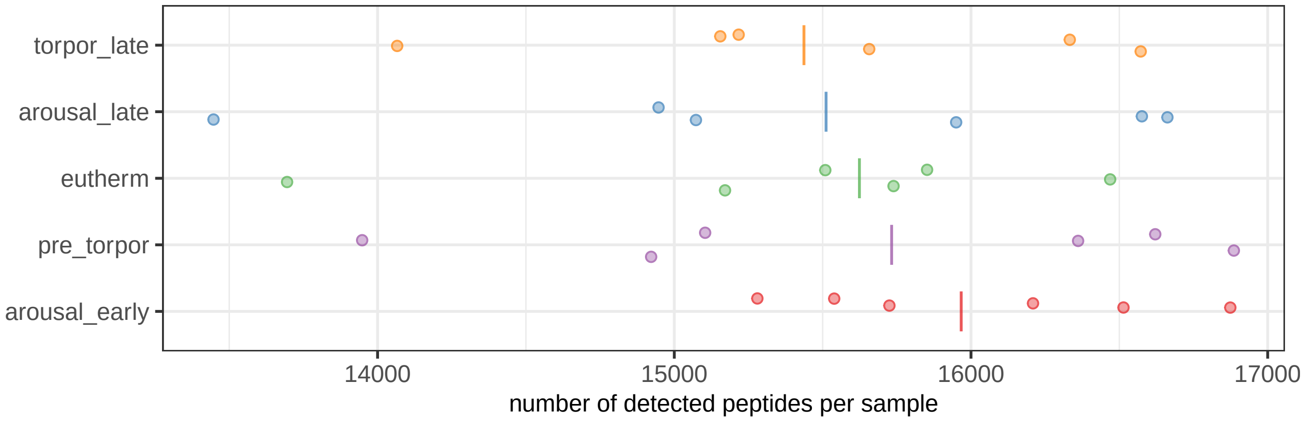

sample metadata used for color−coding: exclude

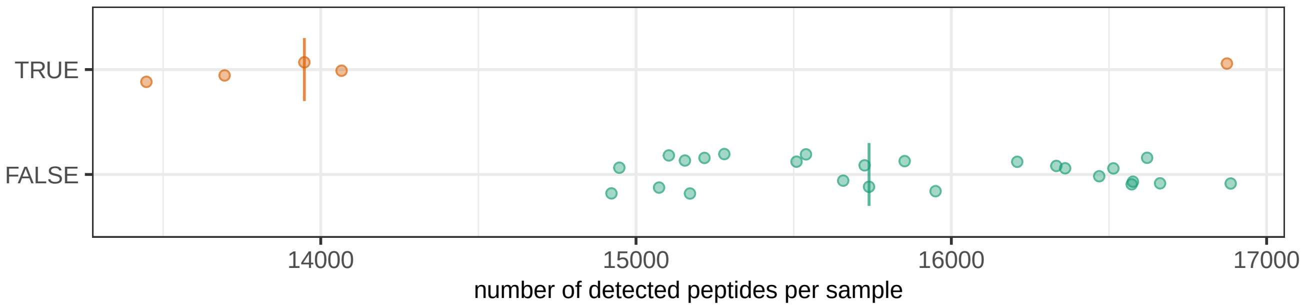

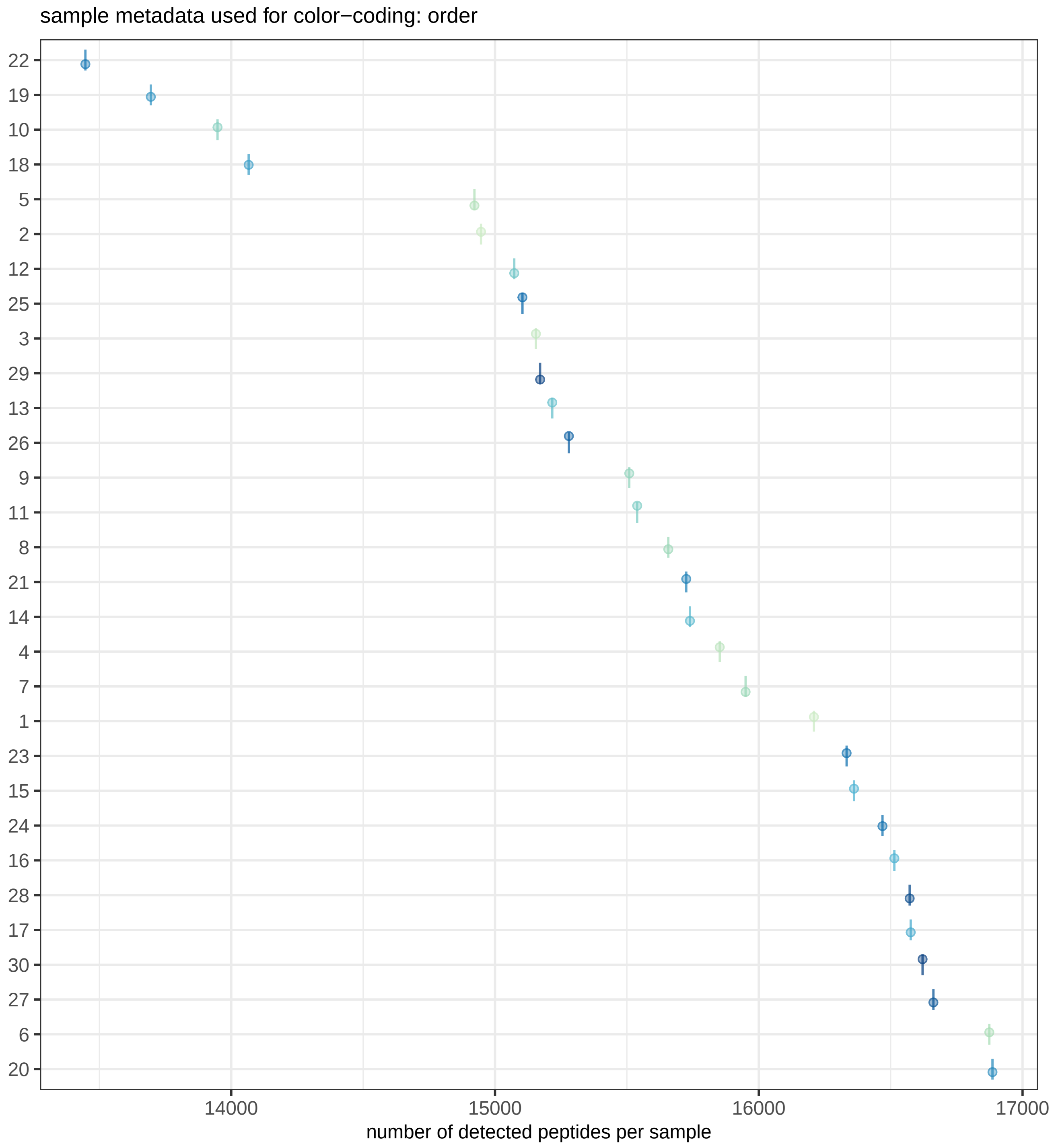
sample metadata used for color−coding: order
22
19
10
18
5
2
12
25
3
29
13
26
9
11
8
21
14
4
7
1
23
15
24
16
28
17
30
27
6
20
14000
15000
16000
17000
number of detected peptides per sample

1.2 data completeness

To visualize how many peptides are consistently identified in multiple samples, the first figure summarizes how common missing values are in the entire dataset. Optimally, most peptides are identified in 100% of samples and this curve slowly falls of. The following figure shows for each sample whether its peptides are also present in other samples in the dataset or whether these are unique to a (minor) subset of samples. You can use this mark of experimental consistency to compare datasets generated by similar protocols and mass-spec acquisition.

1.2.1 cumulative distribution

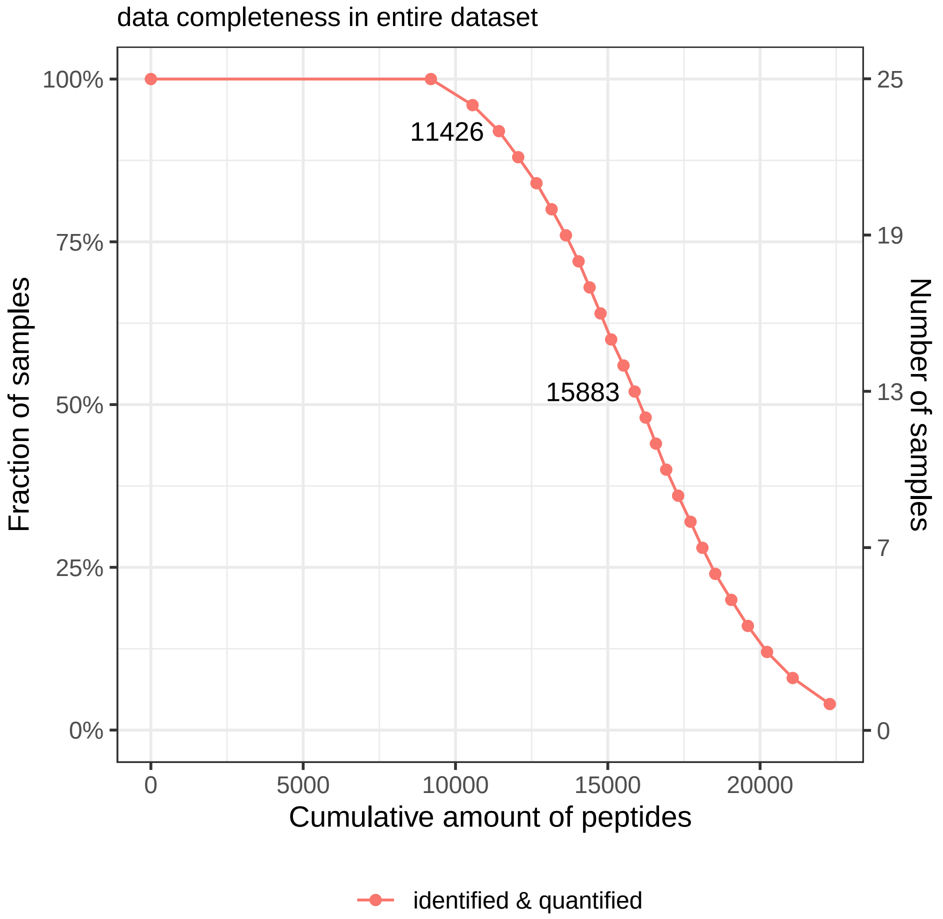

Samples flagged as 'exclude' (by user) are not taken into account in this figure. Exact values are shown for data points matching 90% and 50% of samples to convenience comparison between analyses (e.g. before/after configuring 'exclude' samples, or comparing between experiments of similar protocol).

1.2.2 peptide detection frequency

Each identified peptide in a sample is classified and color-coded by the number of other samples where the same peptide is present. Visualization of the amount of peptides that overlap with other samples in the dataset, from peptides identified in most samples (red) to one-hit-wonders (blue), helps identify uncommon samples (more blue/green than other samples).

Optimally, the majority of peptides in each sample are red~orange with relatively few uniquely identified peptides (blue~green). Samples are sorted by the total amount of detected peptides.

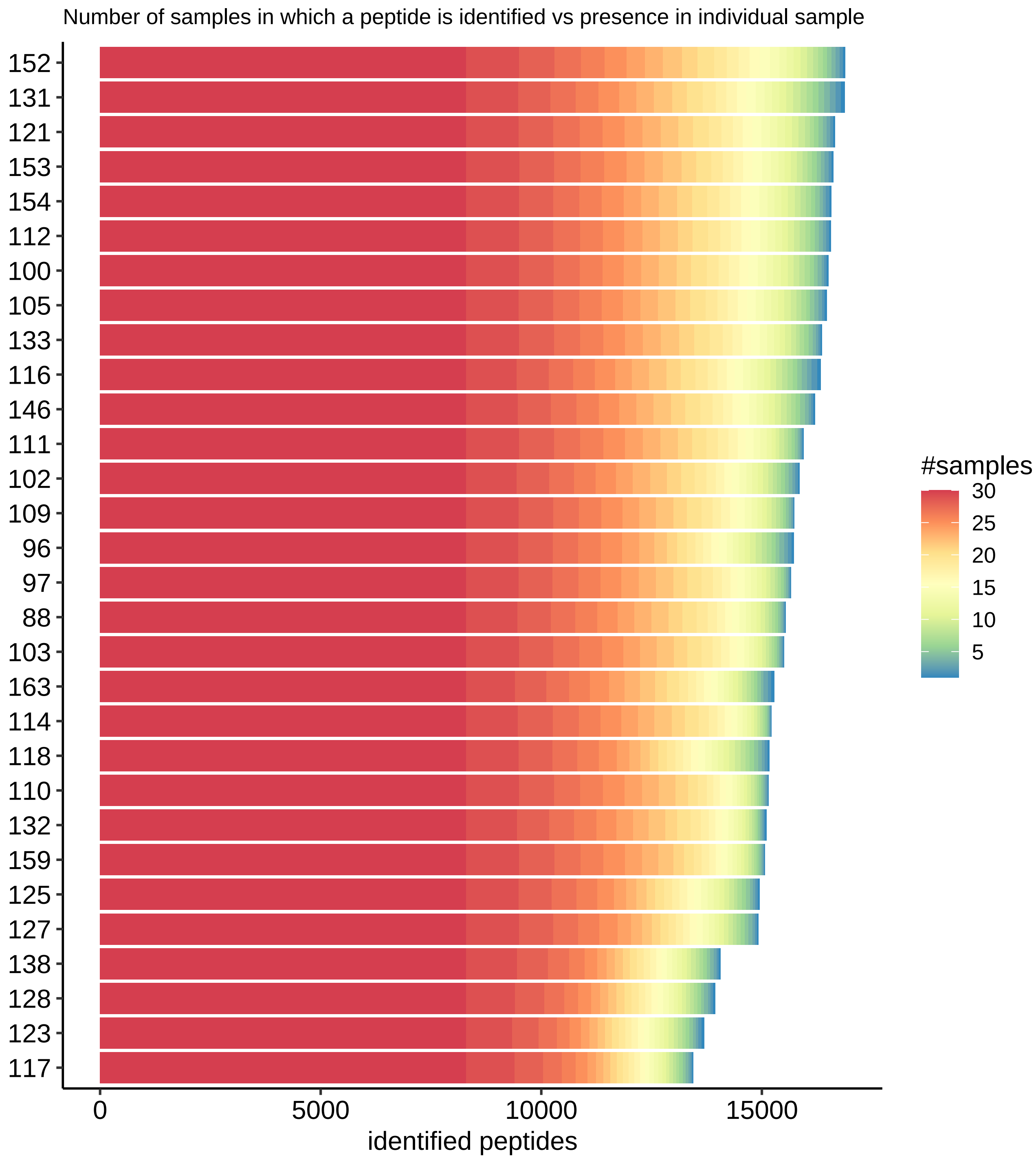

Number of samples in which a peptide is identified vs presence in individual sample
152
131
121
153
154
112
100
105
133
116
146
111
102
109
96
97
88
103
163
114
118
110
132
159
125
127
138
128
123
117
0
5000
10000
15000
identified peptides
#samples
30
25
20
15
10
5

1.3 abundance distributions

The figures in this subsection are used to identify unexpected mass-spec sensitivity or sample loading differences. Peptide data is shown as provided in input files, so peptide filtering nor intensity normalization has been applied yet (for proper QC, make sure the software that generated the input data did not apply normalization prior). If the dataset is DDA, match-between-runs (MBR) peptides are included in these distributions whereas for DIA only 'detected' peptides (based on confidence score threshold) are included.

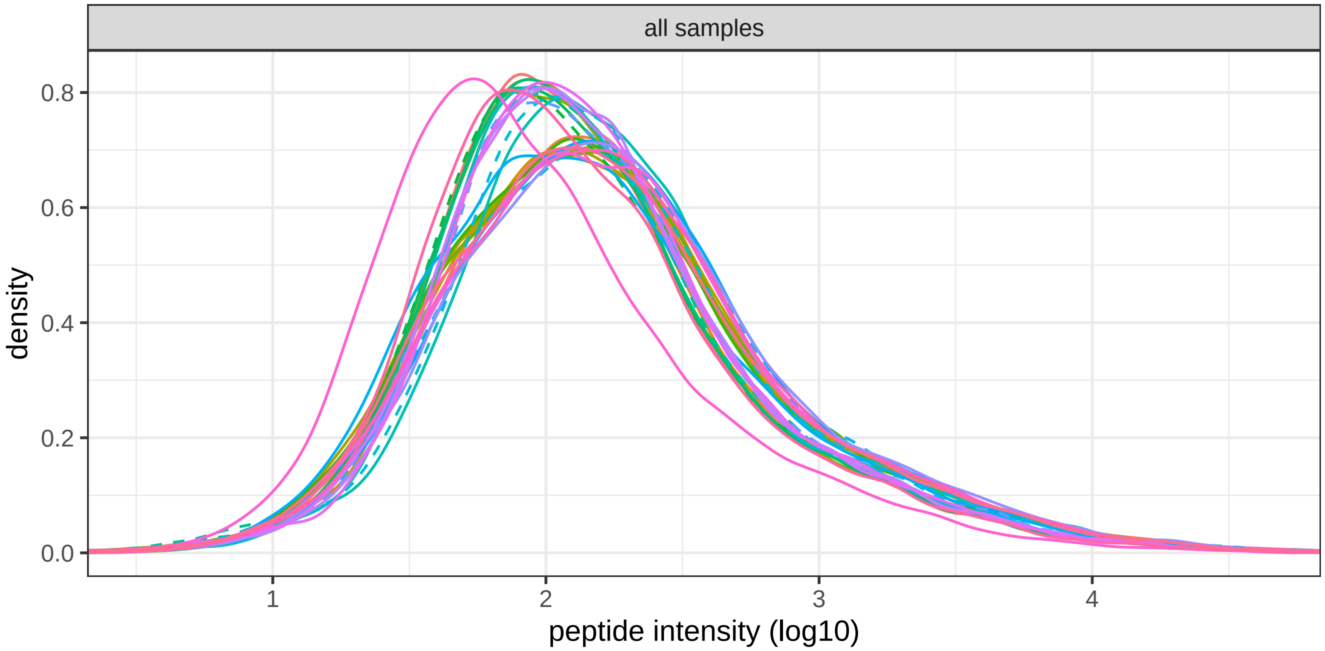

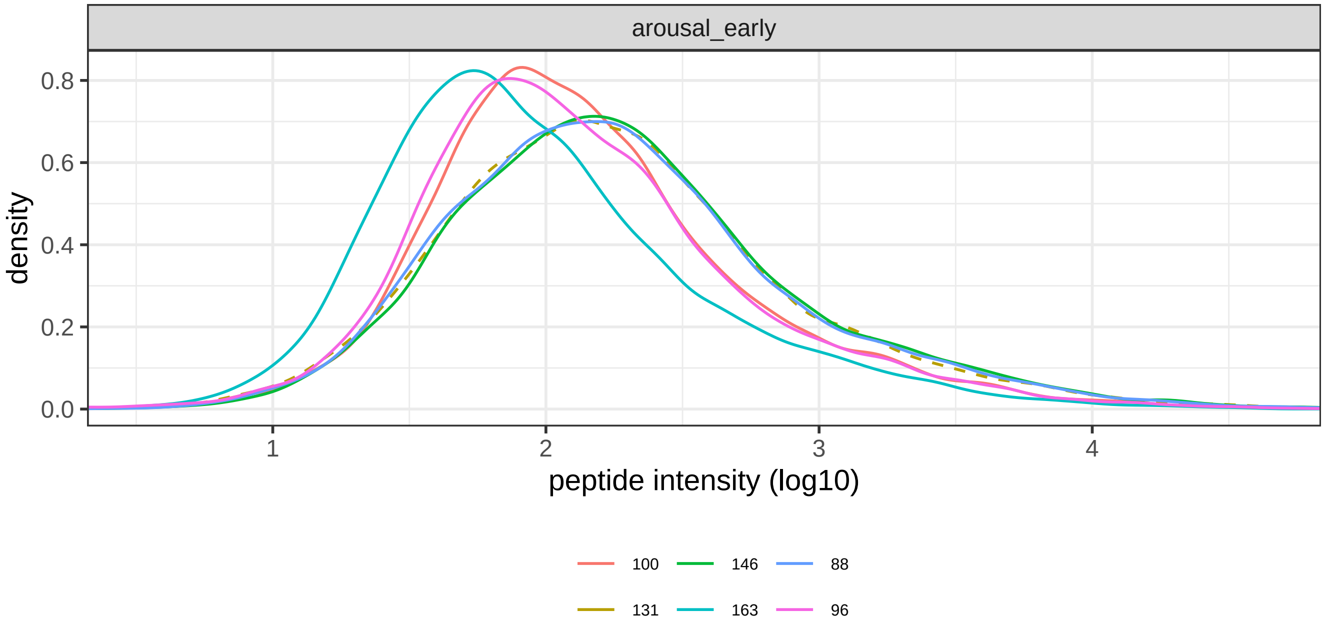

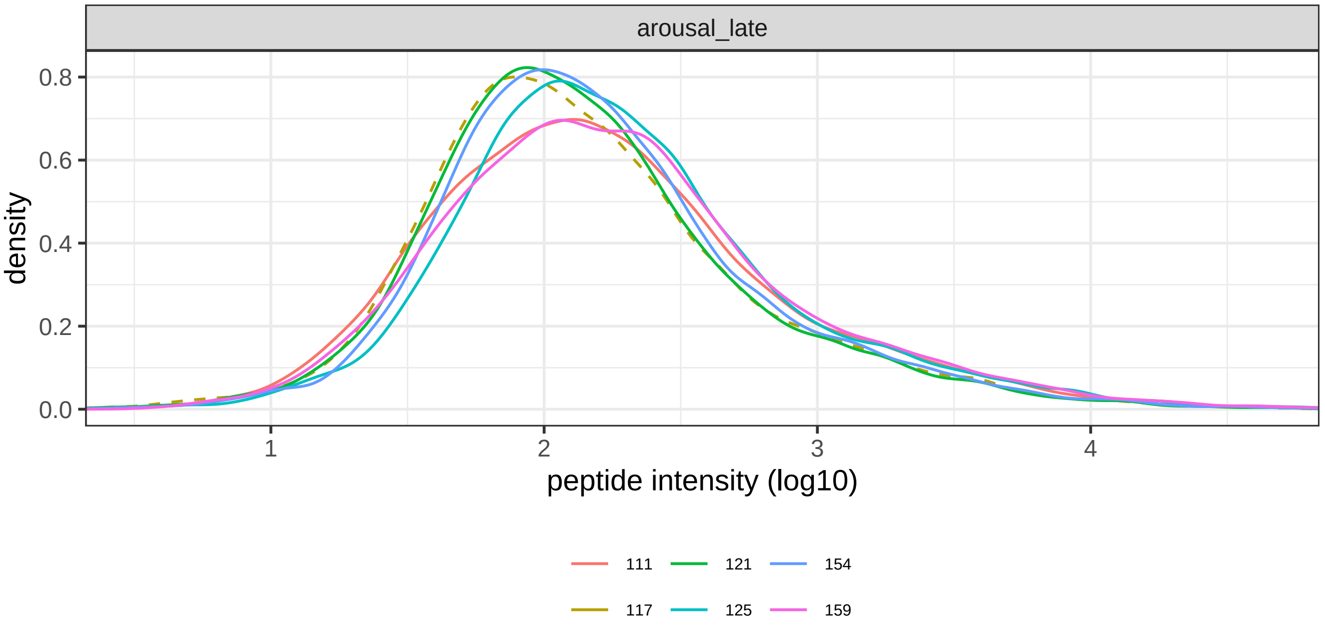
arousal_late
density
0.8
0.6
0.4
0.2
0.0
1
2
3
4
peptide intensity (log10)
111
121
154
117
125
159

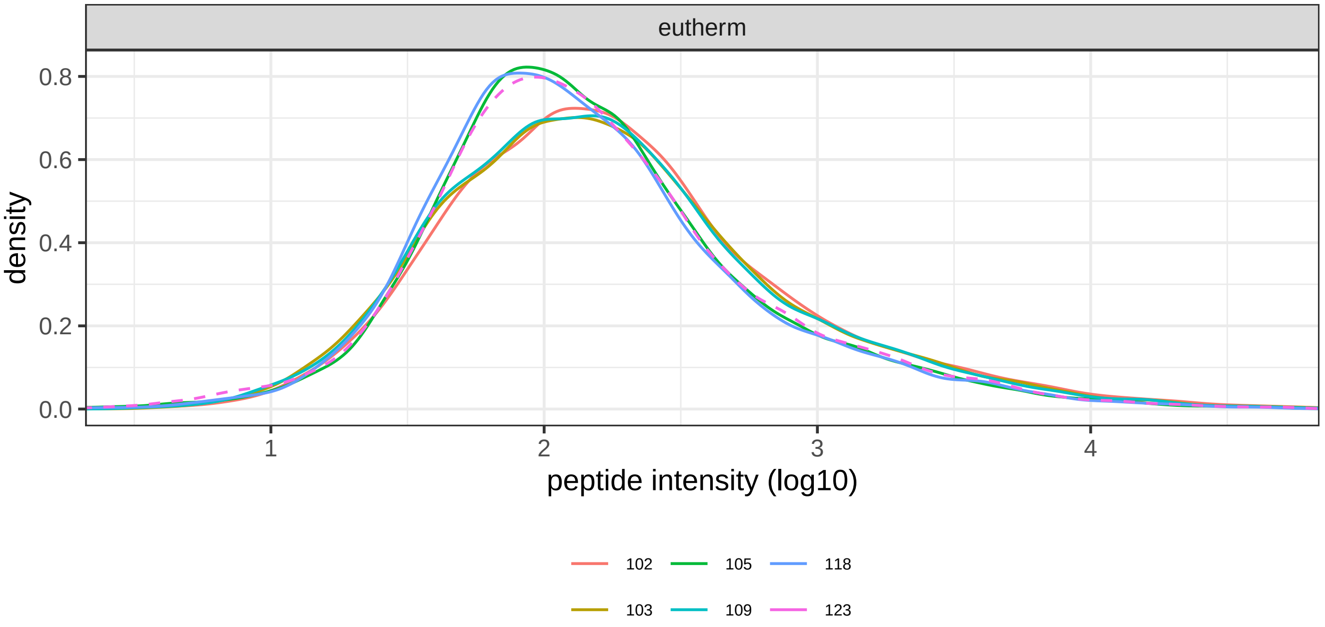
eutherm
density
0.8
0.6
0.4
0.2
0.0
1
2
3
4
peptide intensity (log10)
102
105
118
103
109
123

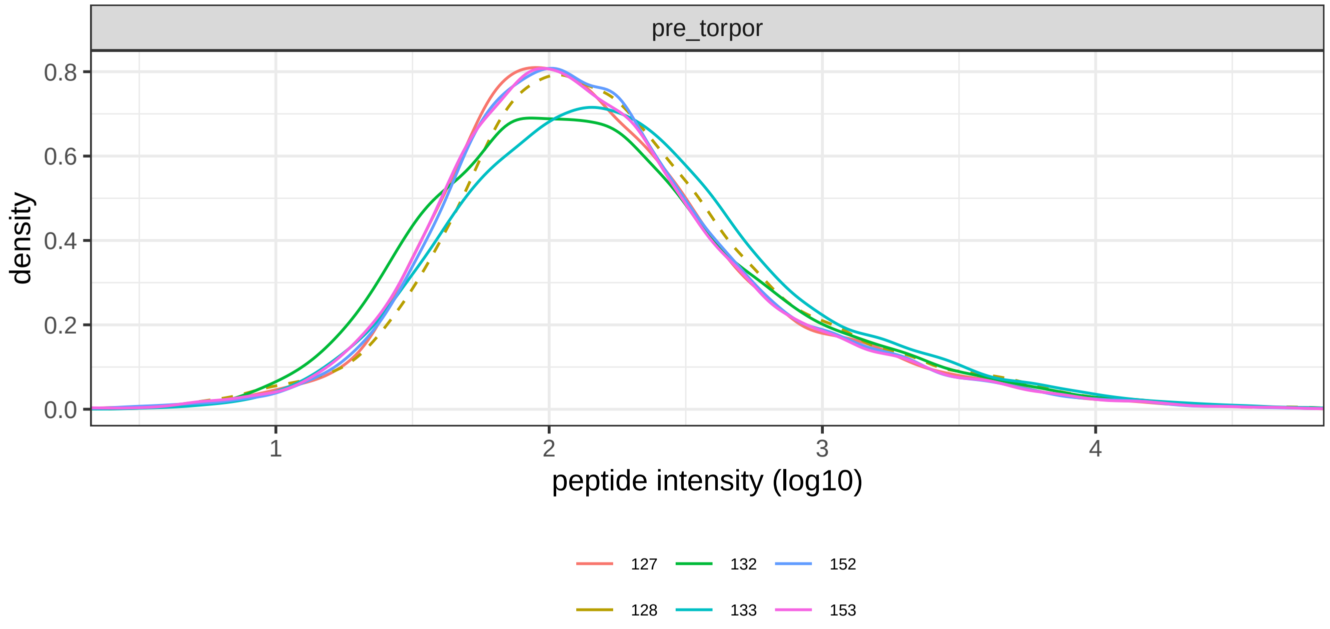
pre_torpor
0.8
0.6
0.4
0.2
0.0
density
1
2
3
4
peptide intensity (log10)
127
132
152
128
133
153

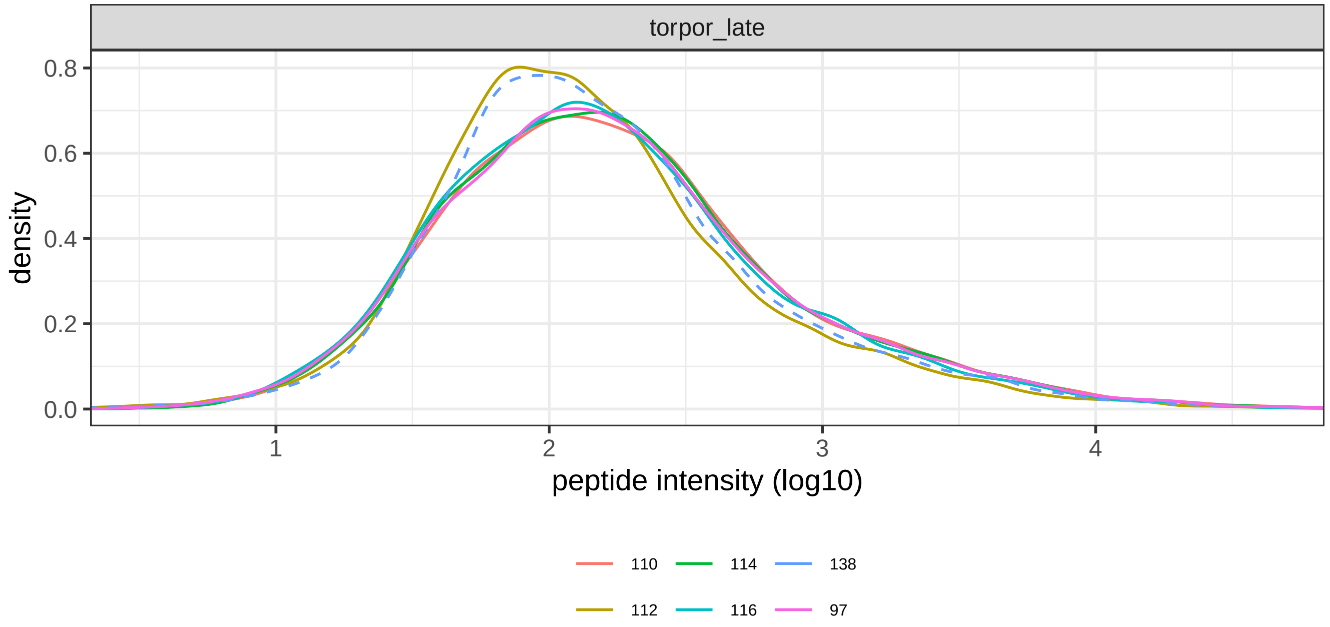
torpor_late
0.8
0.6
0.4
0.2
0.0
density
1
2
3
4
peptide intensity (log10)
110
114
138
112
116
97

1.4 retention time

The figures in this section allow you to identify potential problems during HPLC elution, such as a temporarily blocking column, failing ionization spray or decreasing sensitivity over time. For each sample, all peptides that are also observed in a replicate (such that there is a point of reference available) are visualized.

1.4.1 retention time distributions

The density of the number of peptides eluting at each point in time. The figure below presents an overview of all samples that allows for the identification of outlier samples that follow distinct elution patterns. The following section shows details for each sample. Samples marked as 'exclude' in the provided sample metadata table are visualized as dashed lines.

progress: RT plots: preparing data took 17 seconds progress: RT plots: creating plots took 3 seconds

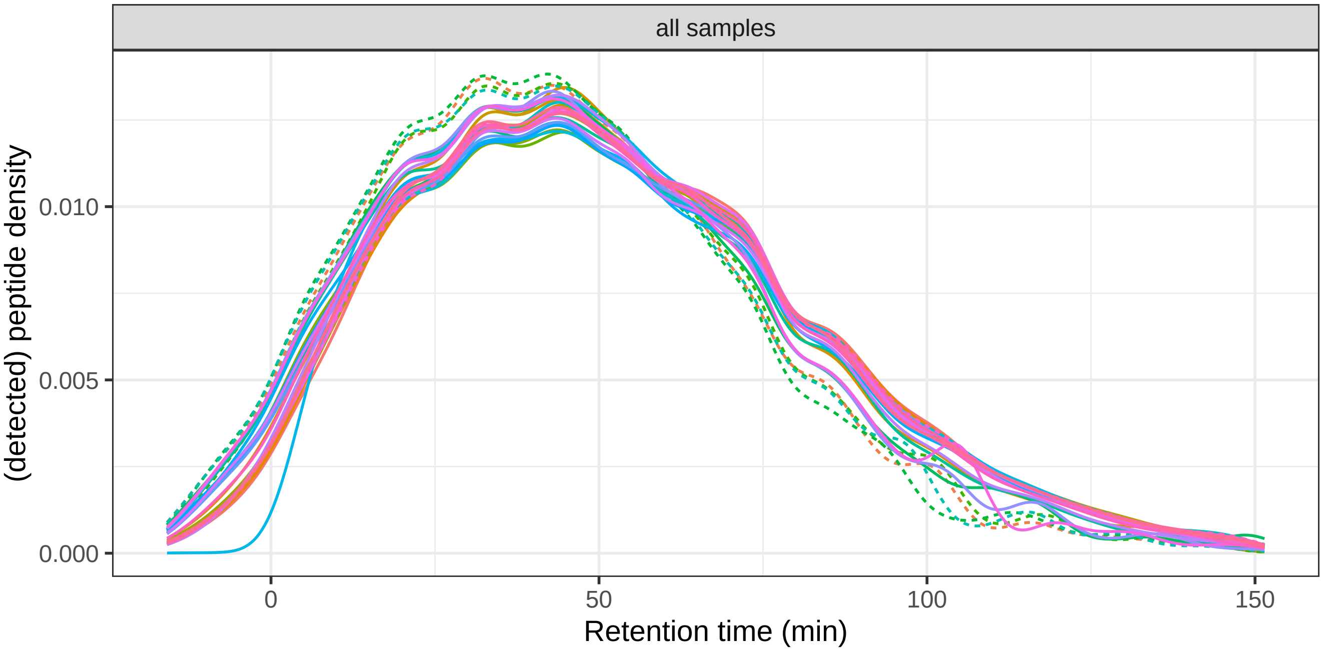

1.4.2 retention time local effects

To investigate how each measurement differs from others, we visualize each sample as a 3 panel figure. First, the data is binned across the retention time dimension (x-axis). If a samples was marked as 'exclude' in the provided sample metadata, this is indicated in the plot title.

The top panel shows the number of peptides in the input data, e.g. as recognized by the software that generated input for this pipeline, over time (black line). For reference, the grey line shows the median amount over all samples (note; if this is the exact same in all samples, the grey line may not be visible as it falls behind the black line).

The middle panel indicates whether peptide retention times deviate from their median over all samples (blue line). The grey area depicts the 5% and 95% quantiles, respectively. The line width corresponds to the number of peptides eluting at that time (data from first panel). Analogously, the bottom panel shows the deviation in peptide abundance as compared to the median over all samples (red line).

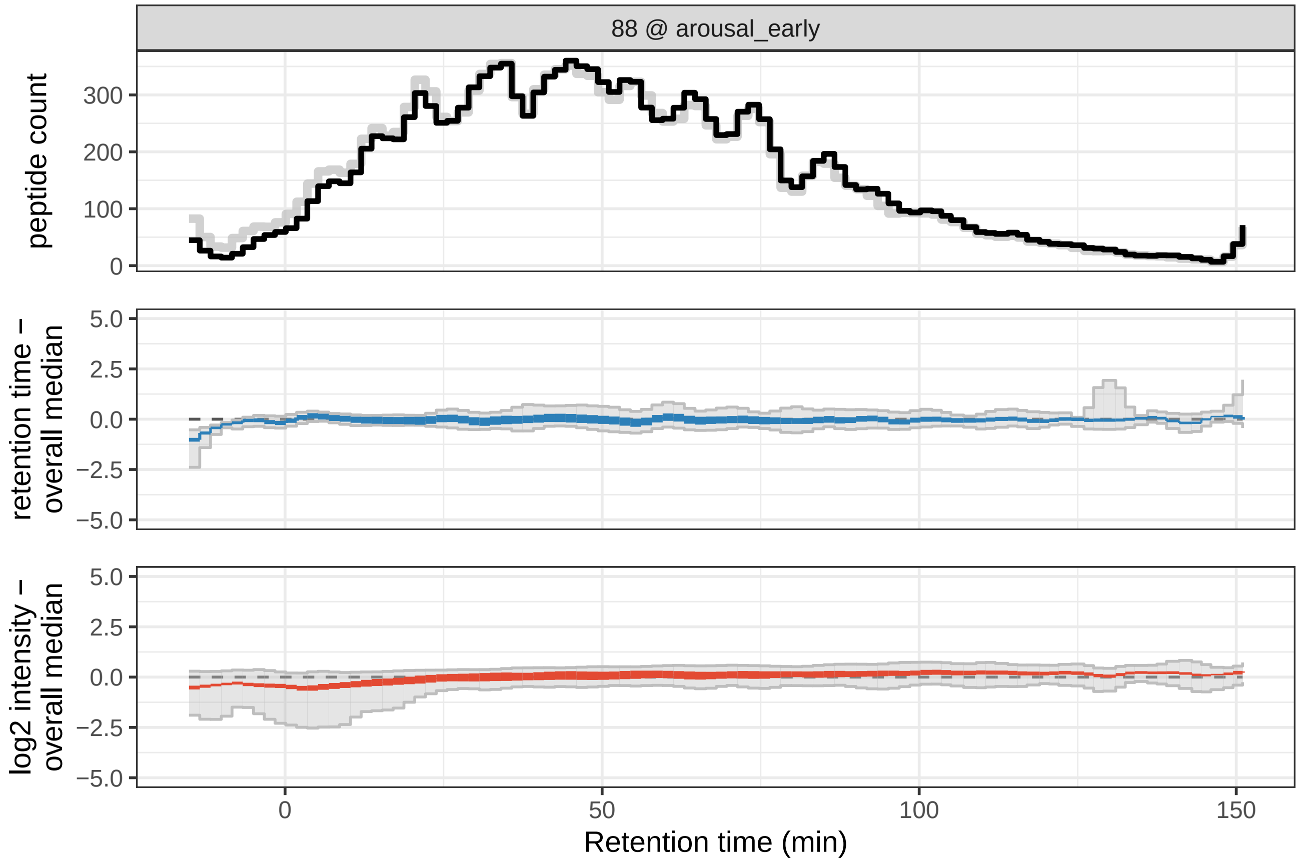
88 @ arousal_early
peptide count
retention time – overall median
log2 intensity – overall median
Retention time (min)

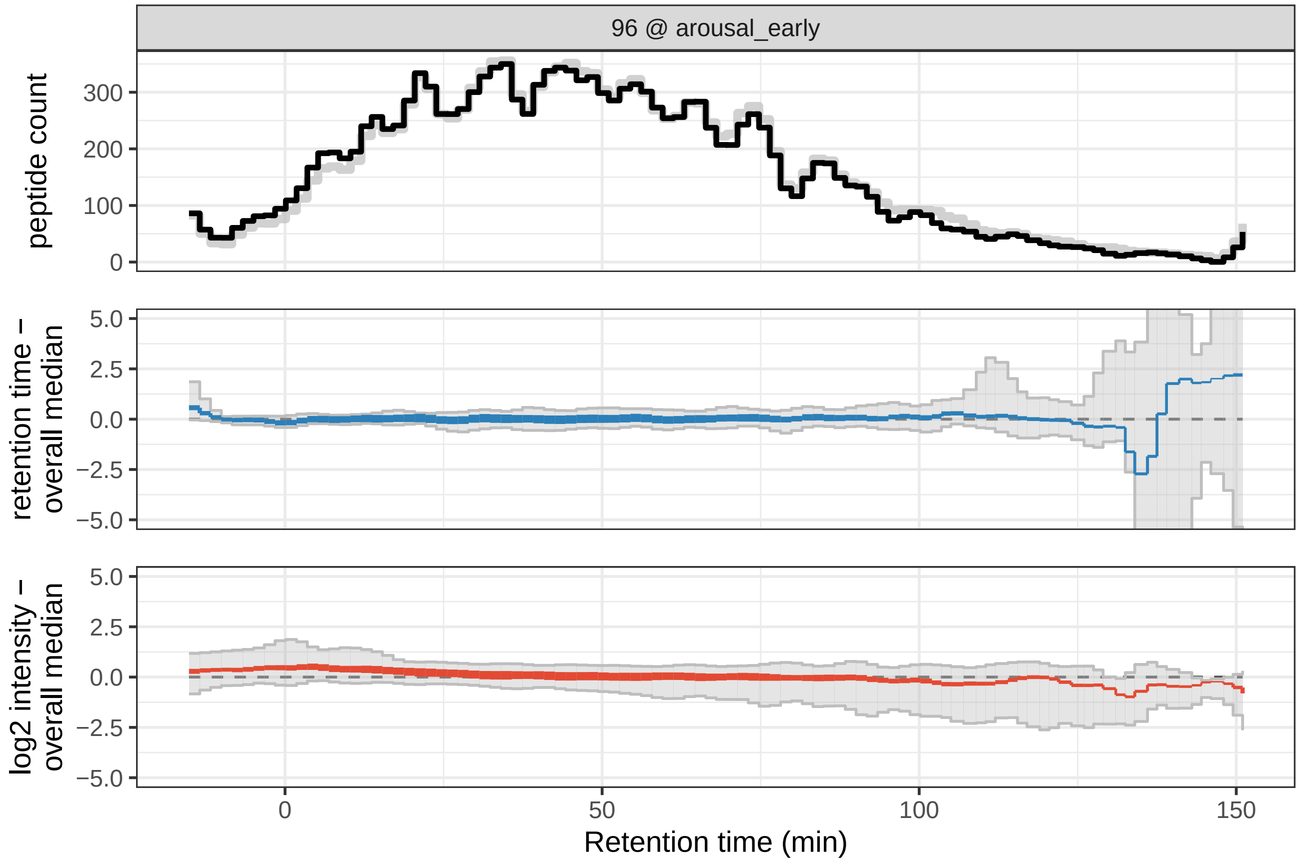
96 @ arousal_early
peptide count
retention time – overall median
log2 intensity – overall median
Retention time (min)

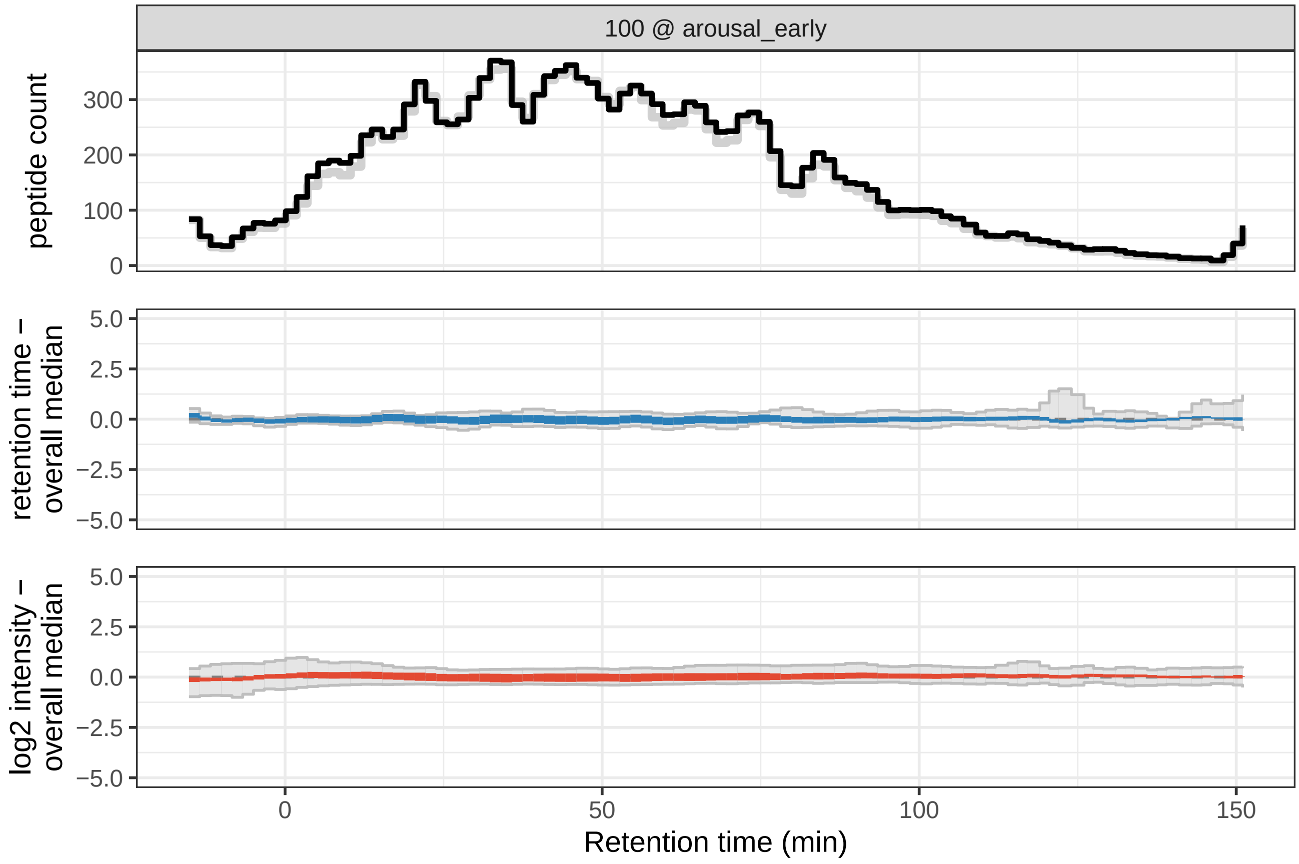
100 @ arousal_early
peptide count
300
200
100
0
retention time –
overall median
5.0
2.5
0.0
−2.5
−5.0
log2 intensity –
overall median
5.0
2.5
0.0
−2.5
−5.0
0
50
100
150
Retention time (min)

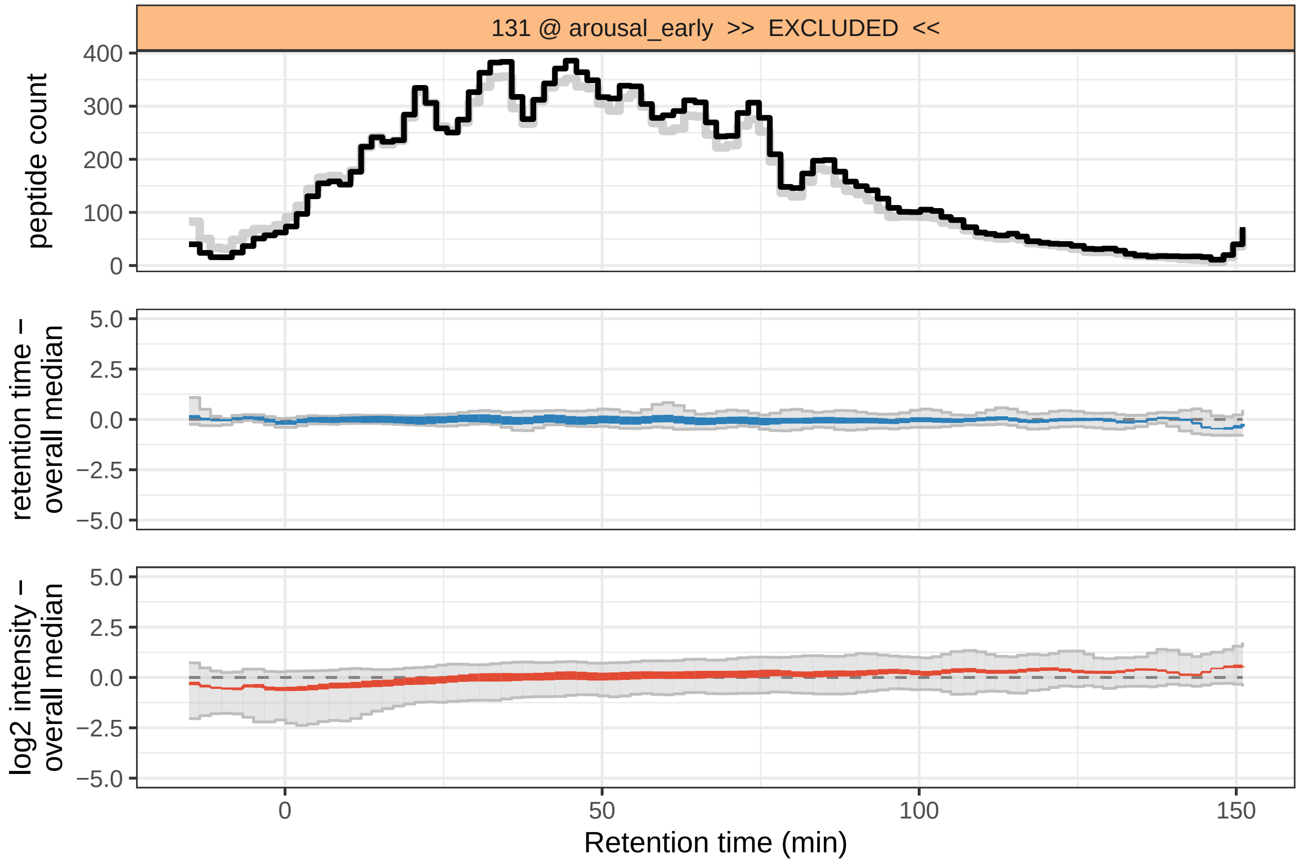
131 @ arousal_early >> EXCLUDED <<
peptide count
400
300
200
100
0
retention time –
overall median
5.0
2.5
0.0
−2.5
−5.0
log2 intensity –
overall median
5.0
2.5
0.0
−2.5
−5.0
0
50
100
150
Retention time (min)

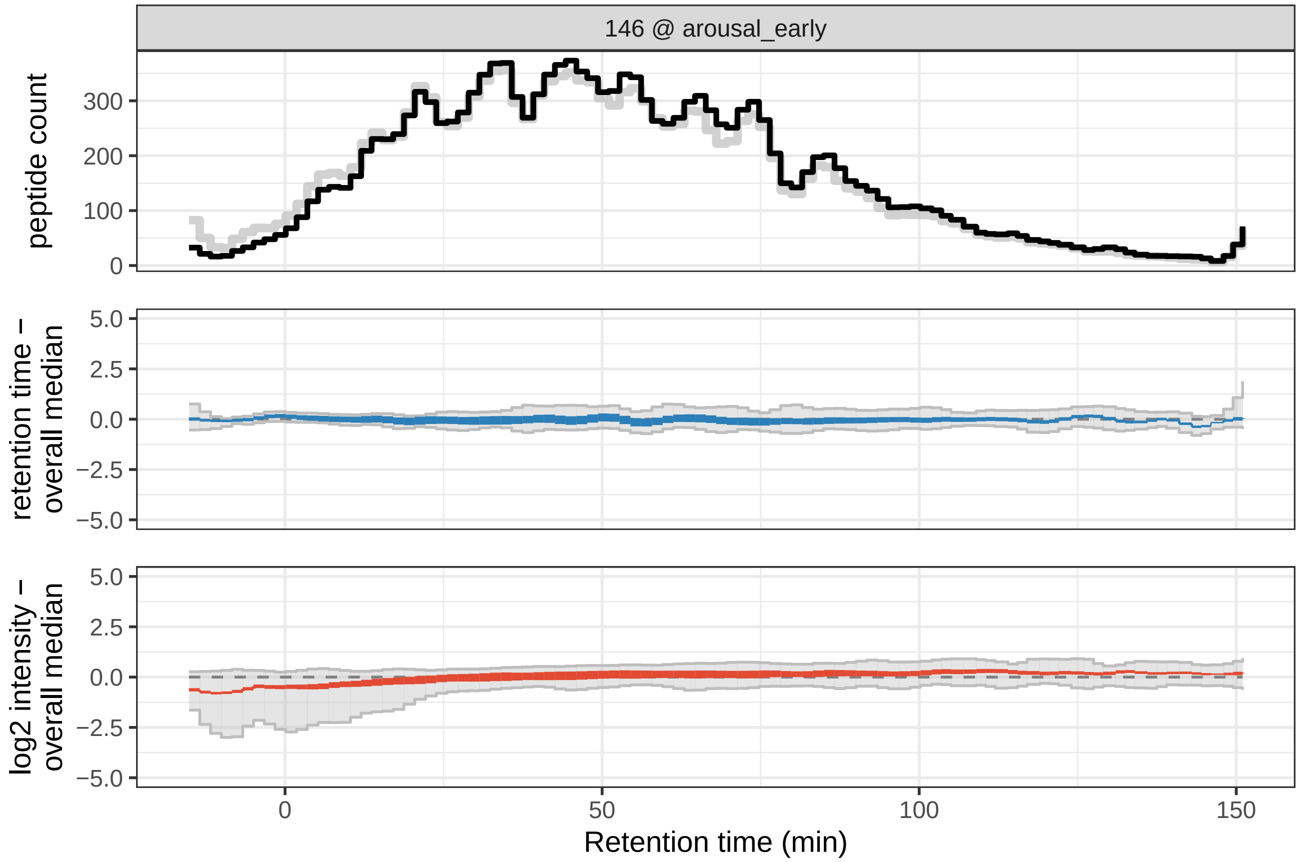
146 @ arousal_early
peptide count
300
200
100
0
retention time –
overall median
5.0
2.5
0.0
−2.5
−5.0
log2 intensity –
overall median
5.0
2.5
0.0
−2.5
−5.0
0
50
100
150
Retention time (min)

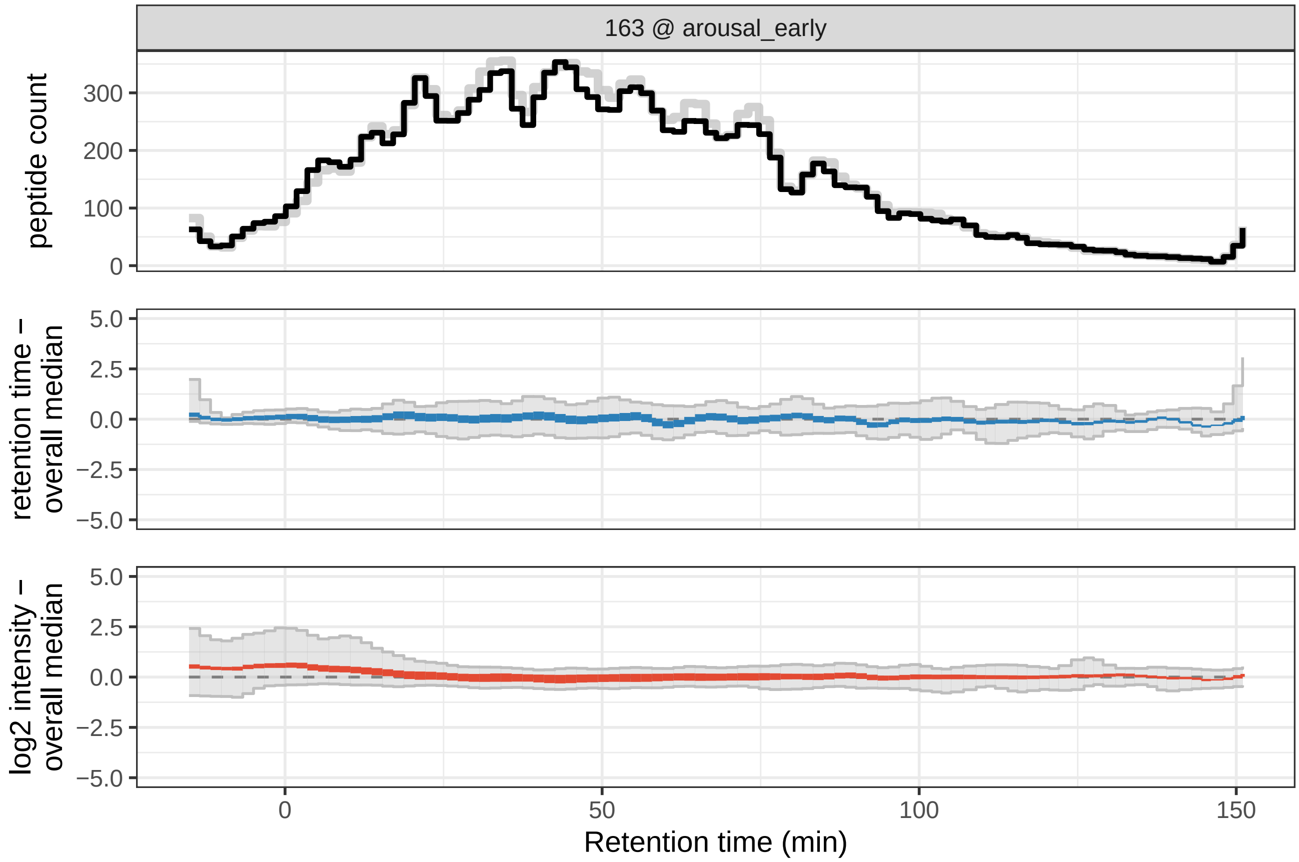
163 @ arousal_early
peptide count
300
200
100
0
retention time –
overall median
5.0
2.5
0.0
−2.5
−5.0
log2 intensity –
overall median
5.0
2.5
0.0
−2.5
−5.0
0
50
100
150
Retention time (min)

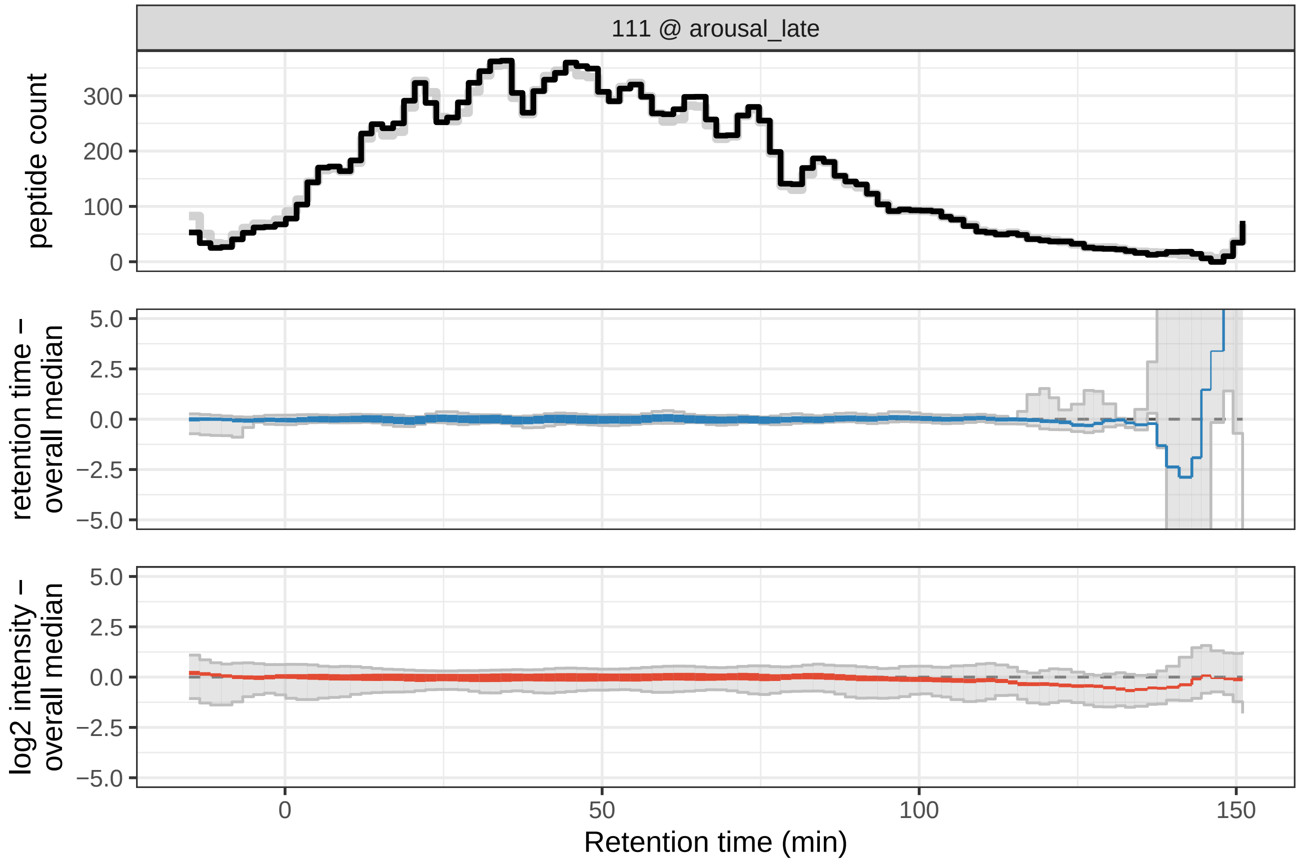
111 @ arousal_late
peptide count
retention time – overall median
log2 intensity – overall median
Retention time (min)

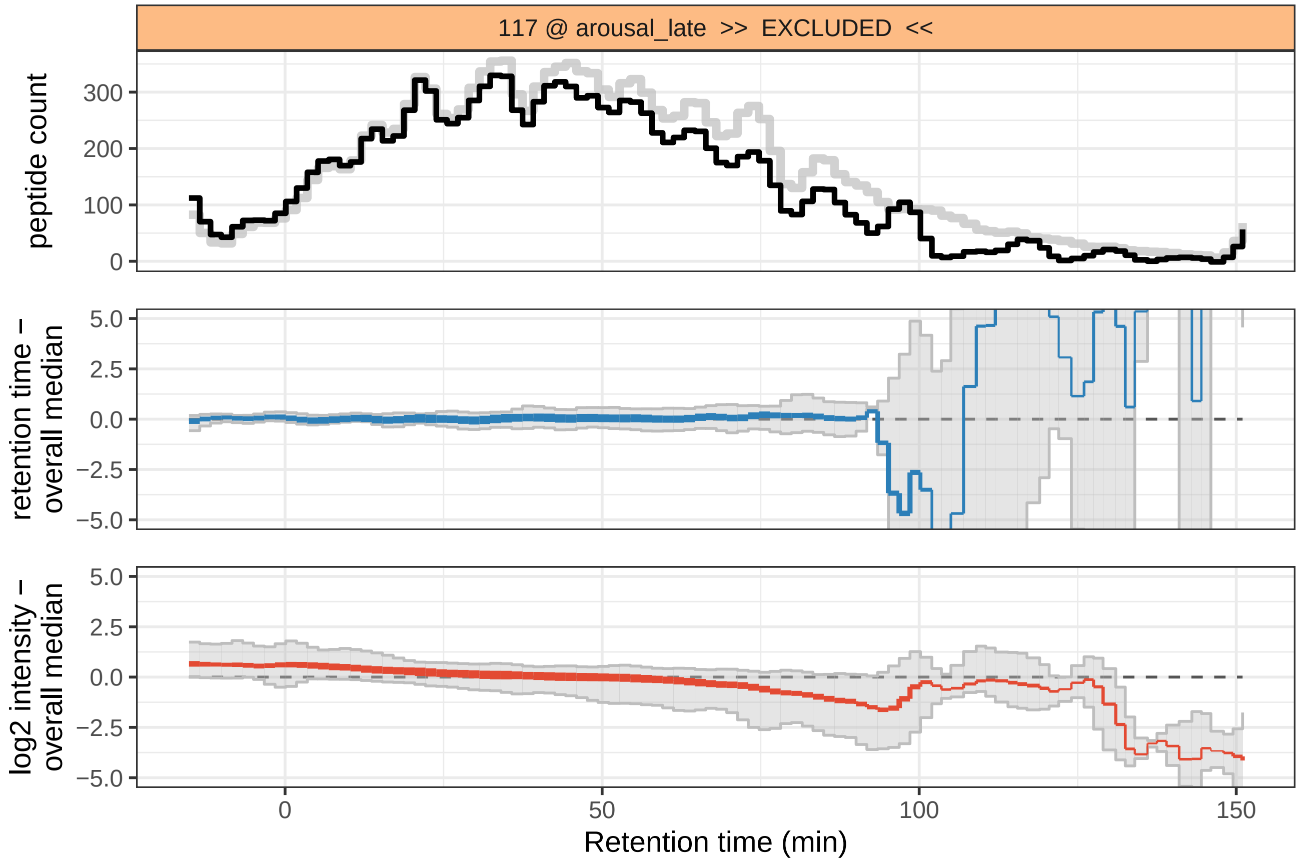
117 @ arousal_late >> EXCLUDED <<
peptide count
retention time – overall median
log2 intensity – overall median
Retention time (min)

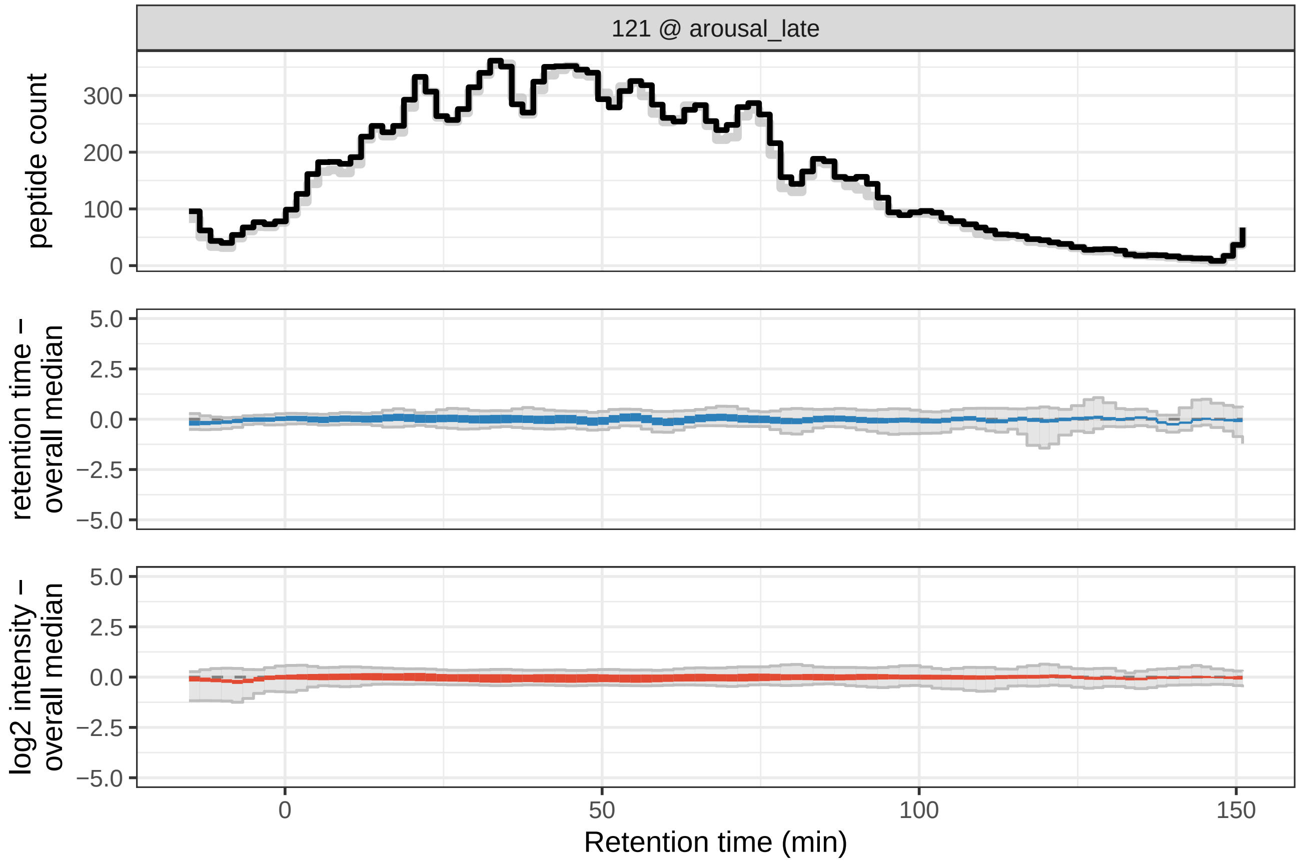
121 @ arousal_late
peptide count
retention time – overall median
log2 intensity – overall median
Retention time (min)

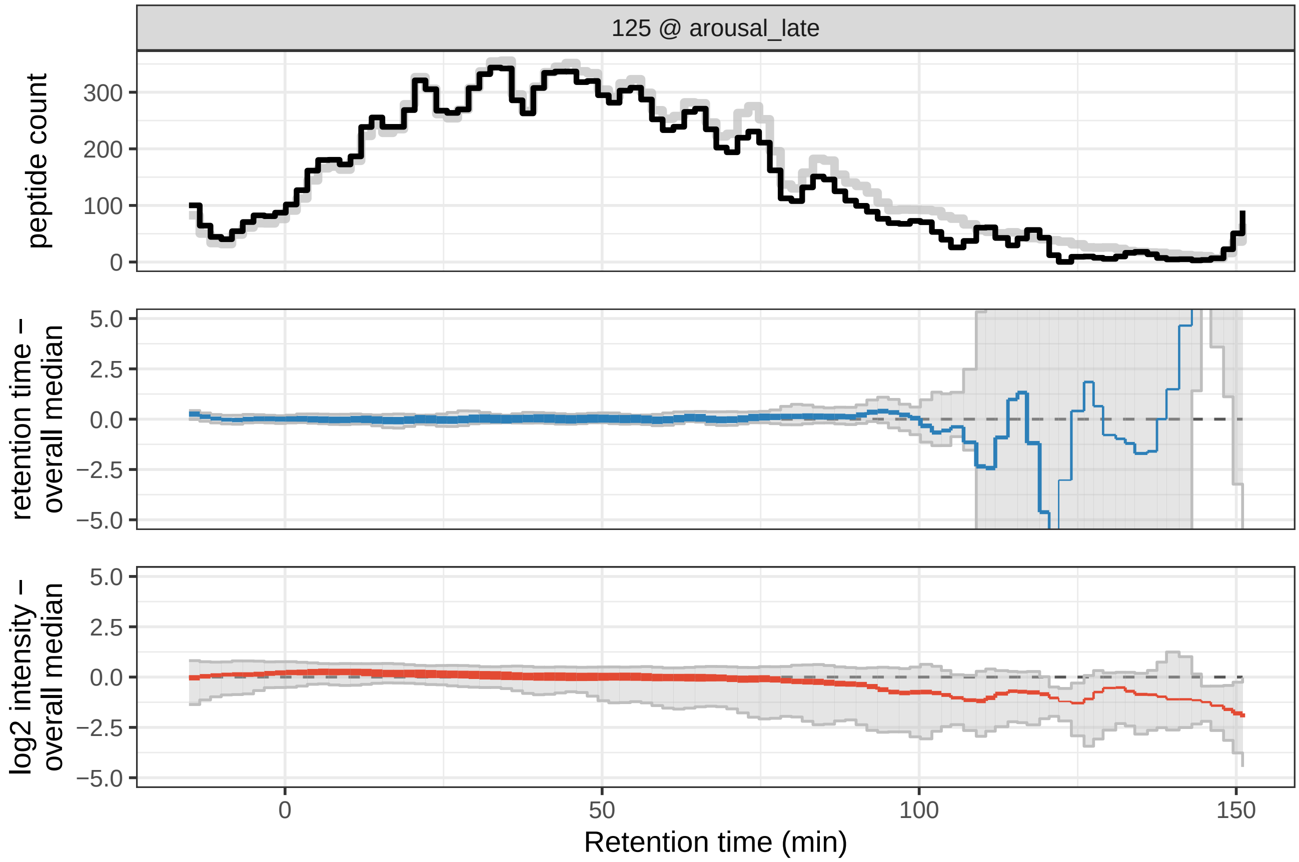
125 @ arousal_late
peptide count
retention time – overall median
log2 intensity – overall median
Retention time (min)

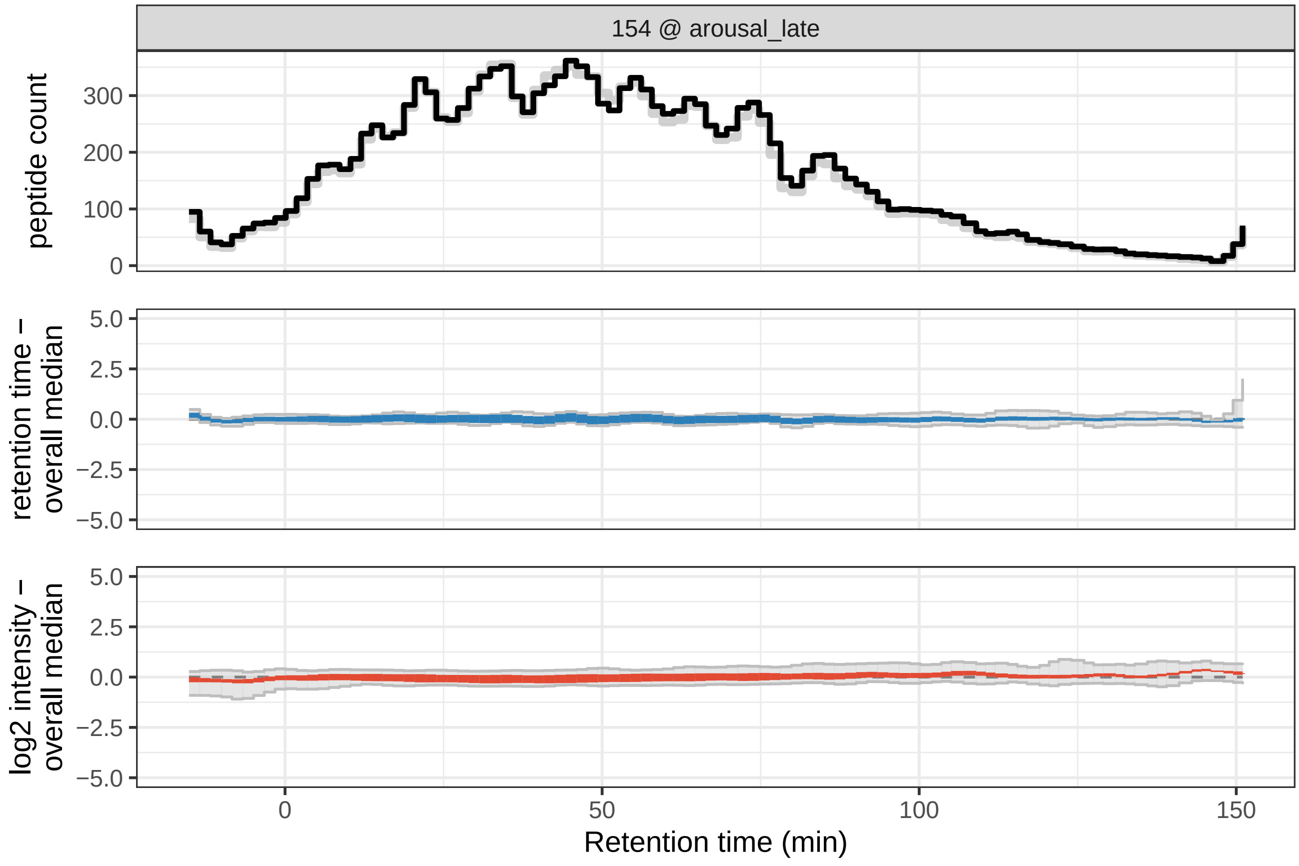
154 @ arousal_late
peptide count
300
200
100
0
retention time –
overall median
5.0
2.5
0.0
−2.5
−5.0
log2 intensity –
overall median
5.0
2.5
0.0
−2.5
−5.0
0
50
100
150
Retention time (min)

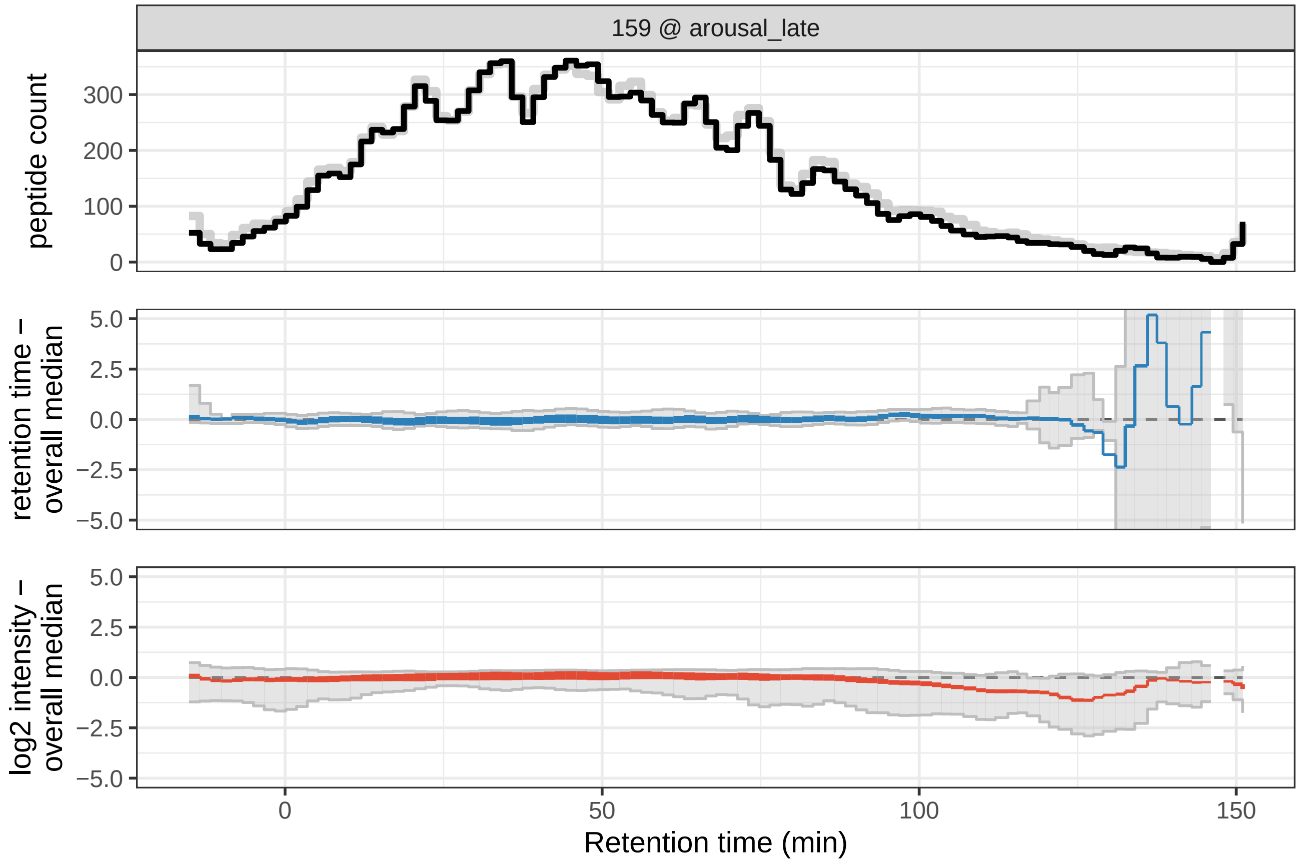
159 @ arousal_late
peptide count
300
200
100
0
retention time –
overall median
5.0
2.5
0.0
−2.5
−5.0
log2 intensity –
overall median
5.0
2.5
0.0
−2.5
−5.0
0
50
100
150
Retention time (min)

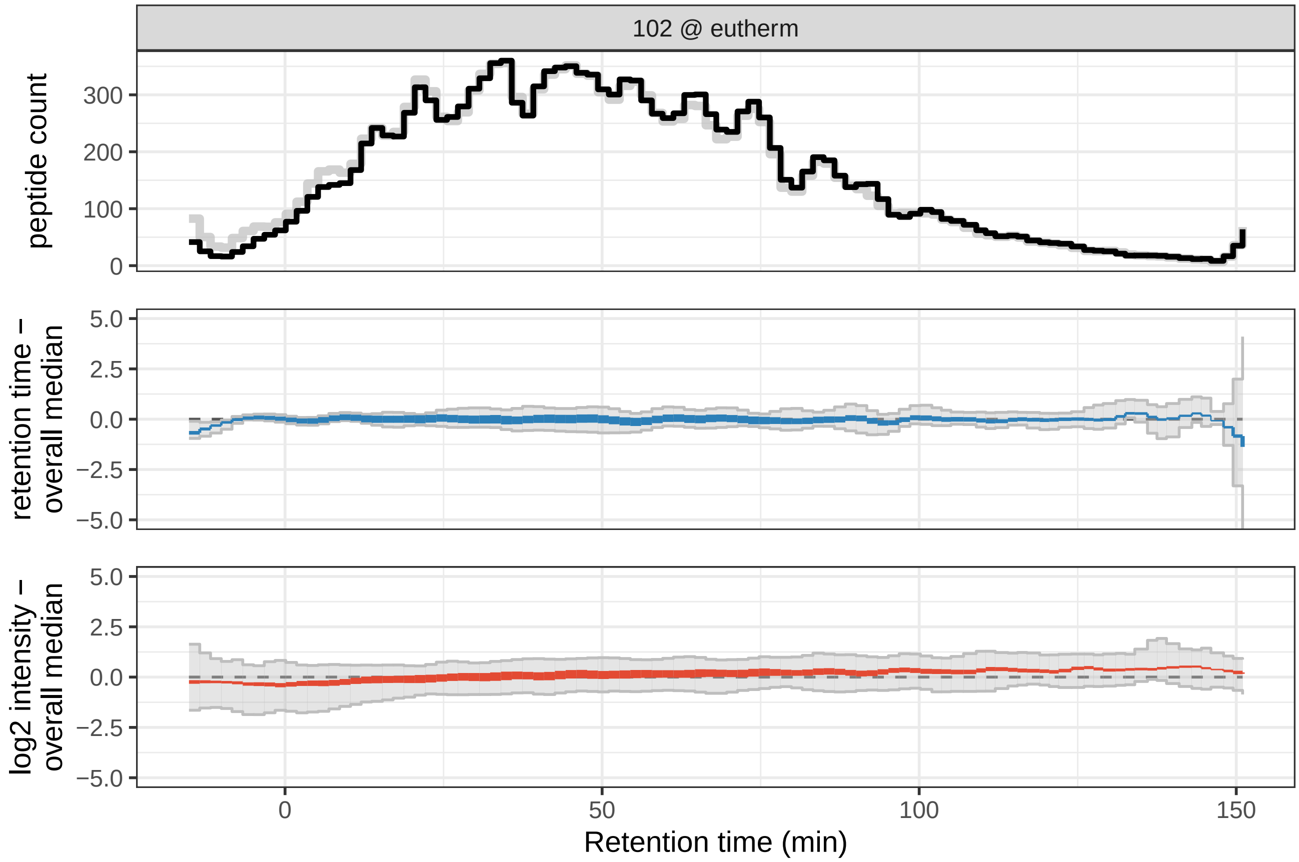
102 @ eutherm
peptide count
retention time – overall median
log2 intensity – overall median
Retention time (min)

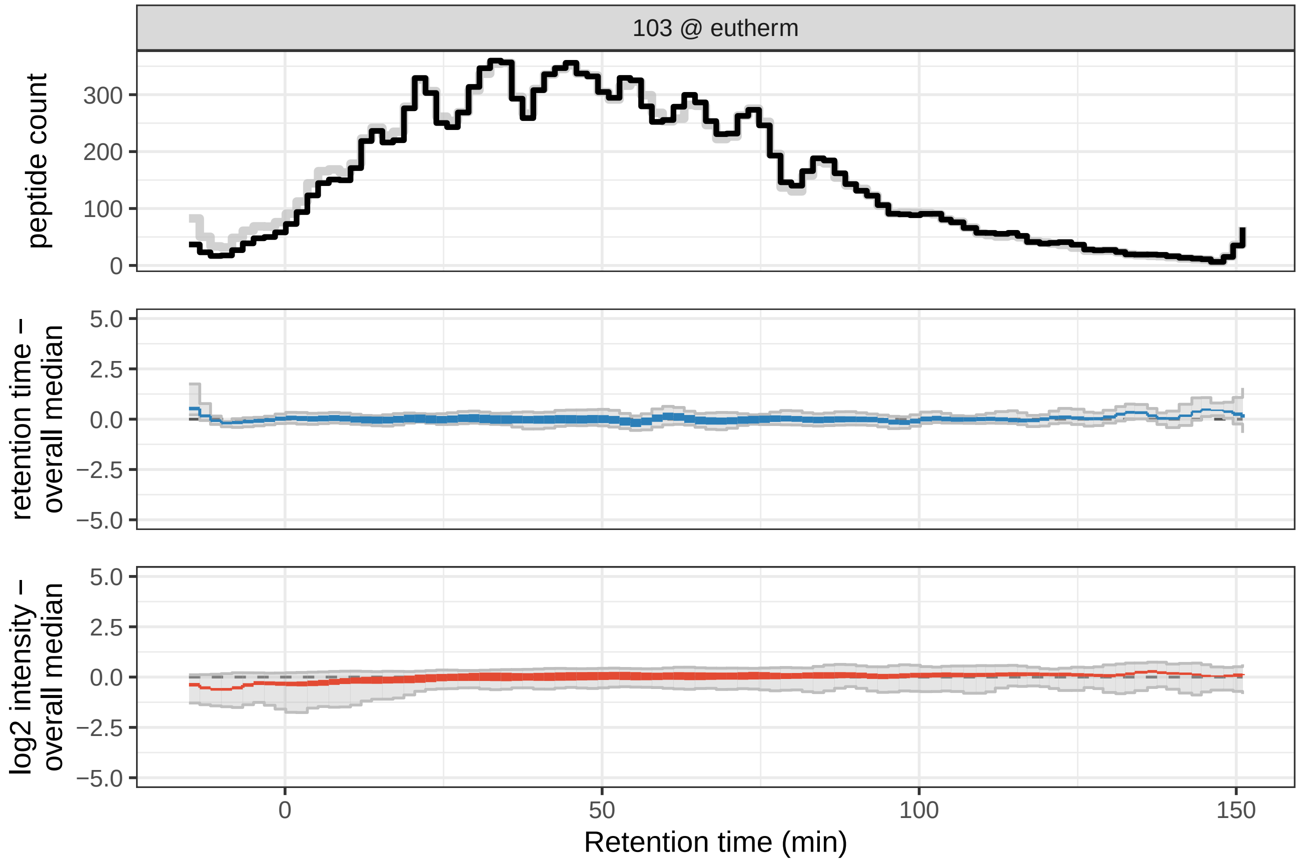
103 @ eutherm
peptide count
retention time – overall median
log2 intensity – overall median
Retention time (min)

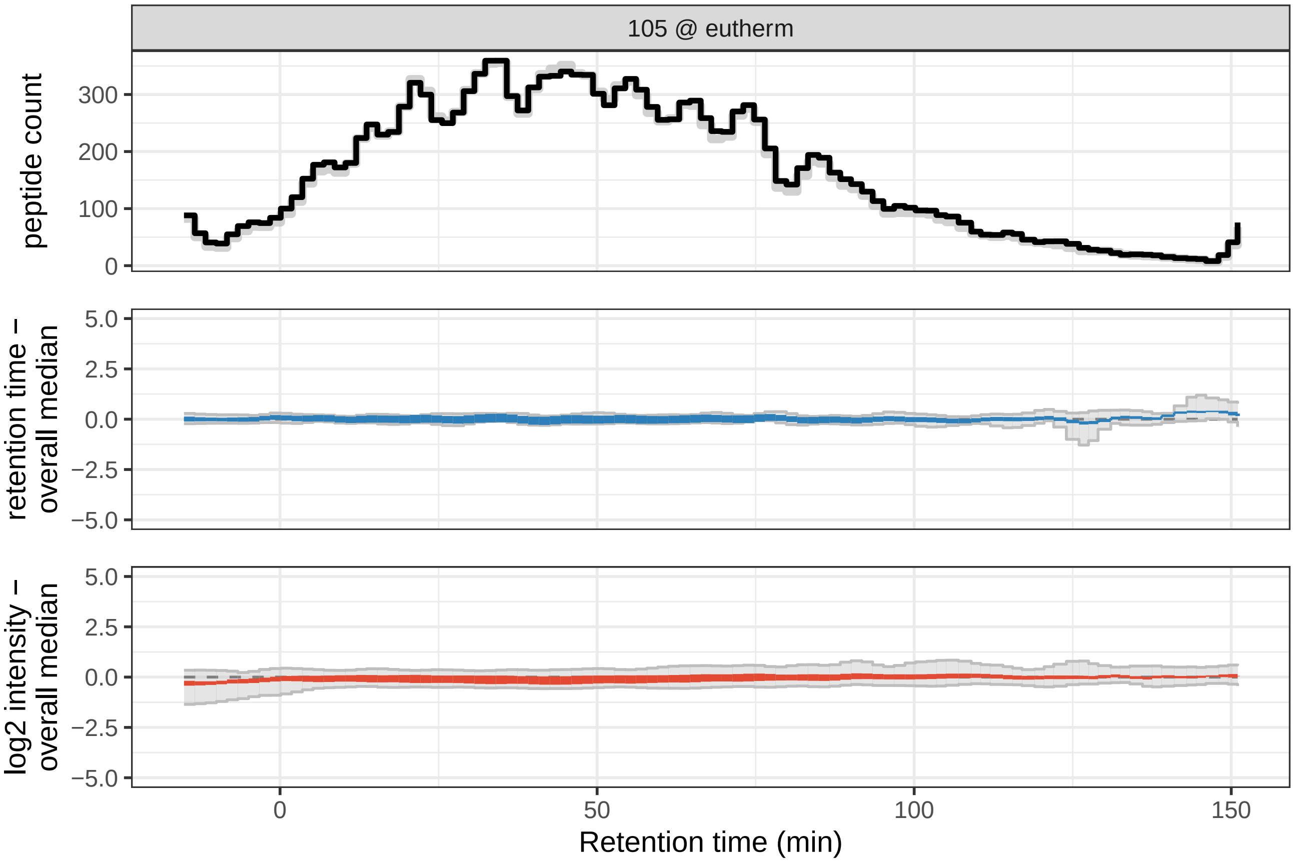
105 @ eutherm
peptide count
300
200
100
0
retention time –
overall median
5.0
2.5
0.0
−2.5
−5.0
log2 intensity –
overall median
5.0
2.5
0.0
−2.5
−5.0
0
50
100
150
Retention time (min)

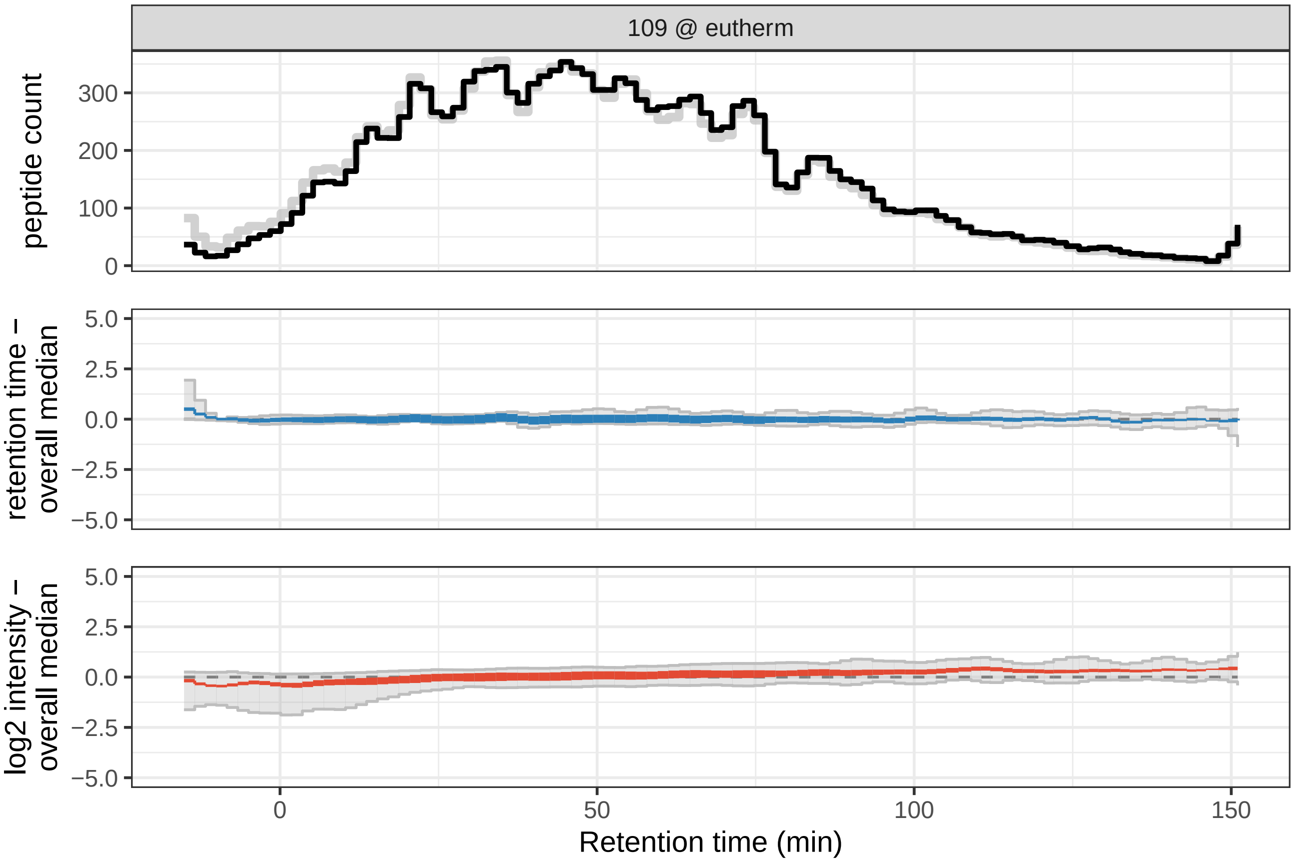
109 @ eutherm
peptide count
300
200
100
0
retention time –
overall median
5.0
2.5
0.0
−2.5
−5.0
log2 intensity –
overall median
5.0
2.5
0.0
−2.5
−5.0
0
50
100
150
Retention time (min)

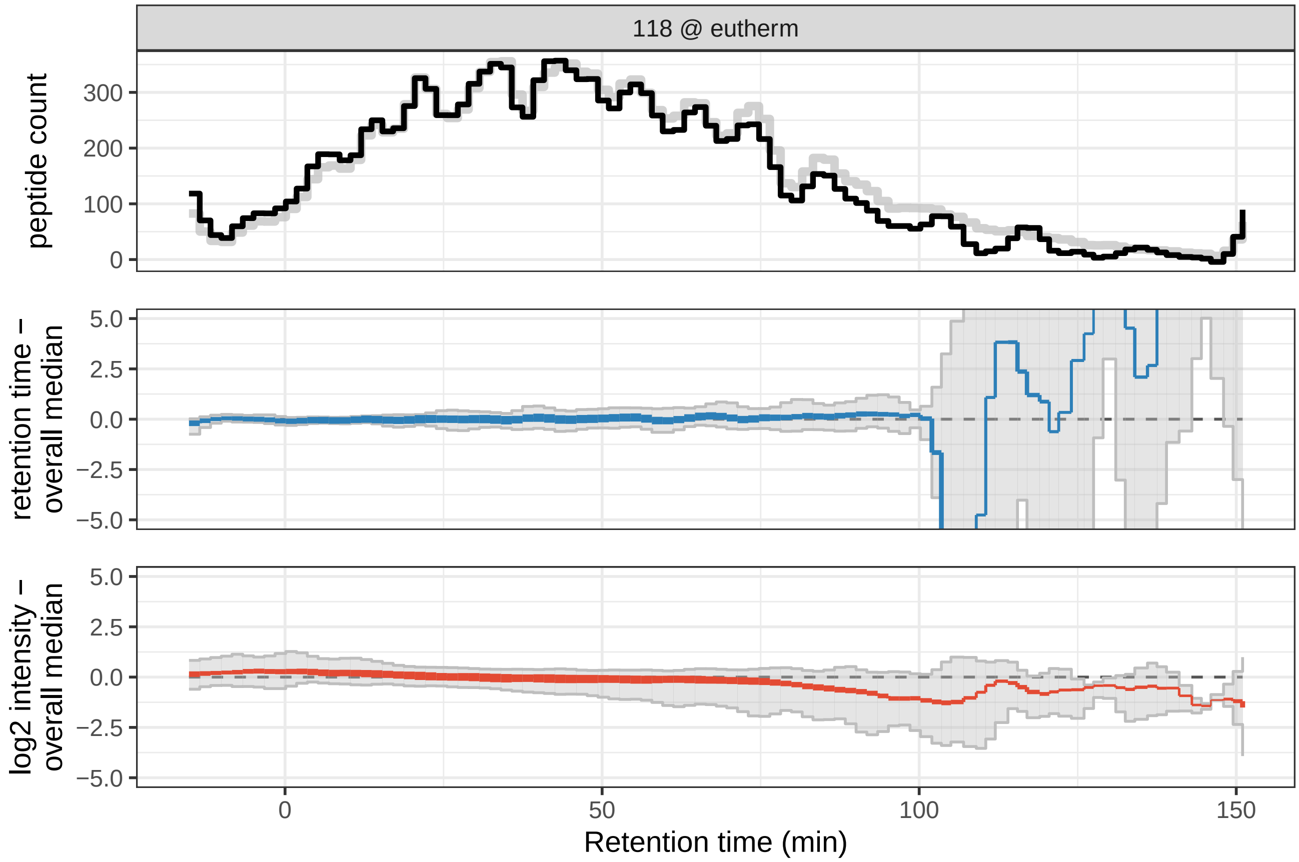
118 @ eutherm
peptide count
300
200
100
0
retention time –
overall median
5.0
2.5
0.0
−2.5
−5.0
log2 intensity –
overall median
5.0
2.5
0.0
−2.5
−5.0
0
50
100
150
Retention time (min)

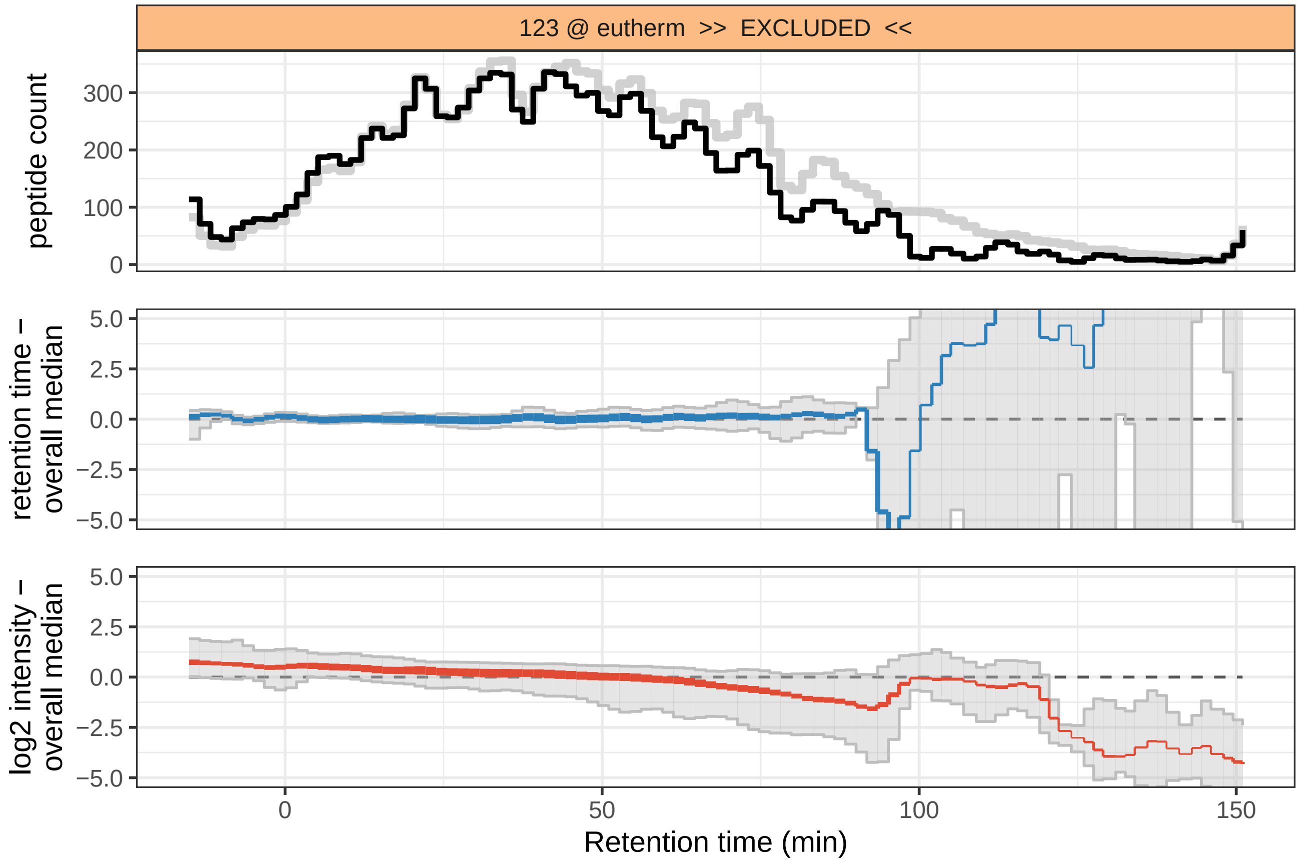
123 @ eutherm >> EXCLUDED <<
peptide count
300
200
100
0
retention time –
overall median
5.0
2.5
0.0
−2.5
−5.0
log2 intensity –
overall median
5.0
2.5
0.0
−2.5
−5.0
0
50
100
150
Retention time (min)

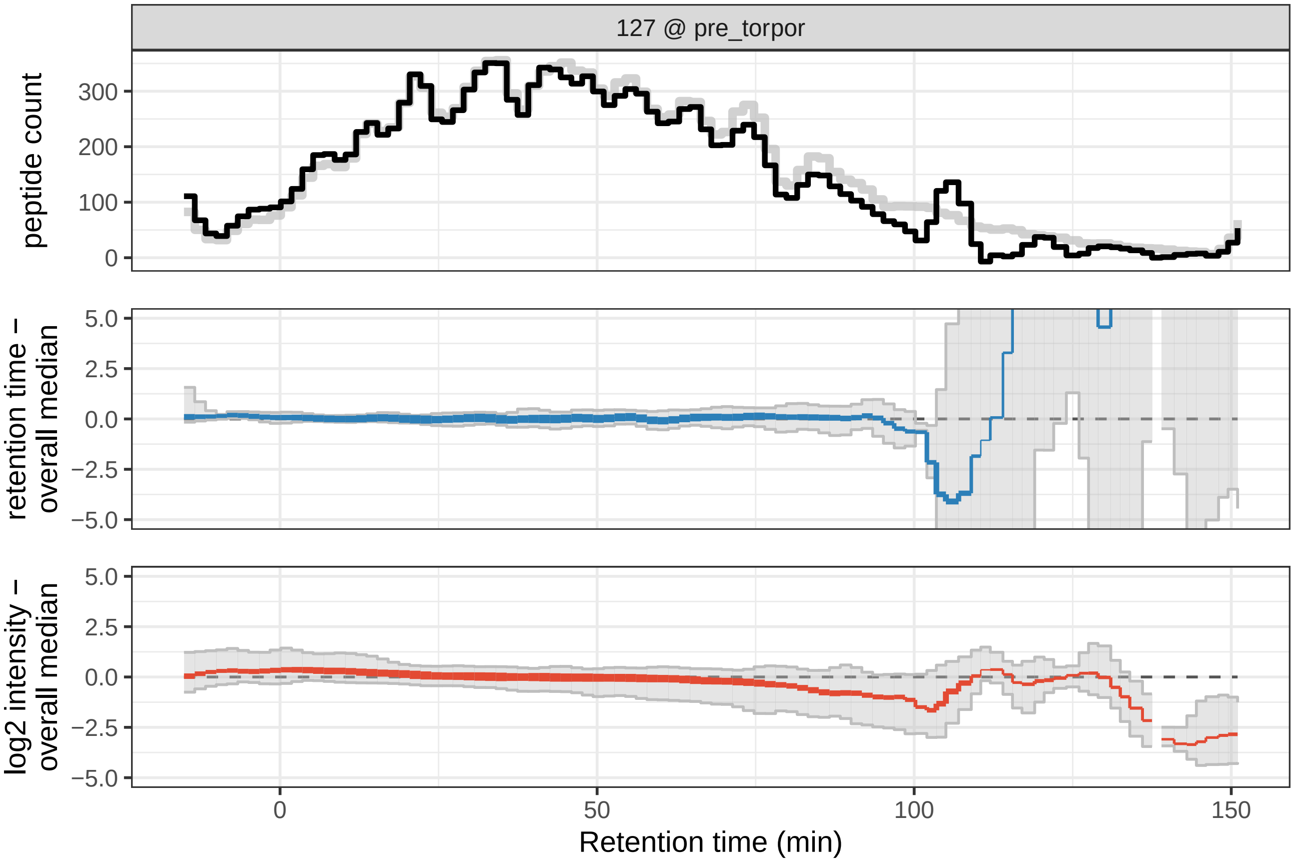
127 @ pre_torpor
peptide count
retention time – overall median
log2 intensity – overall median
Retention time (min)

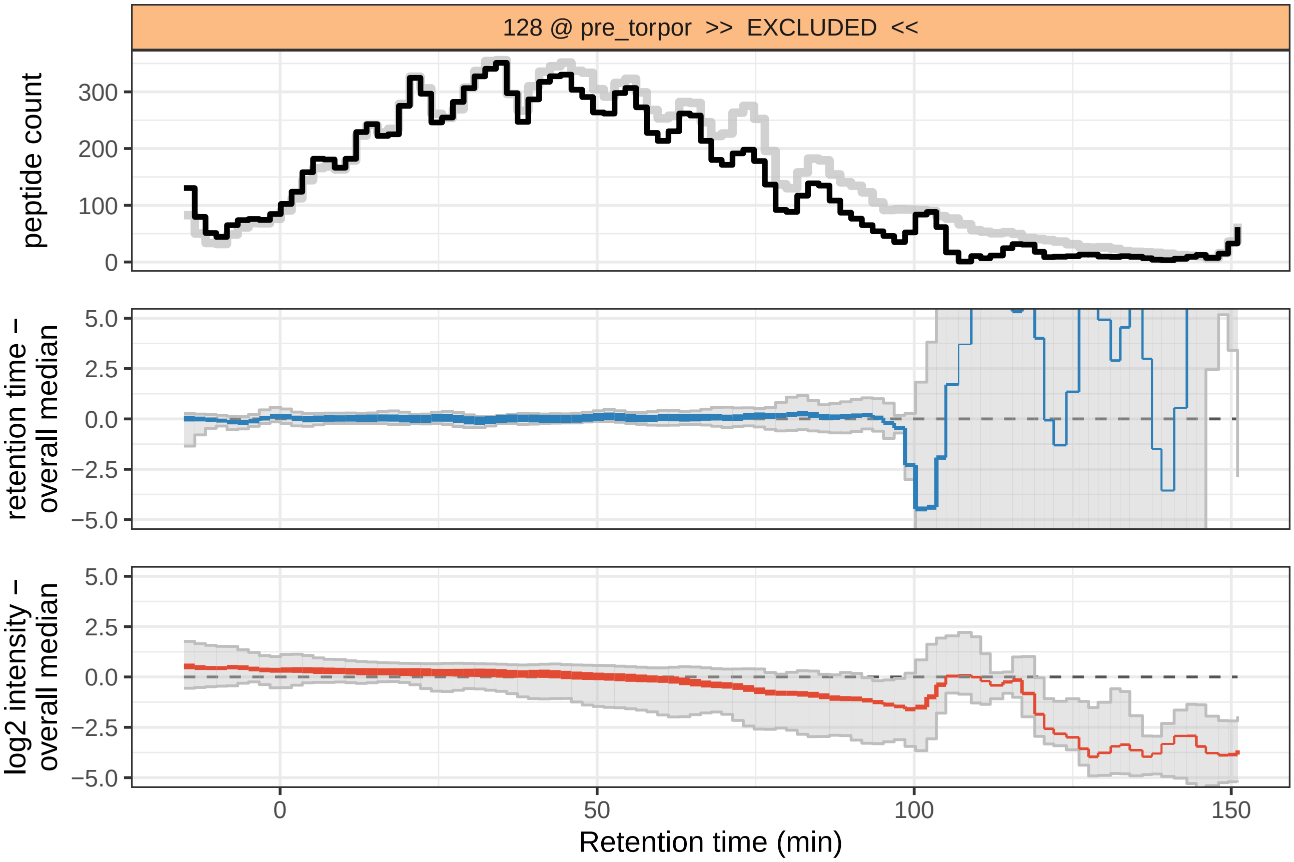
128 @ pre_torpor >> EXCLUDED <<
peptide count
retention time – overall median
log2 intensity – overall median
Retention time (min)

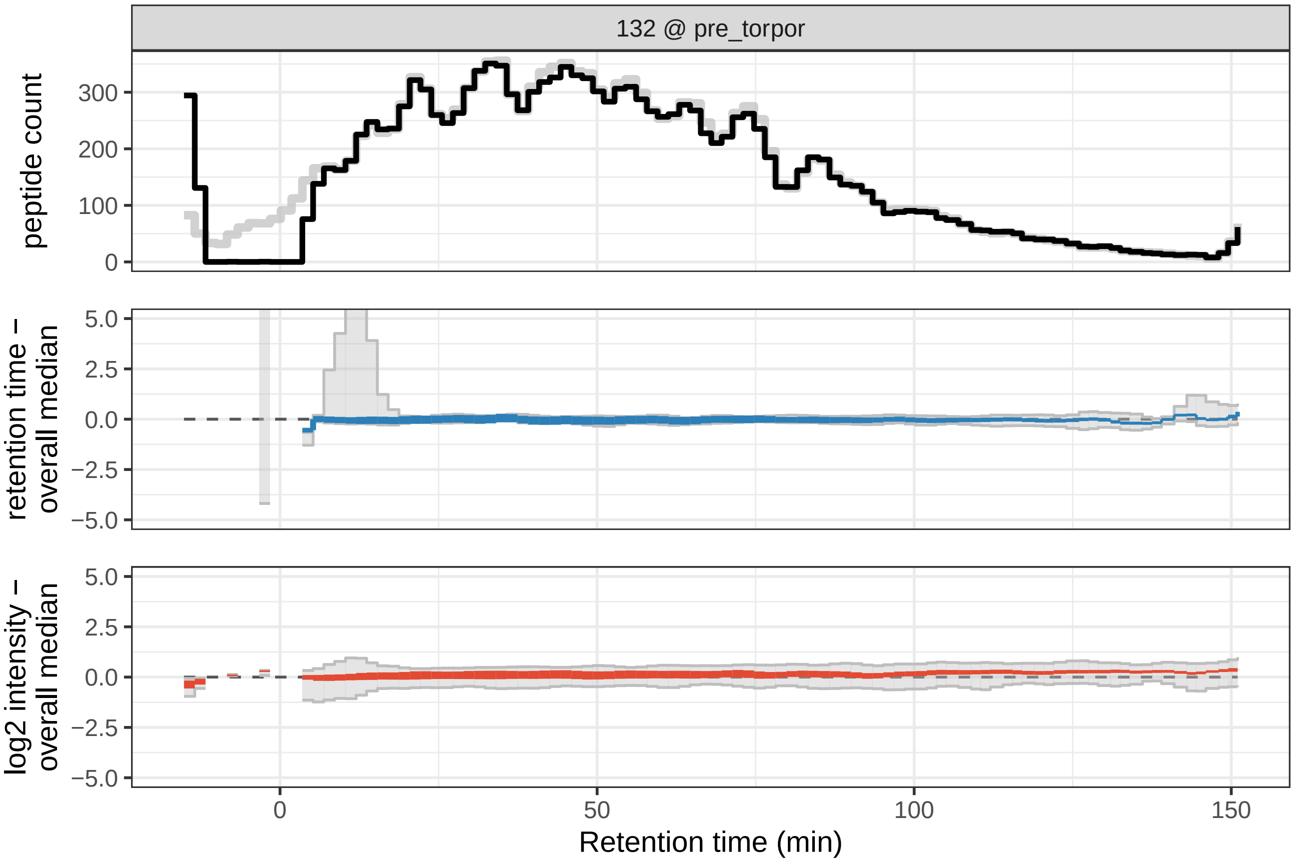
132 @ pre_torpor
peptide count
retention time – overall median
log2 intensity – overall median
Retention time (min)

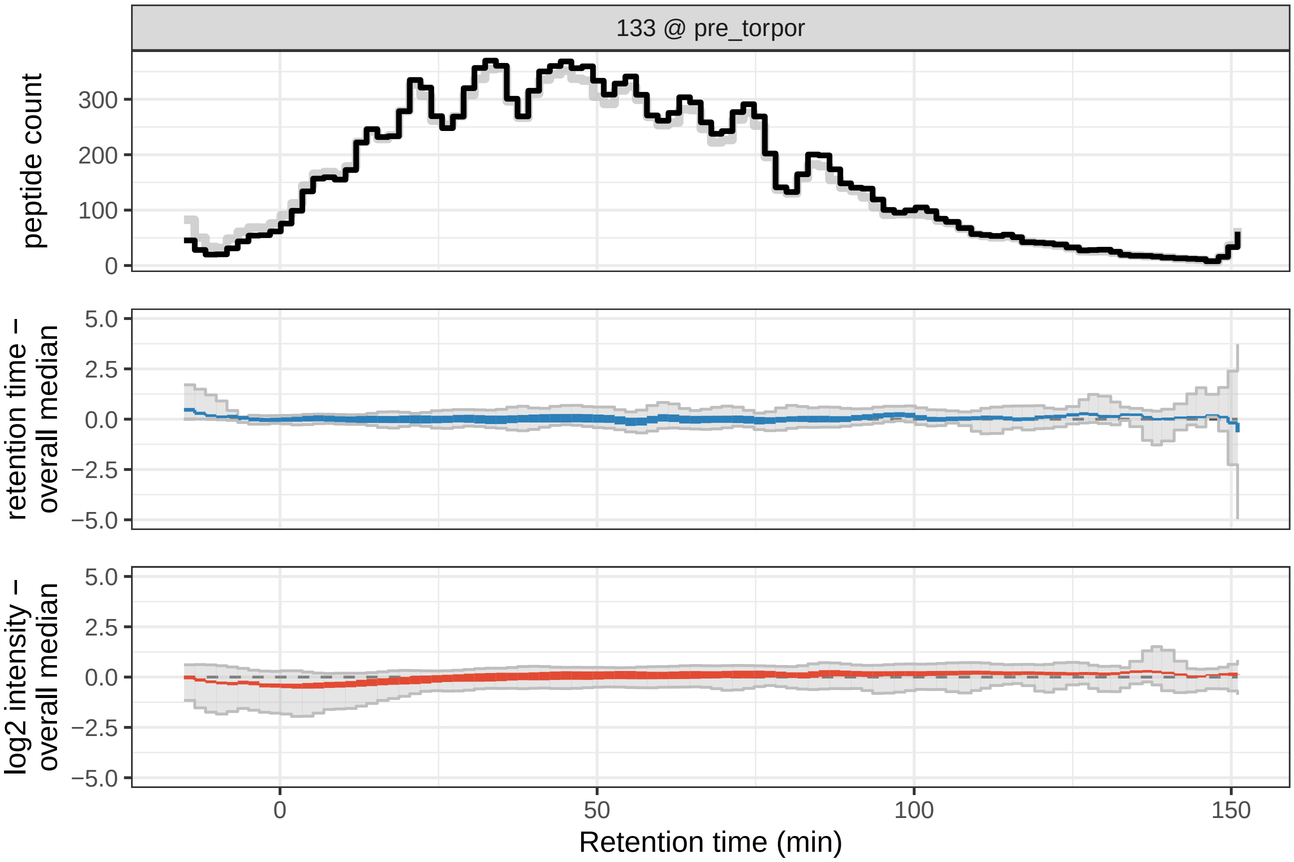
133 @ pre_torpor
peptide count
retention time – overall median
log2 intensity – overall median
Retention time (min)

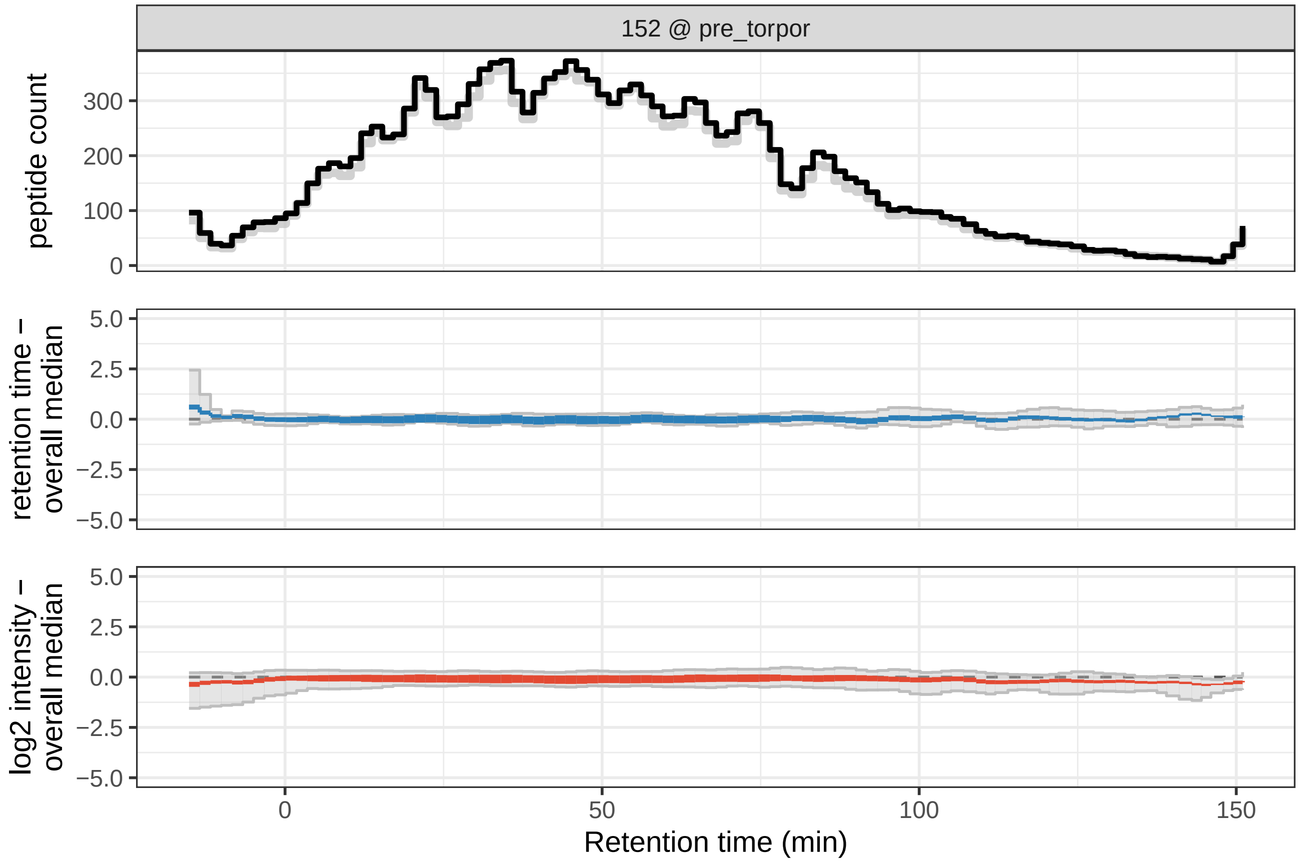
152 @ pre_torpor
peptide count
retention time – overall median
log2 intensity – overall median
Retention time (min)

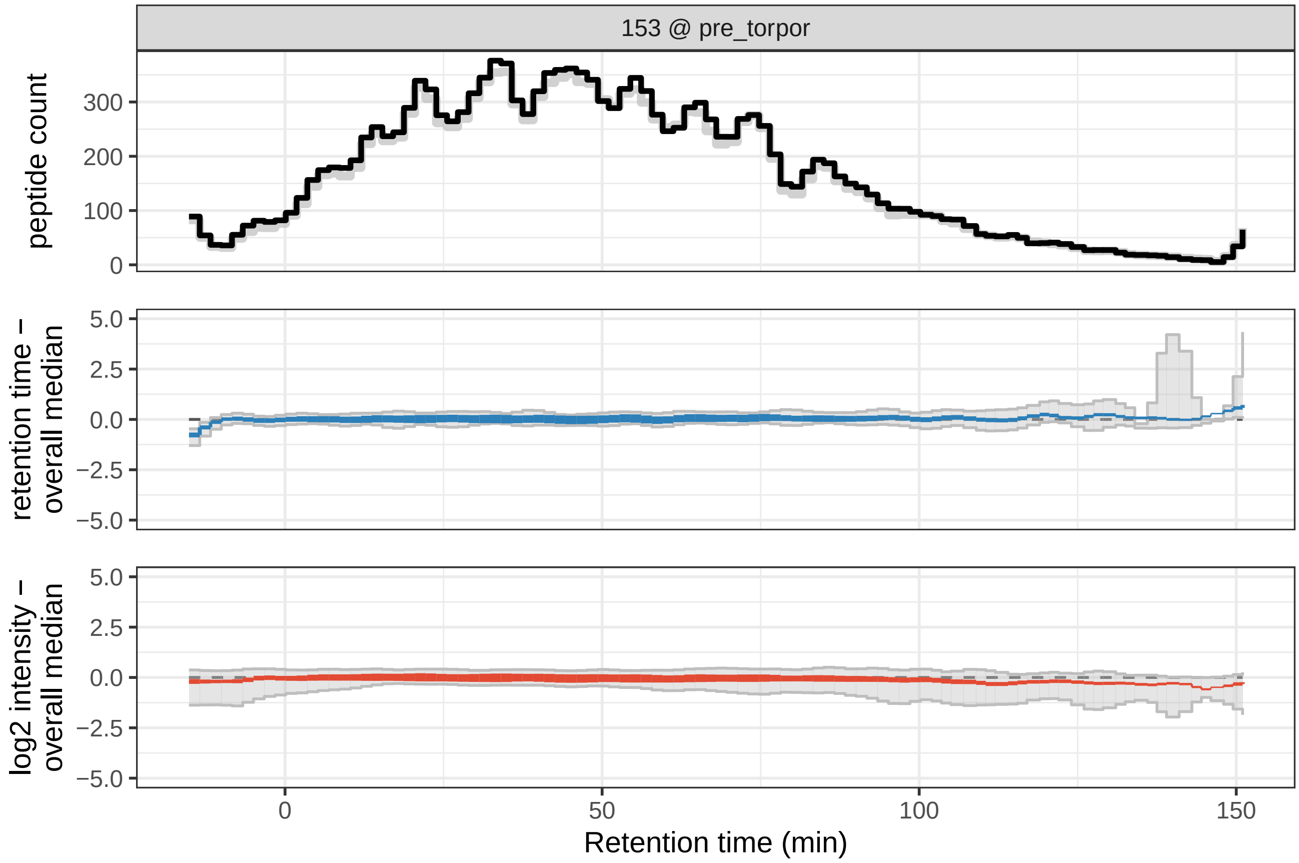
153 @ pre_torpor
peptide count
retention time – overall median
log2 intensity – overall median
Retention time (min)

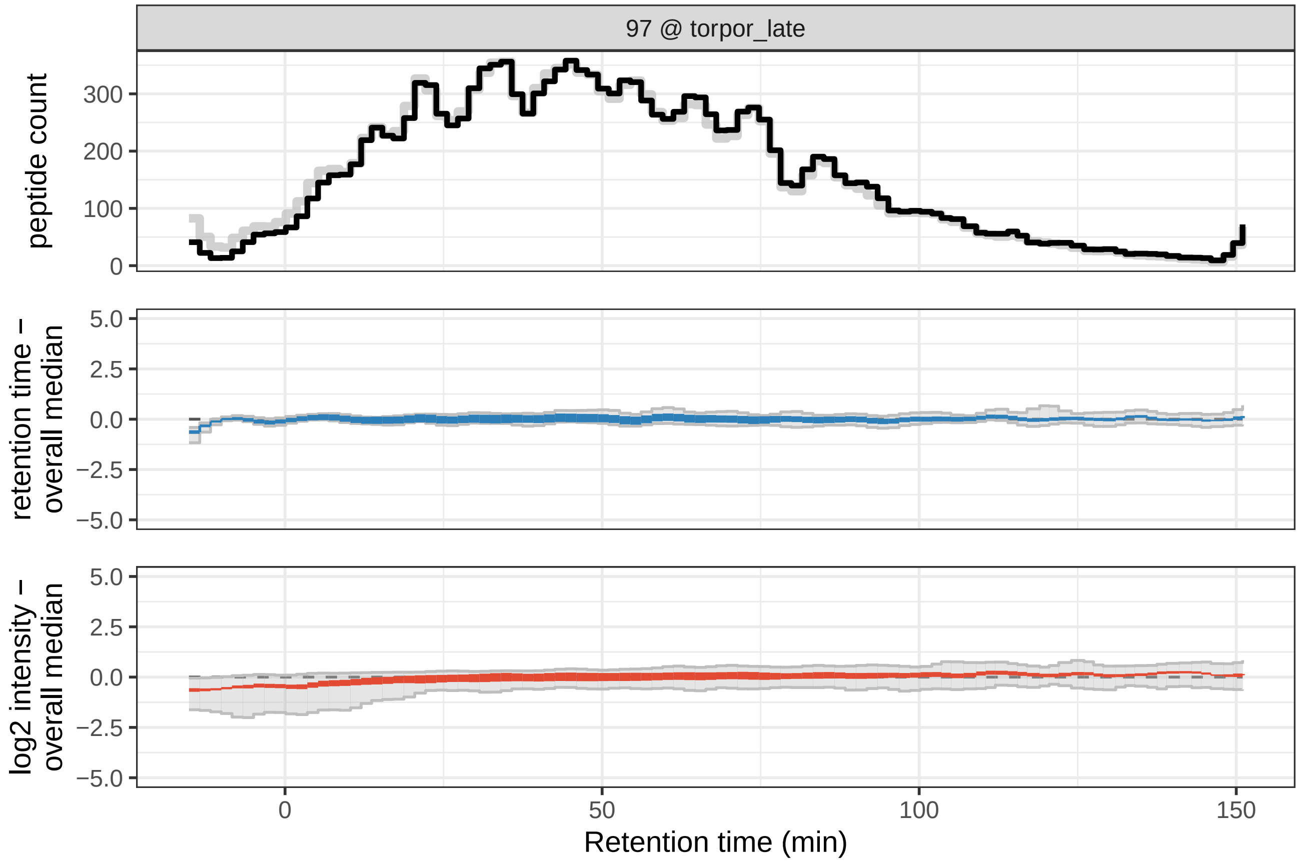
97 @ torpor_late
peptide count
retention time – overall median
log2 intensity – overall median
Retention time (min)

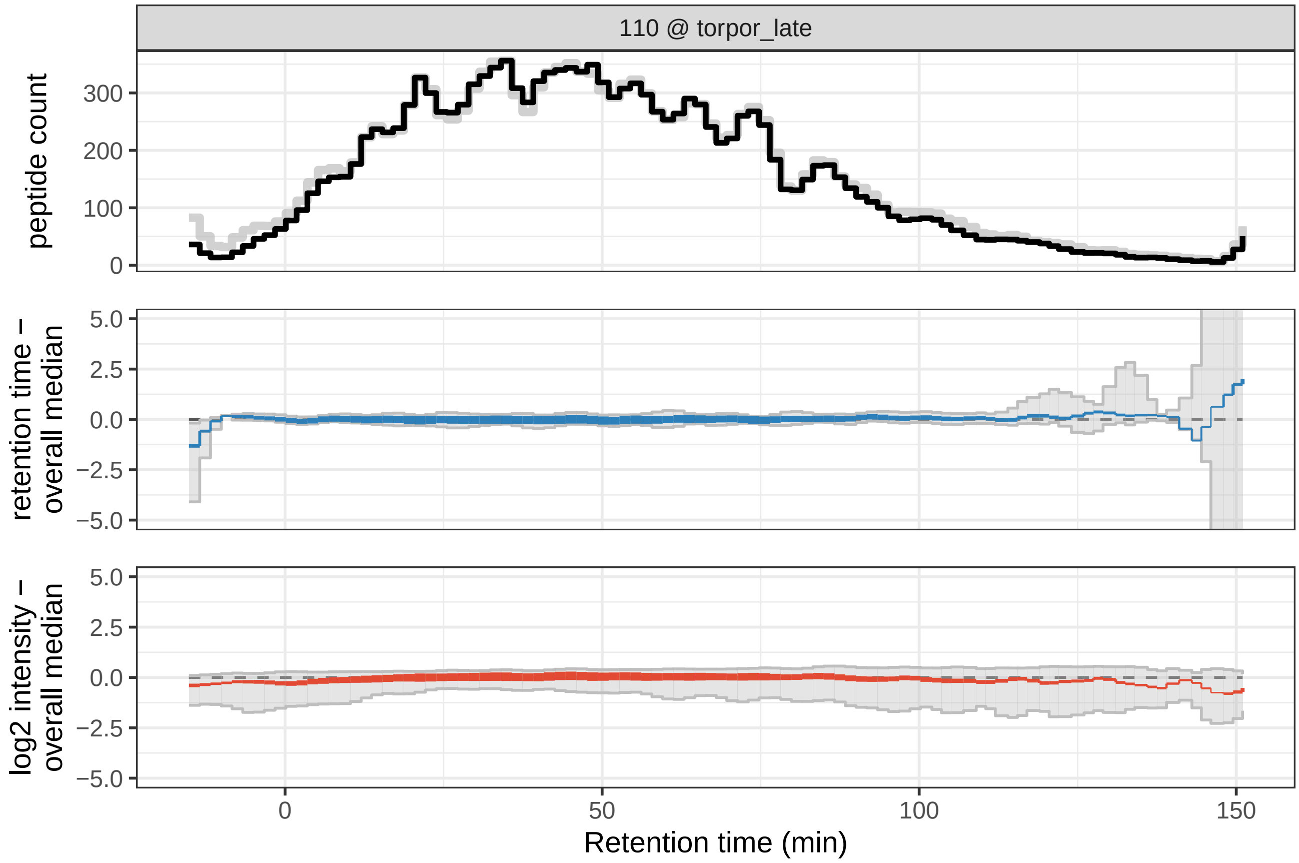
110 @ torpor_late
peptide count
retention time – overall median
log2 intensity – overall median
Retention time (min)

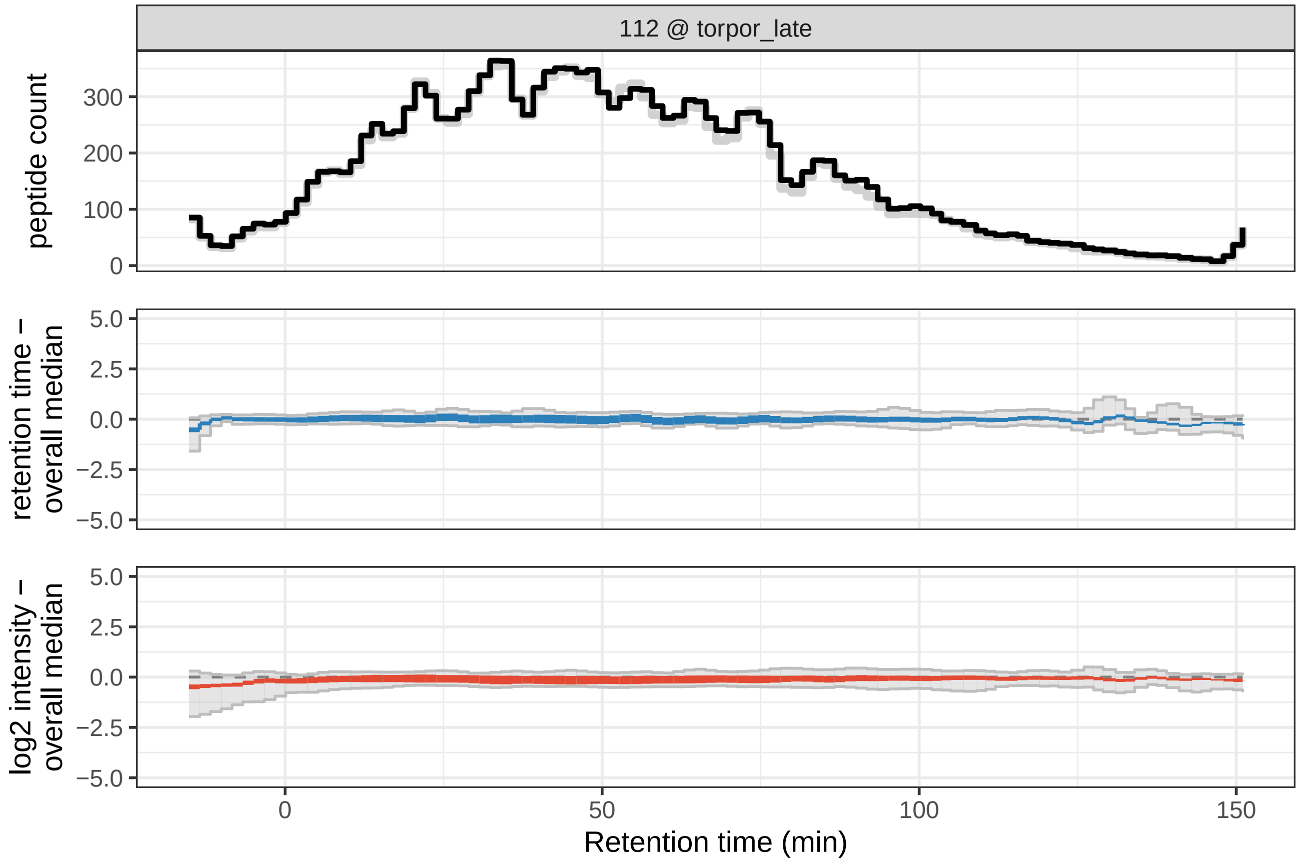
112 @ torpor_late
peptide count
retention time – overall median
log2 intensity – overall median
Retention time (min)

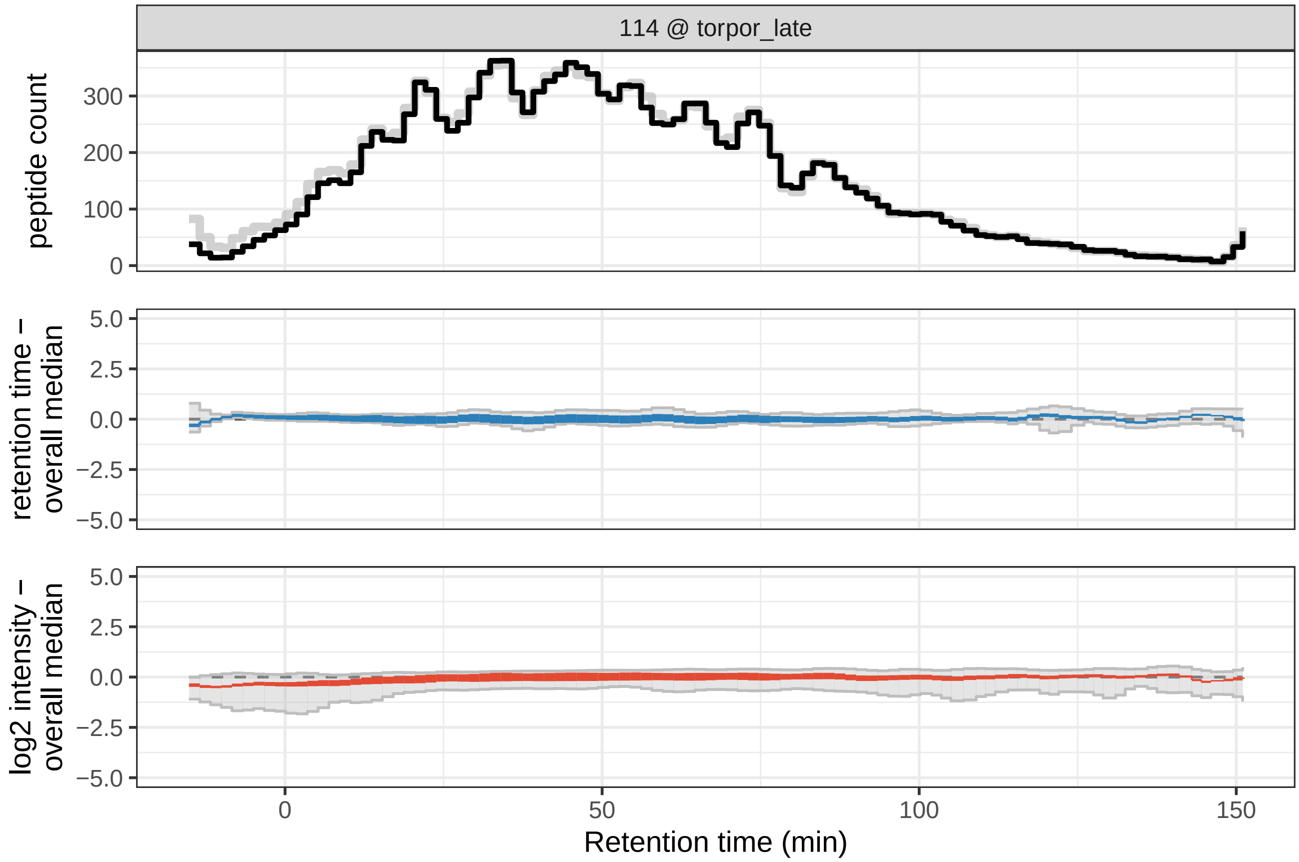
114 @ torpor_late
peptide count
retention time – overall median
log2 intensity – overall median
Retention time (min)

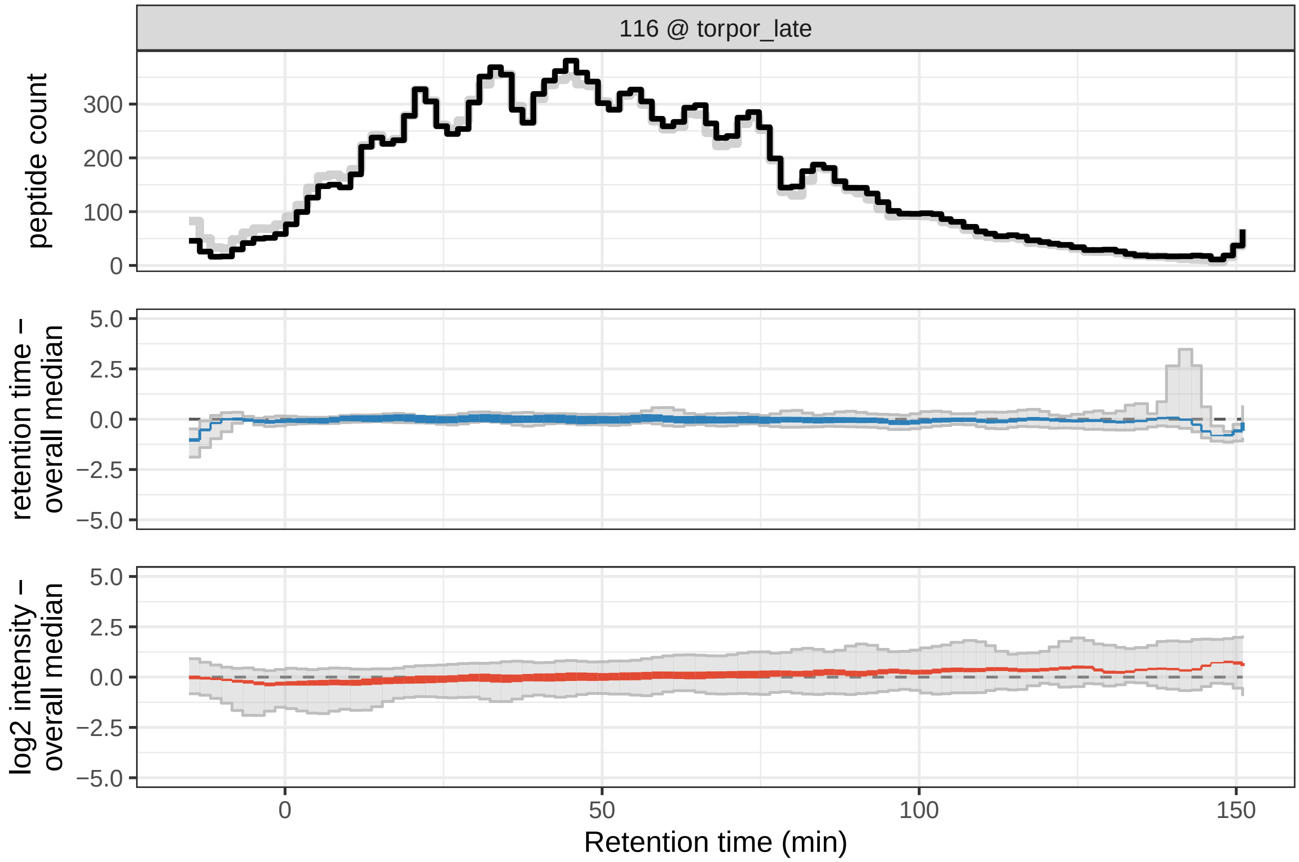
116 @ torpor_late
peptide count
300
200
100
0
retention time –
overall median
5.0
2.5
0.0
−2.5
−5.0
log2 intensity –
overall median
5.0
2.5
0.0
−2.5
−5.0
0
50
100
150
Retention time (min)

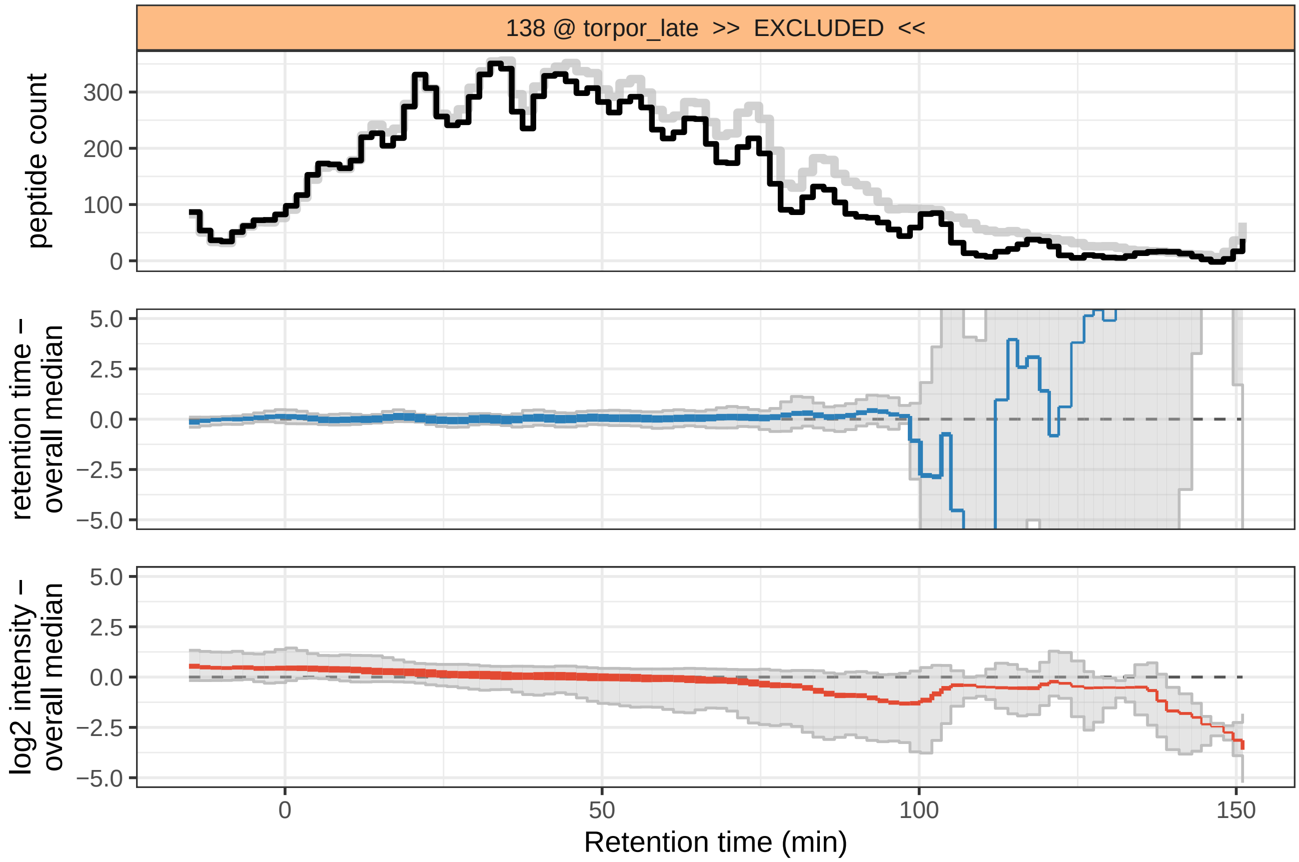
138 @ torpor_late >> EXCLUDED <<
peptide count
300
200
100
0
retention time –
overall median
5.0
2.5
0.0
−2.5
−5.0
log2 intensity –
overall median
5.0
2.5
0.0
−2.5
−5.0
0
50
100
150
Retention time (min)

1.5 variation among replicates

The reproducibility of replicate measurements is expressed in three different analyses. First, the difference between peptide intensities in each sample are compared to the mean value among all replicates (foldchange distributions). Next, the Coefficient of Variation (CoV) is used as a metric for reproducibility to explore how much the CoV within a sample group can be improved by removing a single sample (eg; if CoV strongly improved after removing sample s, it could be regarded as an outlier). Finally, the CoV within each sample group is visualized as a boxplot and a violin plot, figures commonly seen in proteomics literature and useful for comparing across experiments (of similar protocol).

1.5.1 within-group foldchange distributions

The foldchange of all peptides in a sample is compared to their respective mean value over all samples in the group. This visualizes how strongly each sample deviates from other samples in the same group which helps identify outlier samples. The same data was used as detailed in the "retention time" section above. Samples marked as 'exclude' in the provided sample metadata table are visualized as dashed lines.

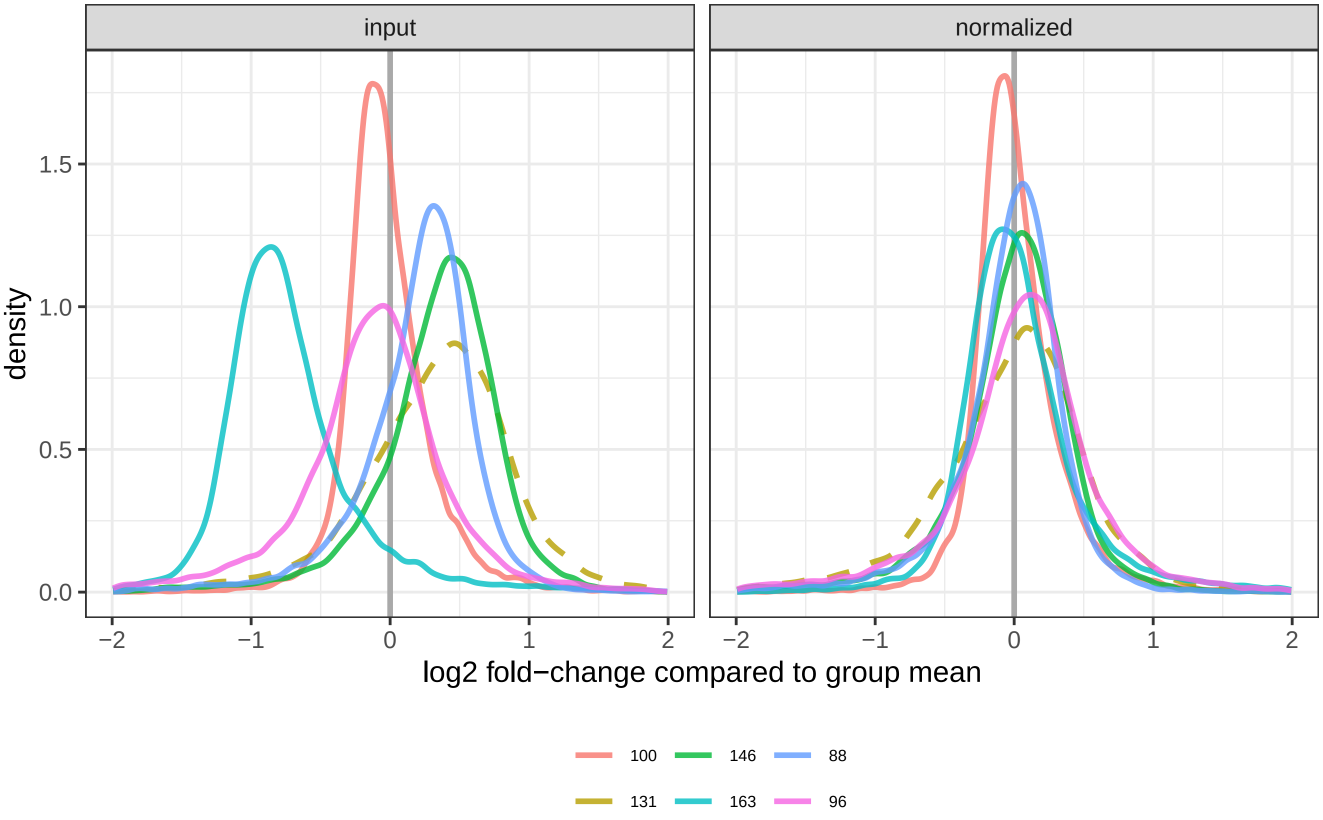

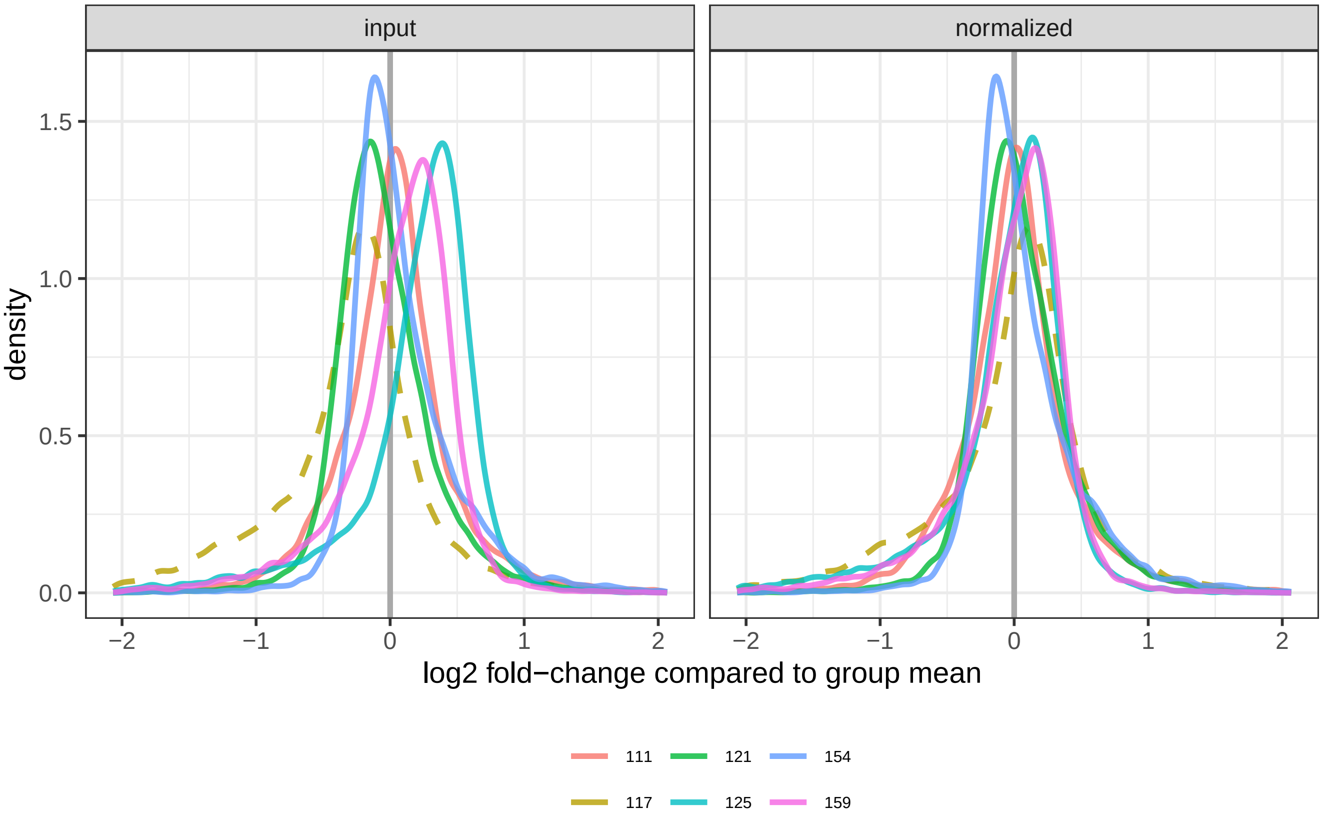
input
normalized
density
1.5
1.0
0.5
0.0
−2
−1
0
1
2
log2 fold−change compared to group mean
111
121
154
117
125
159

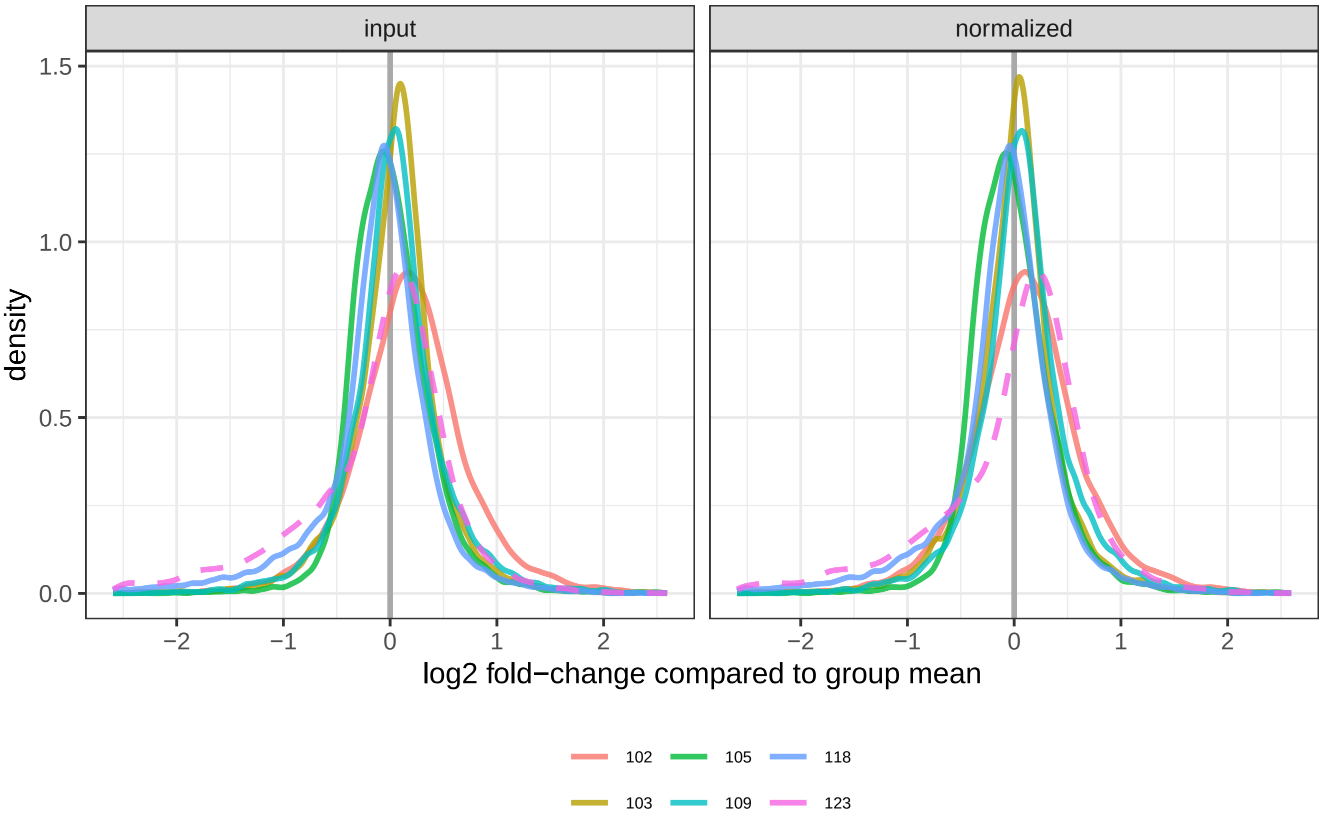
input
normalized
density
1.5
1.0
0.5
0.0
−2
−1
0
1
2
log2 fold−change compared to group mean
102
105
118
103
109
123

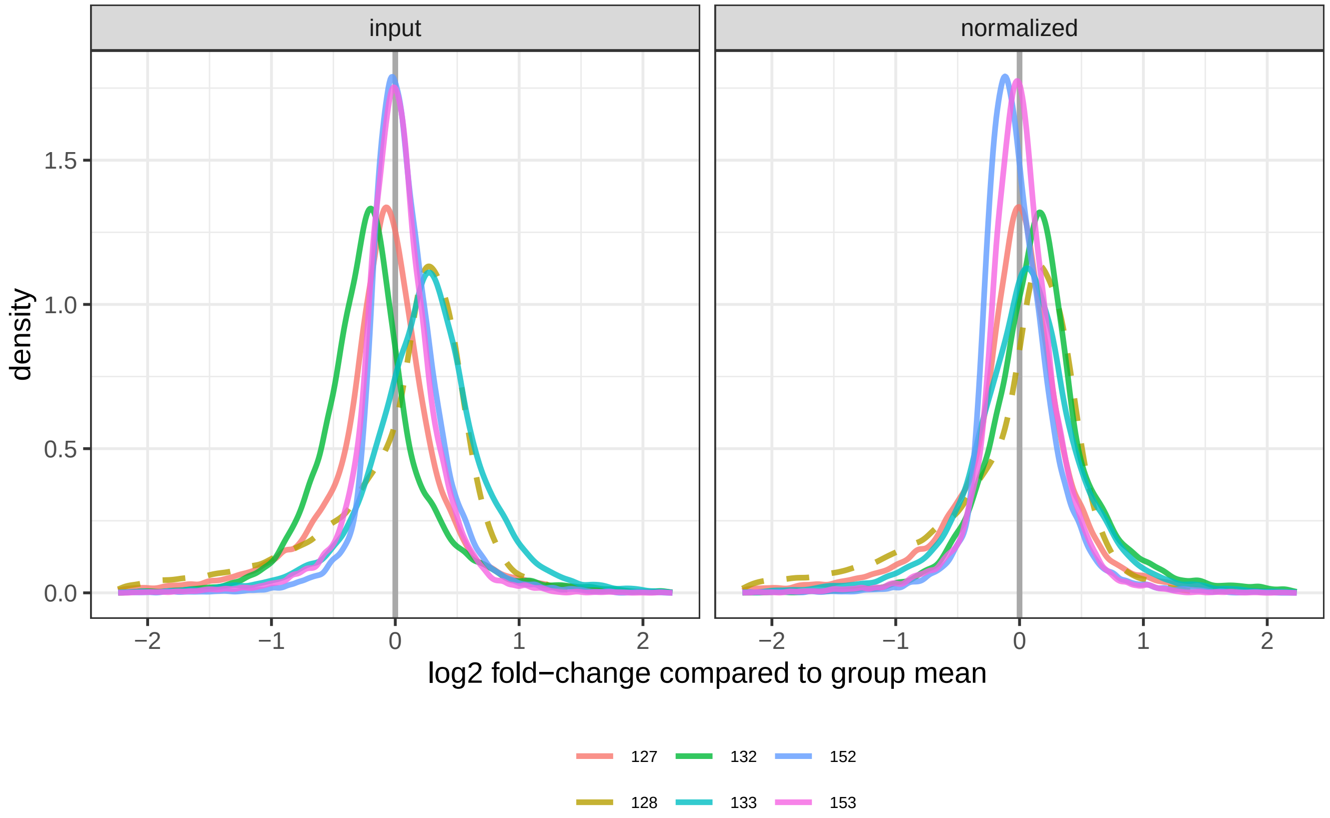
input
normalized
density
1.5
1.0
0.5
0.0
−2
−1
0
1
2
log2 fold−change compared to group mean
127
132
152
128
133
153

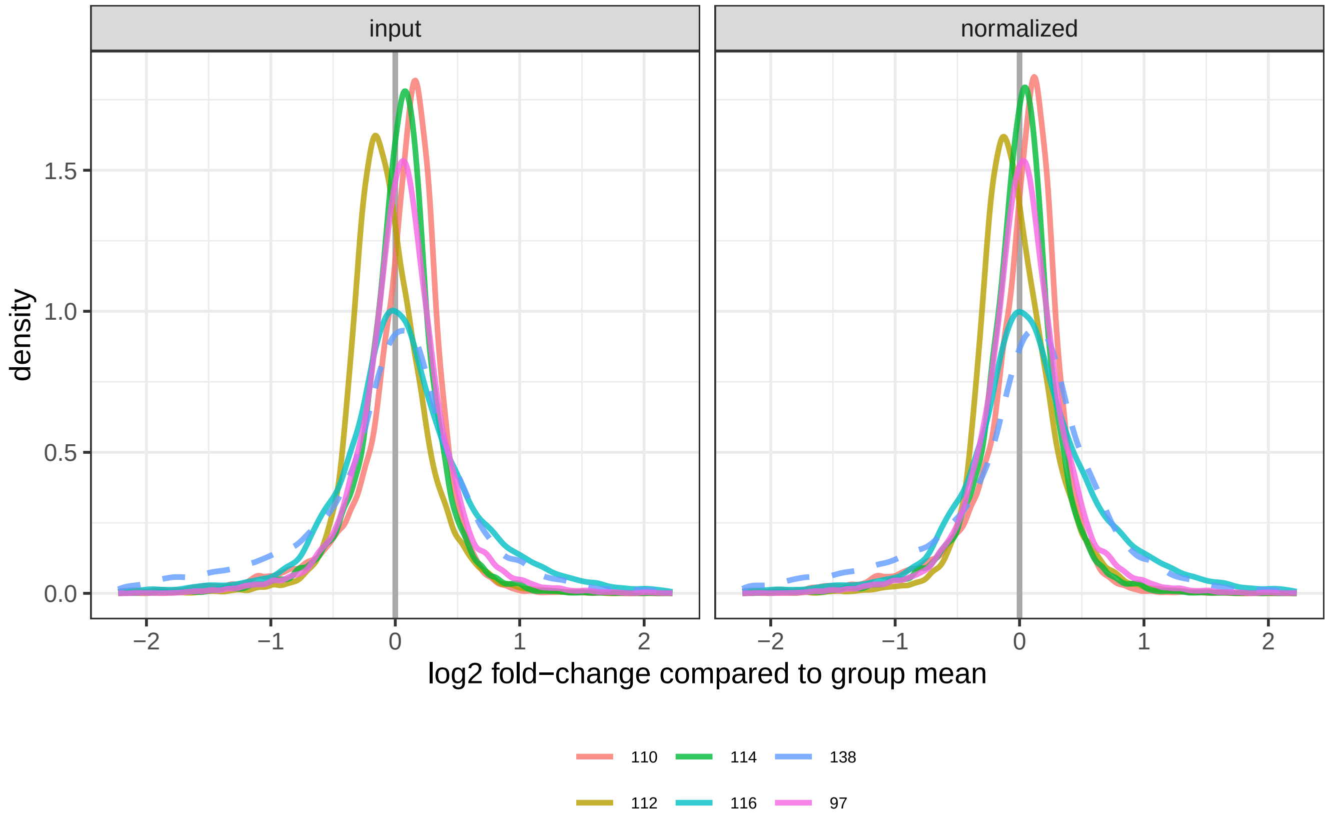
input
normalized
density
1.5
1.0
0.5
0.0
−2
−1
0
1
2
log2 fold−change compared to group mean
110
114
138
112
116
97

1.5.2 CoV, leave-one-out

progress: leave-one-out CoV plot computations took 11 seconds The figures below describe the effect of removing a particular sample prior to within-group Coefficient of Variation (CoV) computation. The lower the CoV distribution is for a sample, the better reproducibility we get by excluding it. Only sample groups with at least 4 replicates can be used for this analysis, so 3 samples remain after leaving one out. Samples marked as 'exclude' in the provided sample metadata are included in these analyses (shown as dashed lines), and only peptides with at least 3 data points across replicates samples (after leave-one-out) are used for each CoV computation.

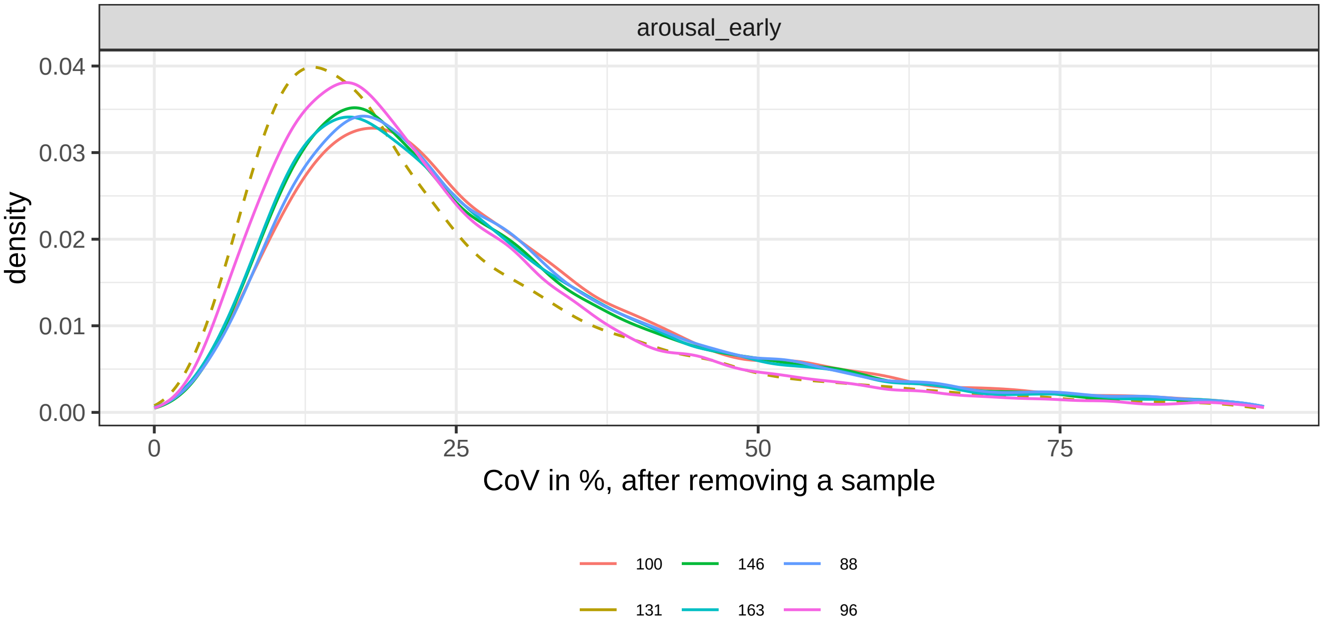

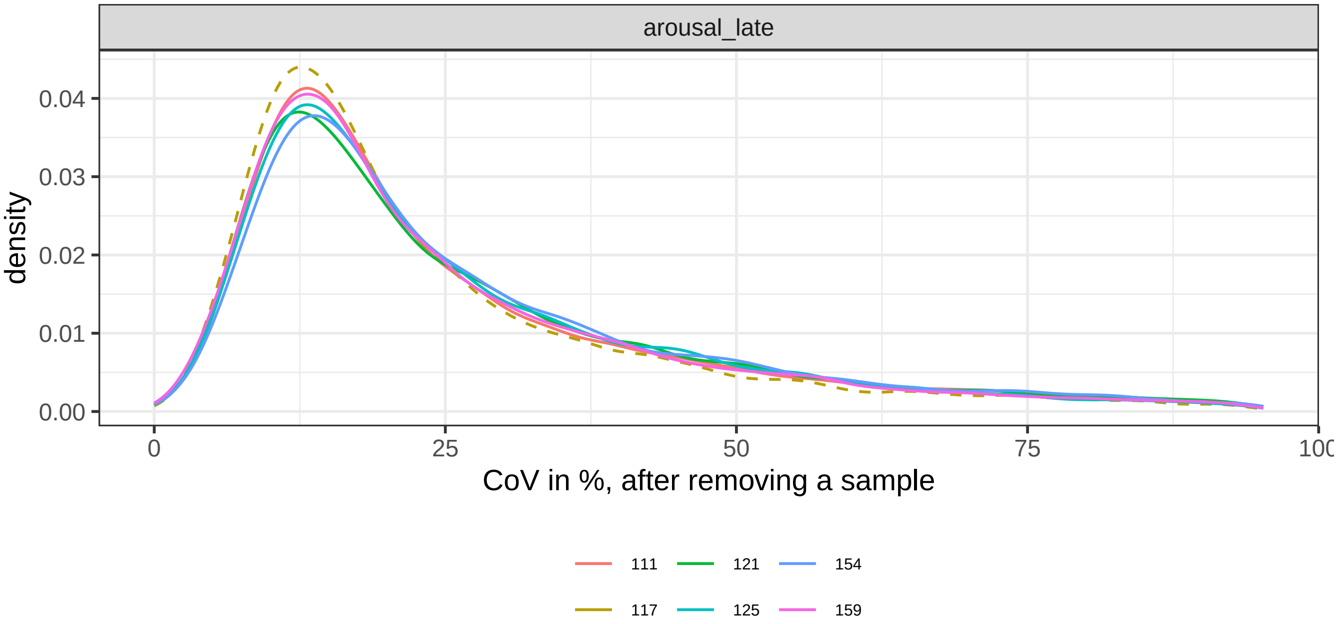

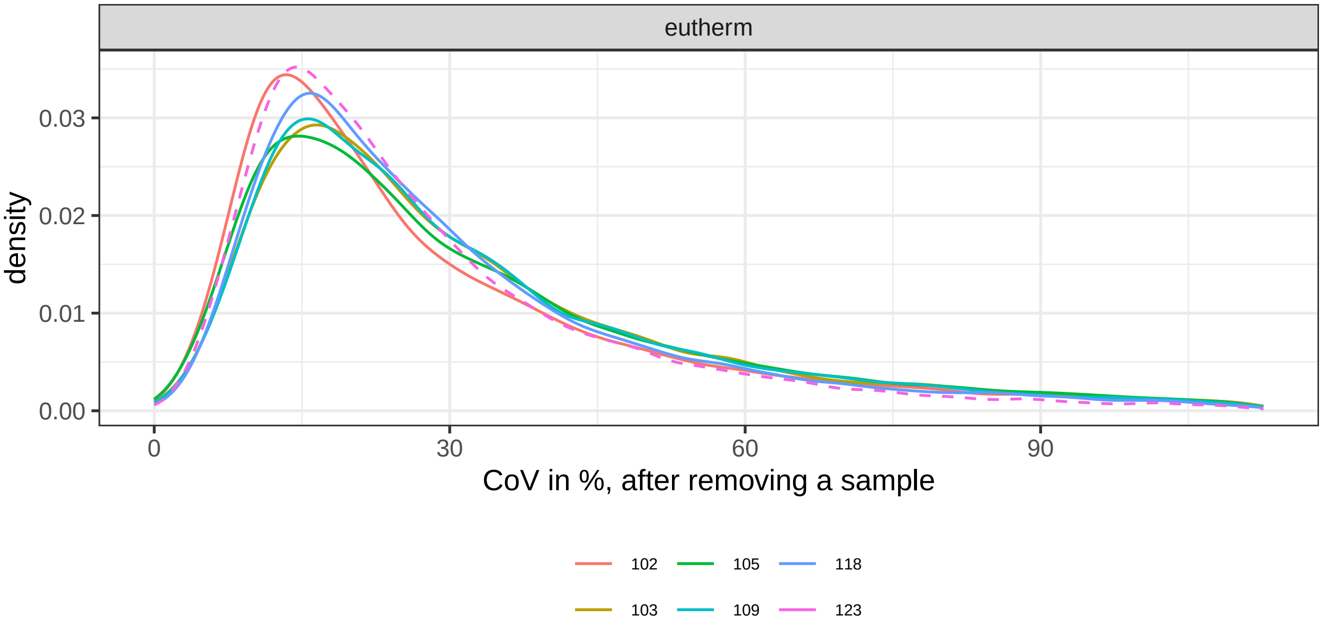
eutherm
density
0.03
0.02
0.01
0.00
0
30
60
90
CoV in %, after removing a sample
102
105
118
103
109
123

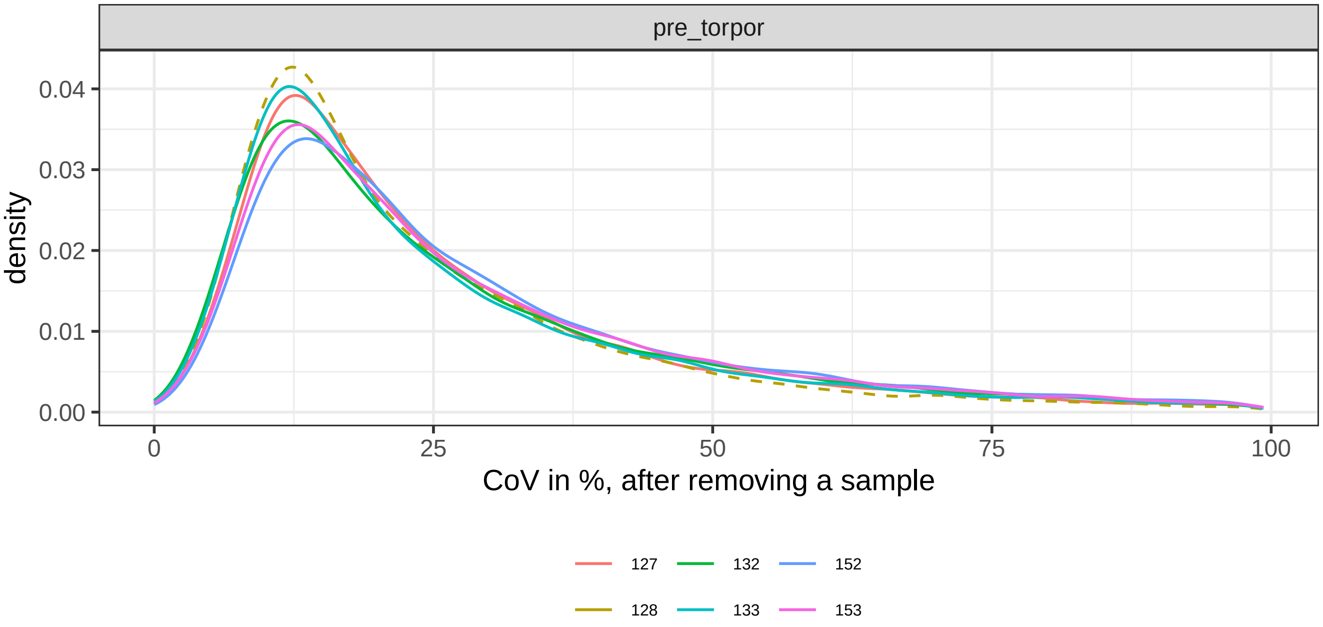
pre_torpor
density
0.04
0.03
0.02
0.01
0.00
0
25
50
75
100
CoV in %, after removing a sample
127
132
152
128
133
153

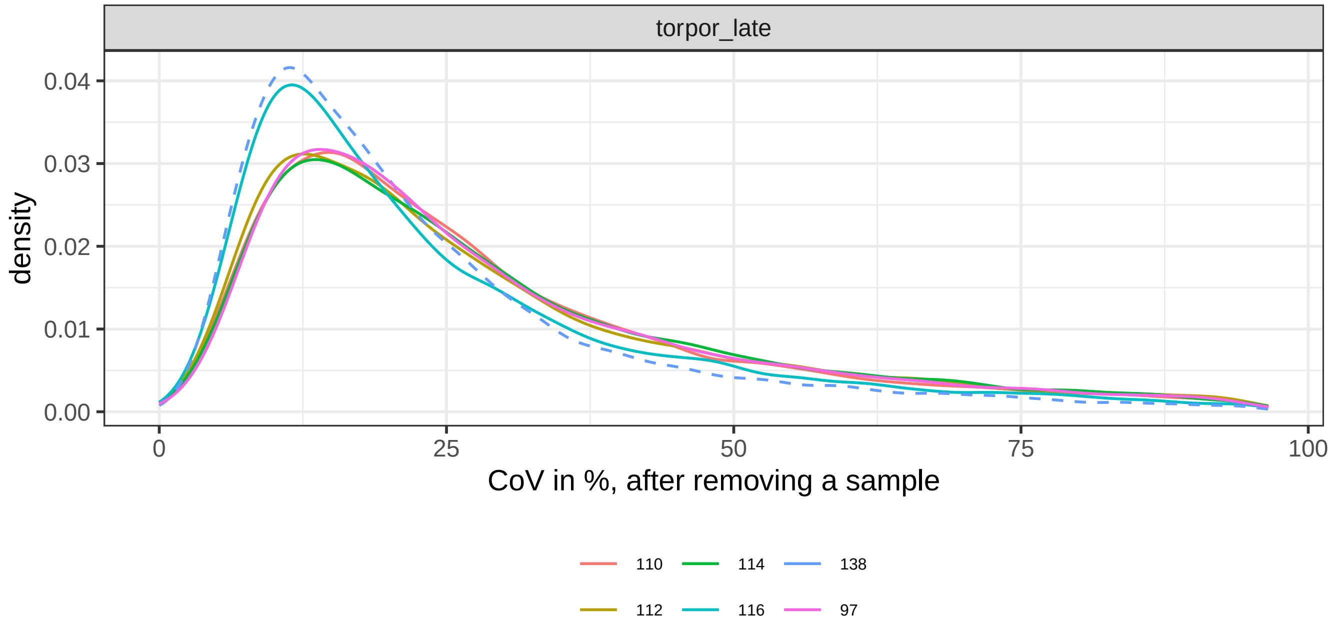

torpor_late
density
0.04
0.03
0.02
0.01
0.00
0
25
50
75
100
CoV in %, after removing a sample
110
114
138
112
116
97

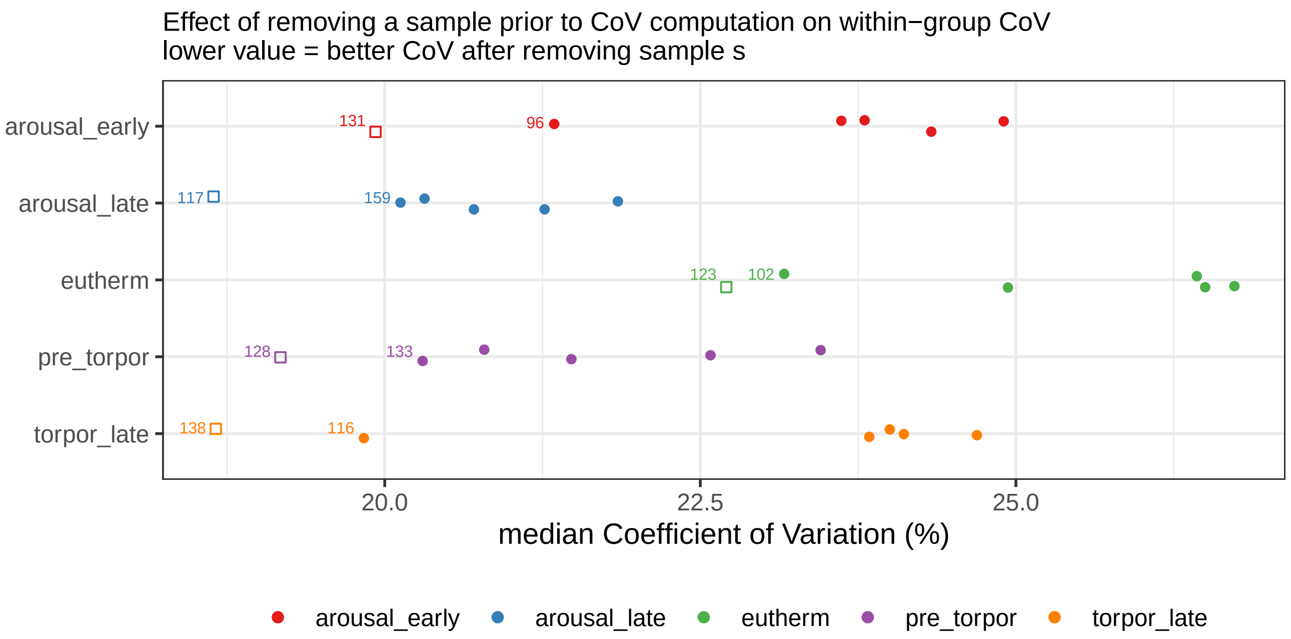

Effect of removing a sample prior to CoV computation on within−group CoV
lower value = better CoV after removing sample s
arousal_early
arousal_late
eutherm
pre_torpor
torpor_late
131
96
117
159
123
102
128
133
138
116
20.0
22.5
25.0
median Coefficient of Variation (%)
arousal_early
arousal_late
eutherm
pre_torpor
torpor_late

shortname	group	exclude	median CoV
131	arousal_early	TRUE	19.9
96	arousal_early	FALSE	21.3
146	arousal_early	FALSE	23.6
163	arousal_early	FALSE	23.8
88	arousal_early	FALSE	24.3
100	arousal_early	FALSE	24.9
117	arousal_late	TRUE	18.6
159	arousal_late	FALSE	20.1
111	arousal_late	FALSE	20.3
125	arousal_late	FALSE	20.7
121	arousal_late	FALSE	21.3
154	arousal_late	FALSE	21.8
123	eutherm	TRUE	22.7
102	eutherm	FALSE	23.2
118	eutherm	FALSE	24.9
105	eutherm	FALSE	26.4
109	eutherm	FALSE	26.5
103	eutherm	FALSE	26.7
128	pre_torpor	TRUE	19.2
133	pre_torpor	FALSE	20.3
127	pre_torpor	FALSE	20.8
132	pre_torpor	FALSE	21.5
153	pre_torpor	FALSE	22.6
152	pre_torpor	FALSE	23.5
138	torpor_late	TRUE	18.7
116	torpor_late	FALSE	19.8
110	torpor_late	FALSE	23.8
112	torpor_late	FALSE	24.0
97	torpor_late	FALSE	24.1
114	torpor_late	FALSE	24.7

Leave-one-out impact on within-group CoV (%)

1.5.3 Coefficient of Variation

The Coefficient of Variation (CoV) is a quality metric for the reproducibility of replicate measurements, here visualized using box- and violin-plots.

The same data was used as detailed in the "retention time" section above, with only peptides quantified in at least 3 replicates used for CoV computation in a group. Only samples that are NOT marked 'exclude' in the provided sample metadata and are in a sample group among at least 3 replicates are used for these figures.

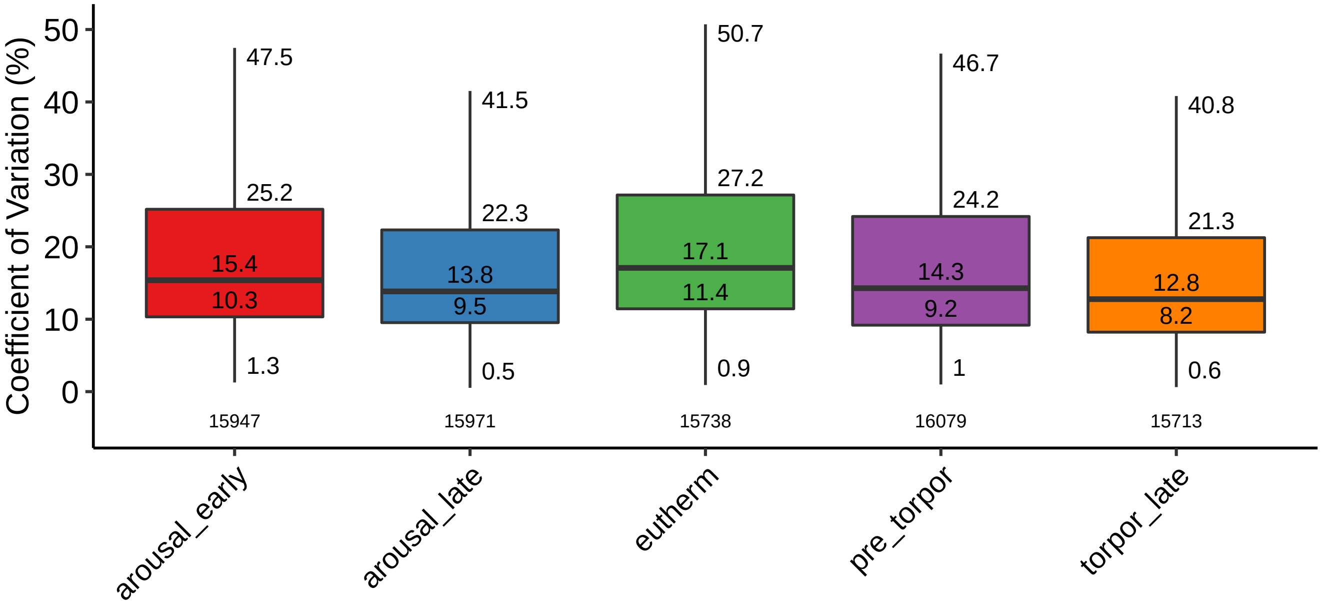

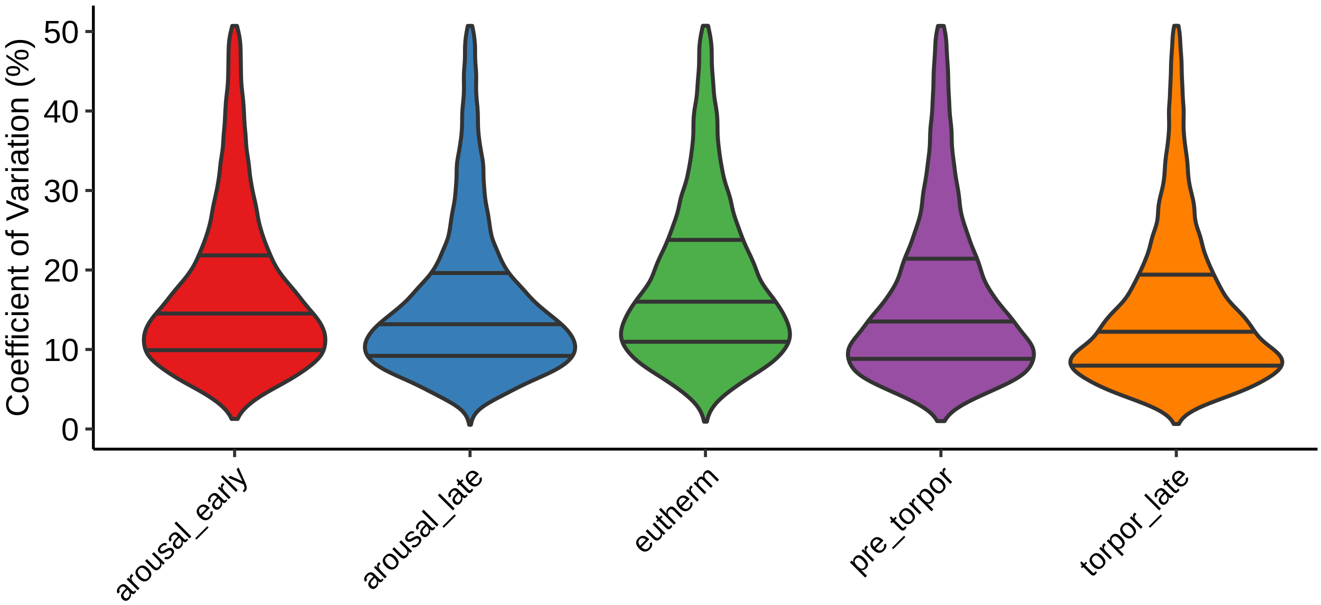

1.6 PCA

A visualization of the first three PCA dimensions illustrates sample clustering. The goal of these figures is to detect global effects from a quality control perspective, such as samples from the same experiment batch clustering together, not to be sensitive to a minor subset of differentially abundant proteins (for which specialized statistical models can be applied downstream).

If additional sample metadata was provided, such as experiment batch, sample-prep dates, gel, etc., multiple PCA figures will be generated with respective color-codings. Users are encouraged to provide relevant experiment information as sample metadata and use these figures to search for unexpected batch effects.

The pcaMethods R package is used here to perform the Probabilistic PCA (PPCA). The set of peptides used for this analysis consists of those peptides that pass your filter criteria in every sample group. If any samples are marked as 'exclude' in the provided sample metadata, an additional PCA plot is generated with these samples included (depicting the 'exclude' samples as square symbols).

Rationale behind data filter
As mentioned above, the aim of the PCA figures is to identify global effects. To achieve this, we compute sample distances on the subset of peptides identified in each group which prevents rarely detected peptides/proteins from having a disproportionate effect on sample clustering. This pertains not only to 'randomly detected contaminant proteins' but also to proteins with abundance levels near the detection limit, which may be detected in only a subset of samples (eg; some measurements will be more successful/sensitive than others).

Figure legends
The first 3 principle components compared visually (1 *vs* 2, 1 *vs* 3, 2 *vs* 3) on the rows. Left- and right-side panels on each row represent the same figure without and with sample labels. The principle components are shown on the axis labels together with their respective percentage of variance explained. Samples marked as 'exclude' in the provided sample metadata, if any, are visualized as square shapes.

PCA of all samples, including those flagged as 'exclude', using 14512 peptides

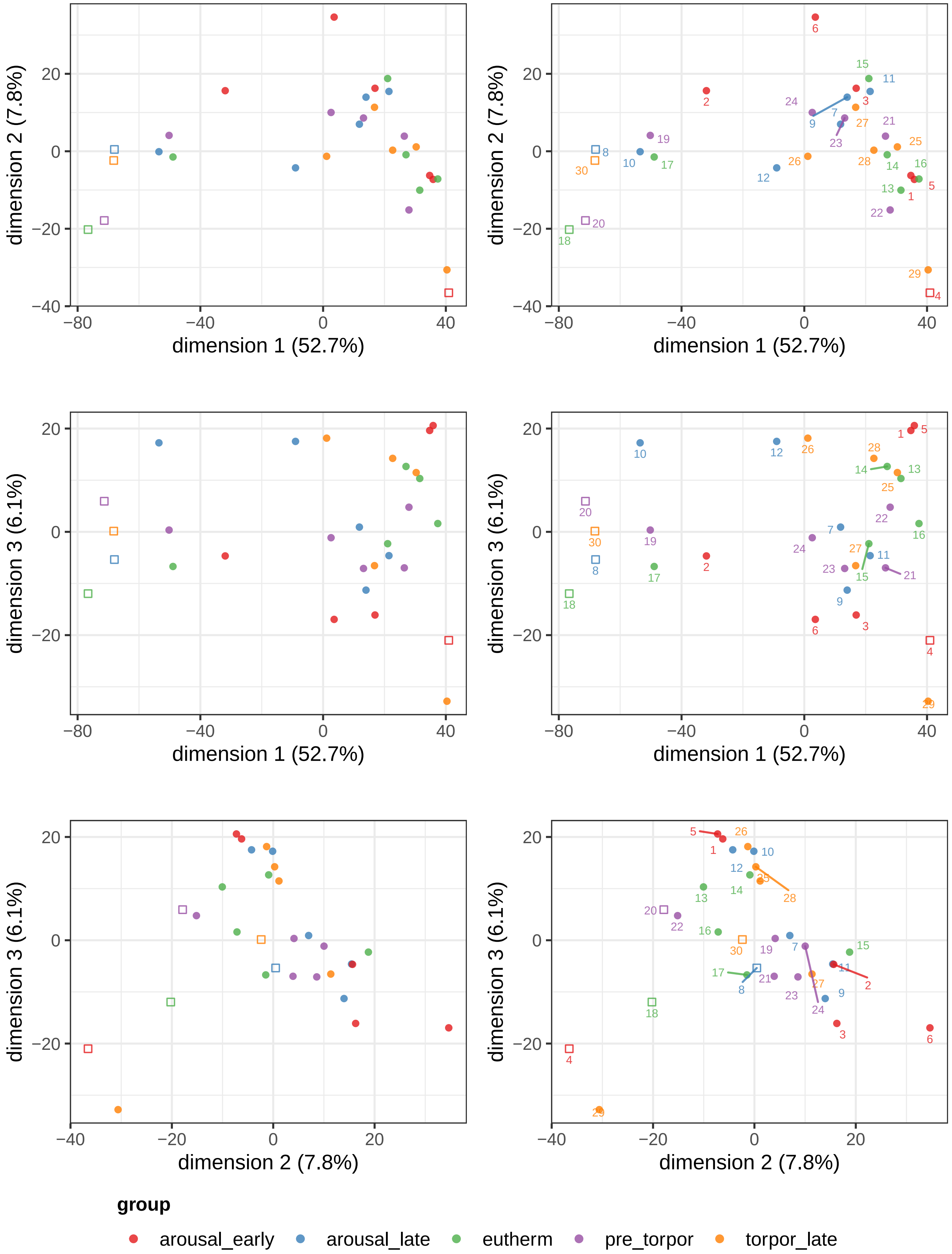

PCA only on samples not flagged as 'exclude', using 13810 peptides

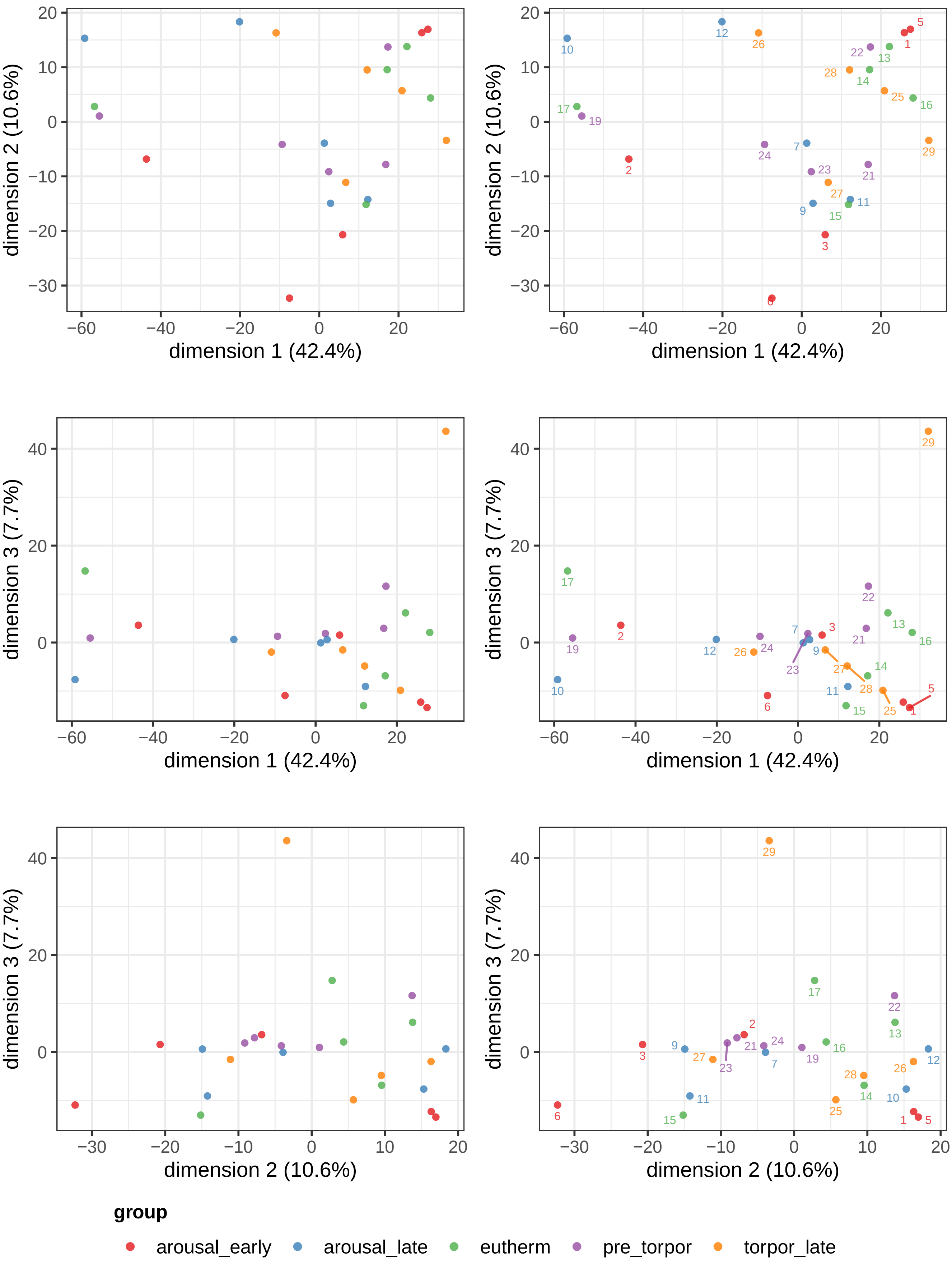

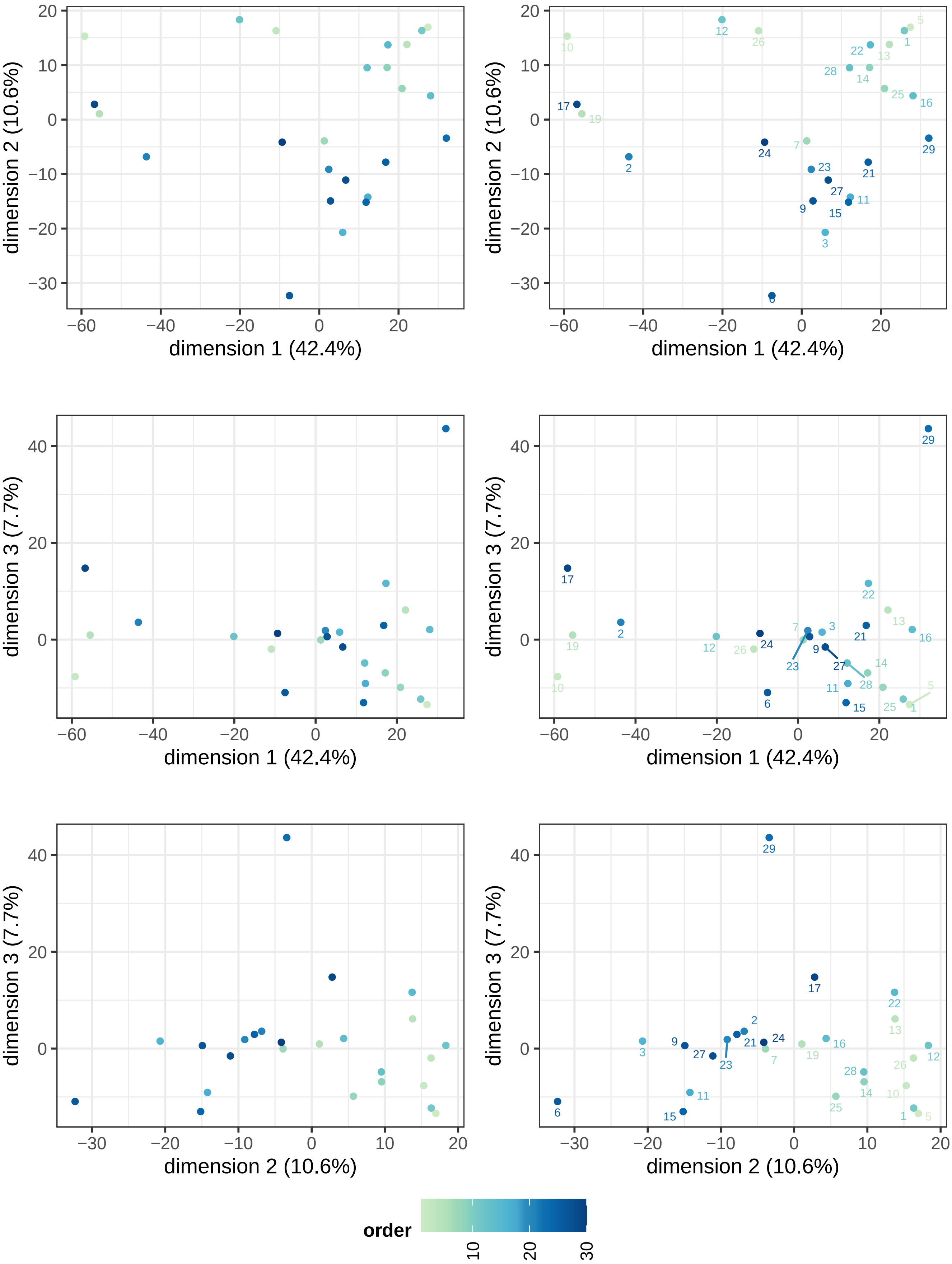
dimension 1 (42.4%)
dimension 2 (10.6%)
dimension 3 (7.7%)
order
10
20
30

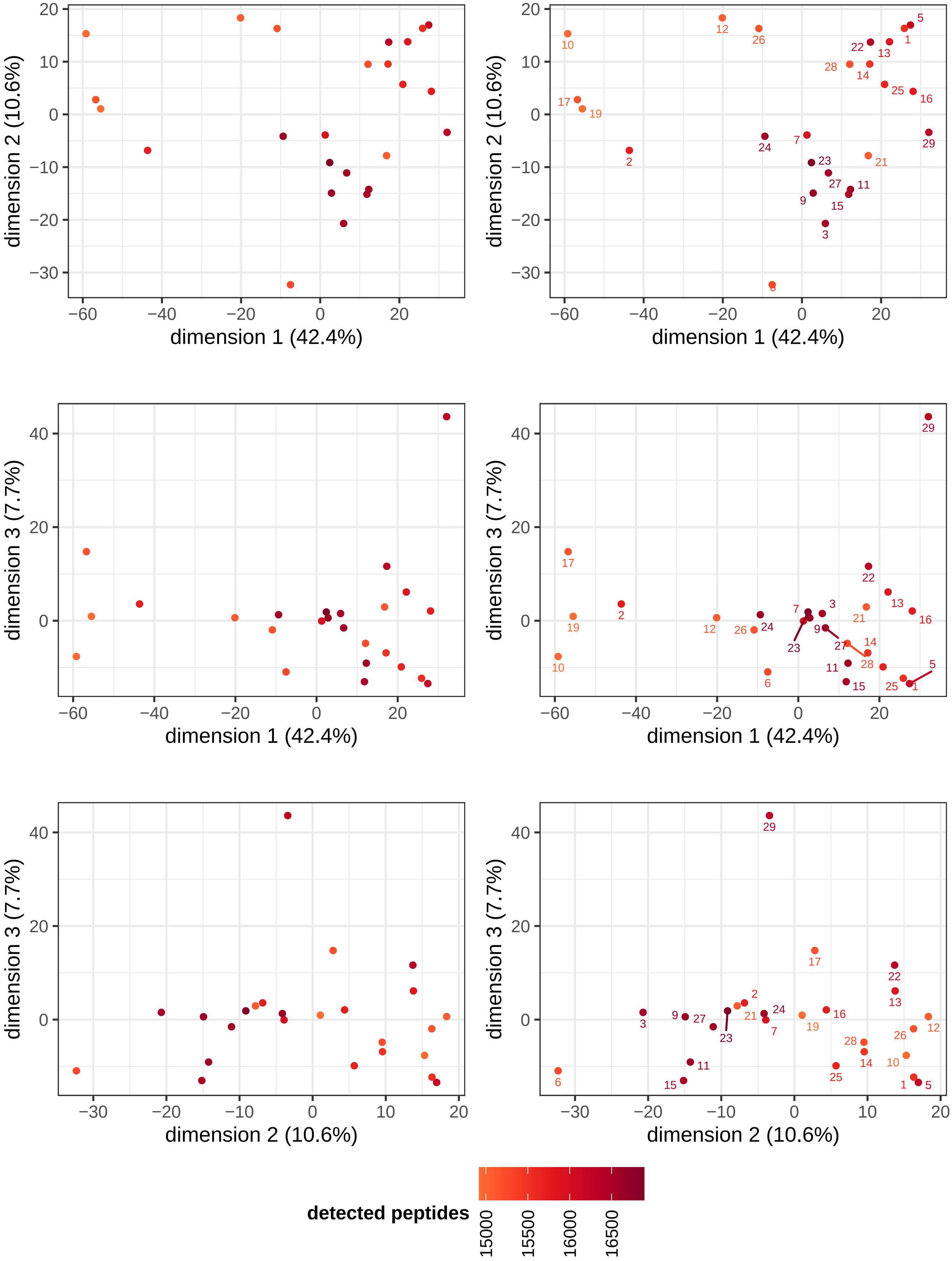
dimension 1 (42.4%)
dimension 2 (10.6%)
dimension 3 (7.7%)
detected peptides
15000
15500
16000
16500

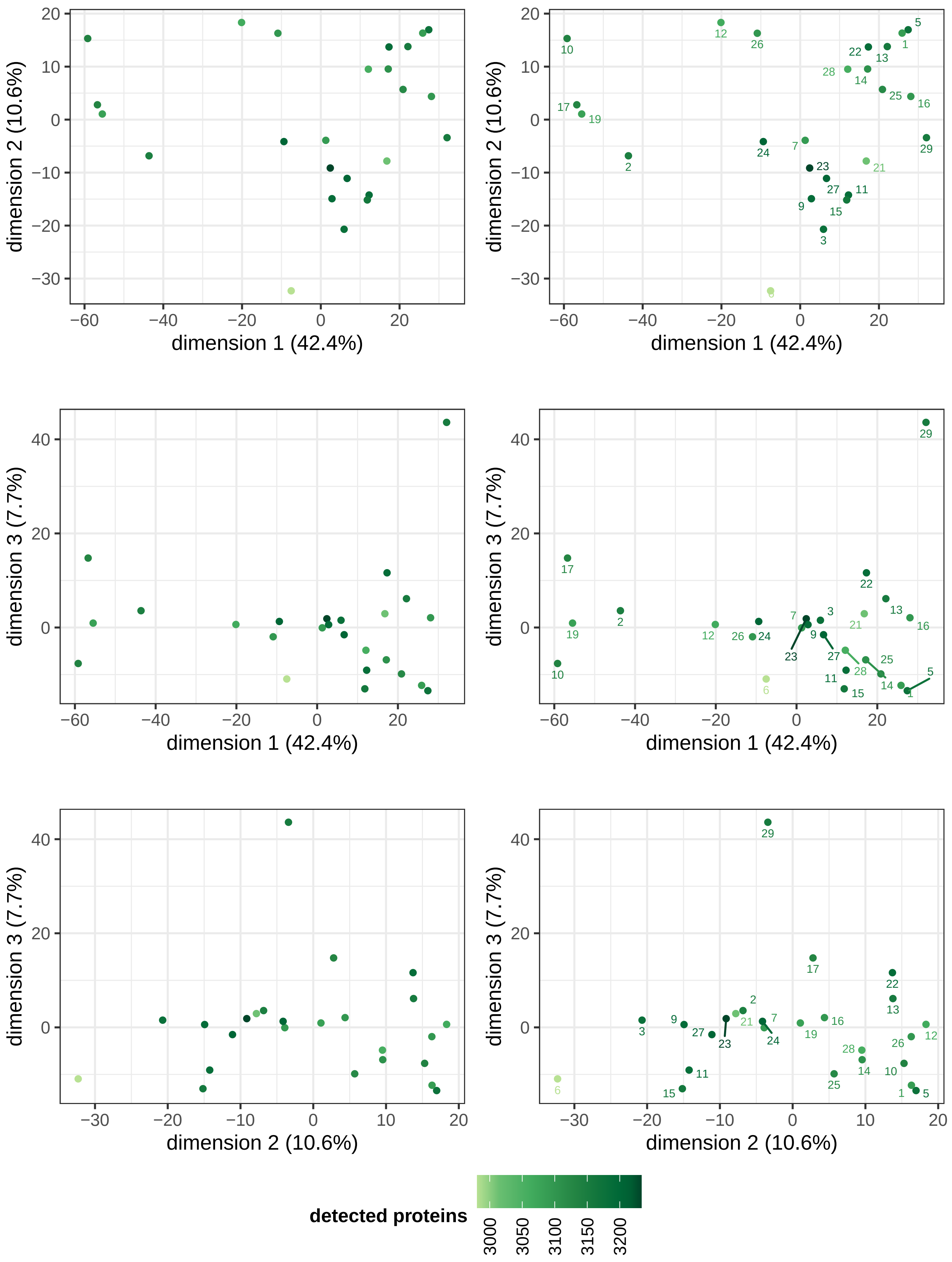
dimension 1 (42.4%)
dimension 2 (10.6%)
dimension 3 (7.7%)
detected proteins
3000
3050
3100
3150
3200

2 Differential abundance analysis

goal: maximize reliable features for quantification

In a pairwise analysis of two groups of samples, only peptides with N data-points in both groups are used for quantitative analysis (where N = defined by user settings). For example; if peptide *p* is consistently quantified in sample groups A and B but not in C/D/E, it can be used when comparing group A *versus* group B but should not be used in any other group comparisons. This approach is particularly suited to maximize the number of peptides used for statistical analysis in experimental designs with many sample groups.

A common alternative strategy is a global filtering approach where peptides are selected based on their properties in the overall dataset (eg; present in x% of samples or x% of replicates in all groups) and subsequentially the resulting data matrix is used for all downstream statistical analyses. In the example above where peptide *p* is present in a subset of sample groups, *p* would either be left out (not present in majority of samples in entire dataset) or erronously used when applying t-statistics to groups B and C (since *p* is not present in group C, it may differentially detected but there are no features available for quantitative analysis)

2.1 torpor_late vs arousal_late

- **user setting:** using 'filter by contrast' peptide filtering approach
- 22607 peptides in 3764 proteins remain in the current contrast after peptide filters and are used for the statistical analysis in this section
- qvalue threshold: 0.05
- log2 foldchange threshold: 0

2.1.1 volcano

The plot title shows the statistical model and contrast (sample groups in the comparison). Left- and right-side figure panels on each row represent the same figure without and with labels for the 25 proteins with lowest p-value.

Bottom figure panels have limited x- and y-axis. For datasets with a small number of strong outliers in p-value or fold-change, which may have a profound effect on the plot scales, this allows inspection of the remainder of the volcano plot without disproportionate influence by 'extreme' values.

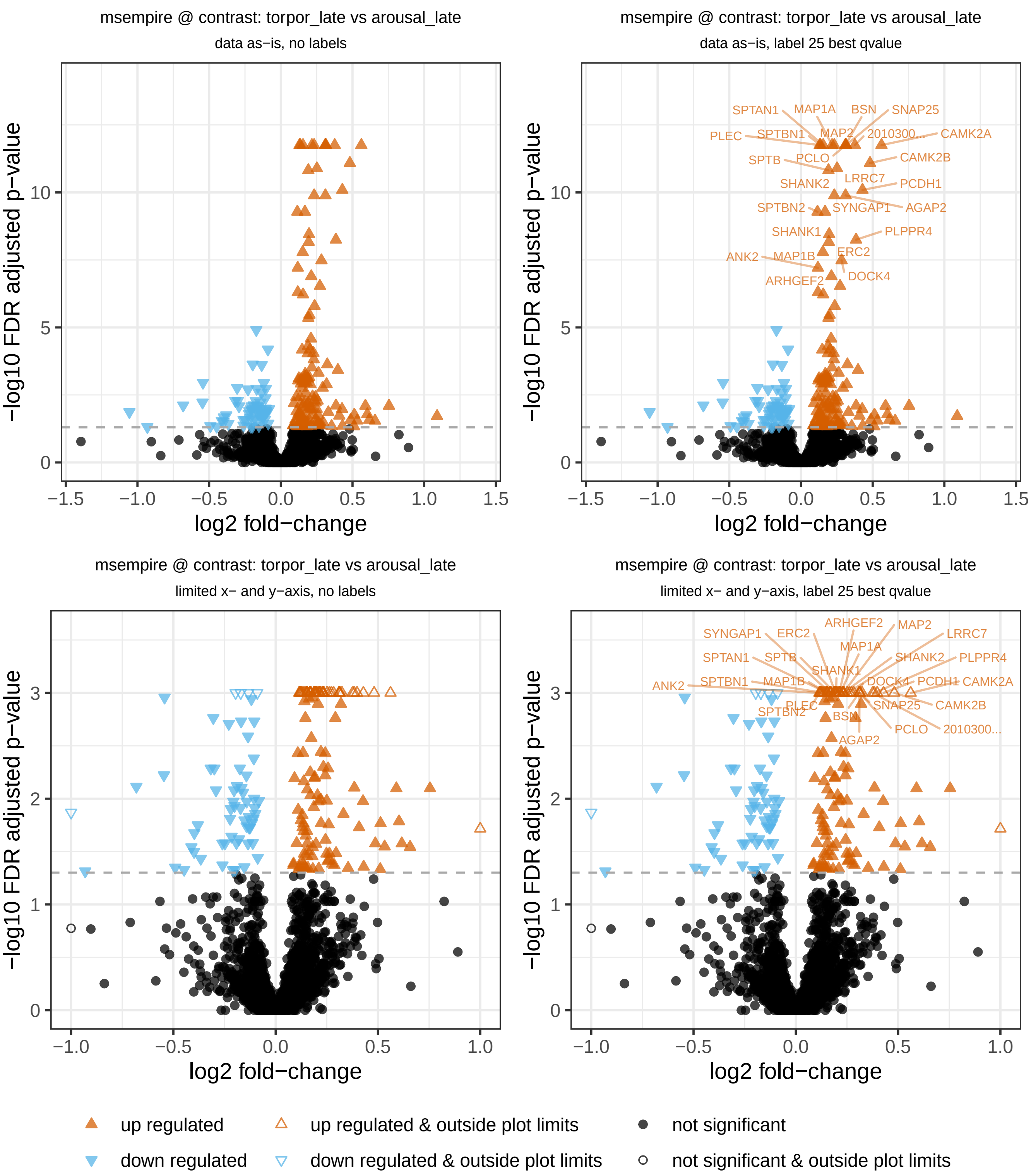
msempire @ contrast: torpor_late vs arousal_late
data as−is, no labels
msempire @ contrast: torpor_late vs arousal_late
data as−is, label 25 best qvalue
SPTAN1
MAP1A
BSN
SNAP25
PLEC
SPTBN1
MAP2
2010300...
CAMK2A
SPTB
PCLO
CAMK2B
SHANK2
LRRC7
PCDH1
SPTBN2
SYNGAP1
AGAP2
SHANK1
PLPPR4
ANK2
MAP1B
ERC2
ARHGEF2
DOCK4
msempire @ contrast: torpor_late vs arousal_late
limited x− and y−axis, no labels
msempire @ contrast: torpor_late vs arousal_late
limited x− and y−axis, label 25 best qvalue
ARHGEF2
MAP2
LRRC7
SYNGAP1
ERC2
MAP1A
SPTAN1
SPTB
SHANK2
PLPPR4
SHANK1
ANK2
SPTBN1
MAP1B
DOCK4
PCDH1
CAMK2A
PLEC
SPTBN2
SNAP25
CAMK2B
BSN
PCLO
2010300...
AGAP2
−log10 FDR adjusted p−value
log2 fold−change
up regulated
down regulated
up regulated & outside plot limits
down regulated & outside plot limits
not significant
not significant & outside plot limits

2.1.2 foldchange distribution

Distributions of estimated foldchanges produced by the statistical models. If the mode is far from 0, consider alternative normalization strategies. Do note the scale on the x-axis, for some experiments the foldchanges are very low which in turn may exaggerate this figure.

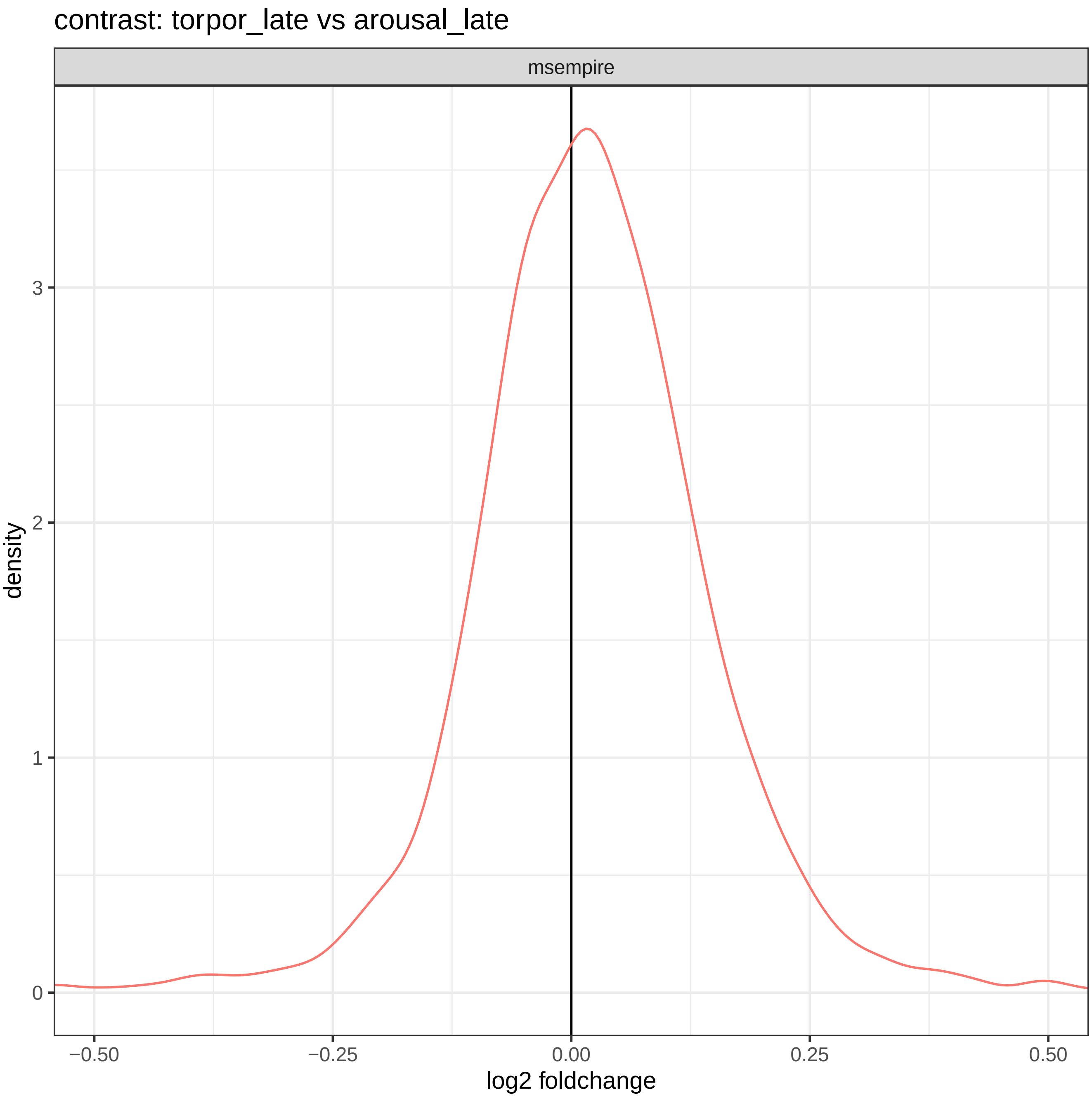

2.1.3 p-value distribution

Histogram of p-values computed by differential expression analysis algorithms, as-is, for quality-control inspection. The horizontal line indicates the expected counts assuming a uniform distribution (total number of p-values divided by number of histogram bins)

See further: https://www.ncbi.nlm.nih.gov/pmc/articles/PMC6164648/

See further: http://varianceexplained.org/statistics/interpreting-pvalue-histogram/

note; the MSqRob and MS-EmpiRe models often yield p-value distributions that show a large peak at p-value 1, these are typically proteins with estimated log foldchanges at/near zero where these models are very sure the null hypothesis cannot be rejected

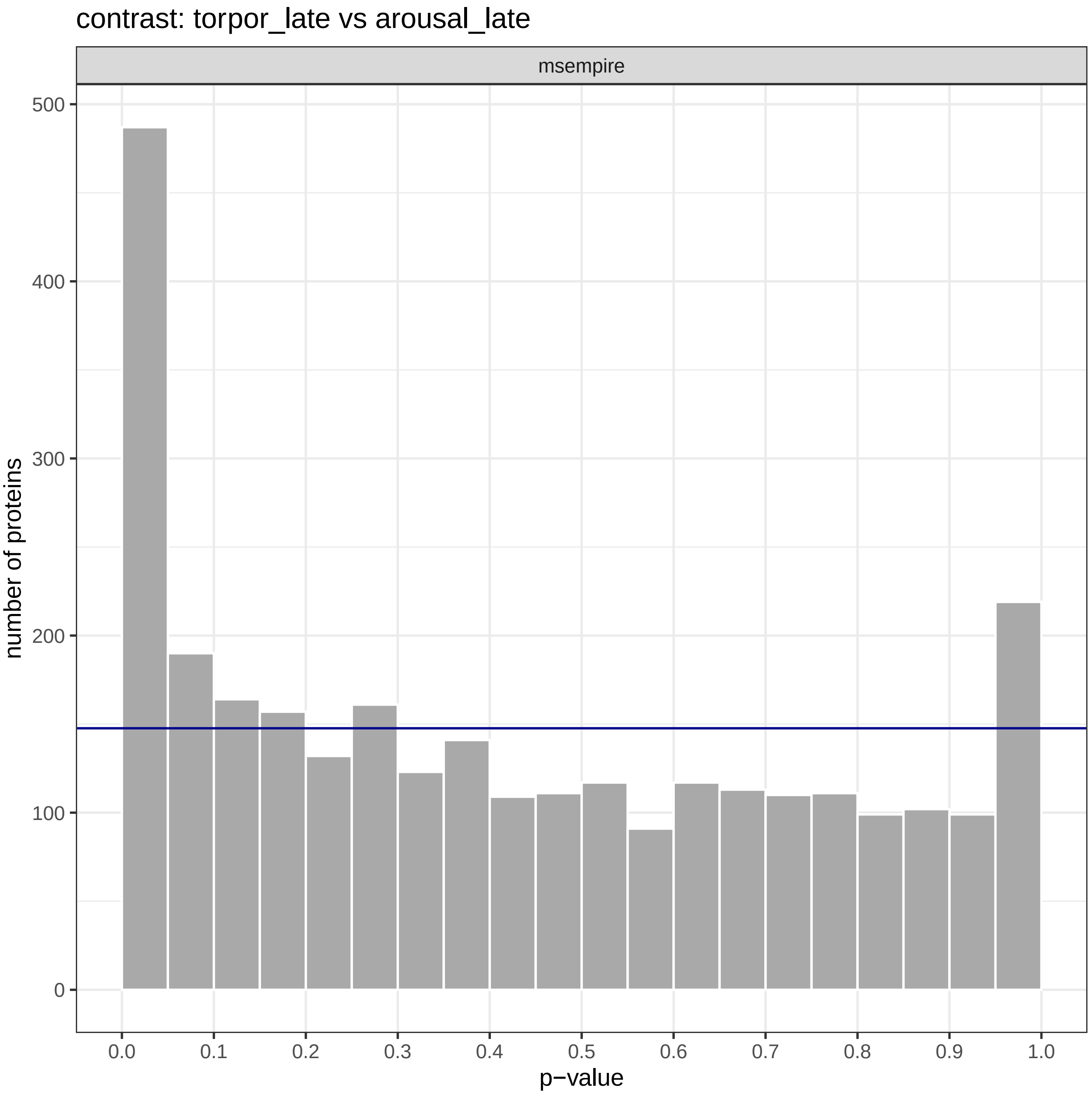

2.1.4 differential detect

Some proteins may not have peptides with sufficient abundance values for differential expression analysis, but do show strong differences in the number of detected peptides between sample groups. A simple z-score is computed for each protein based on the number of detected peptides per sample group. DEA is much more powerful for differential testing, but theze z-scores can offer complementary information in some experimental designs (e.g. a wildtype-knockout APMS study).

Below figure shows the distribution of these scores with thresholds at 2 std. Both the z-scores and the counts these are based upon are available in the statistical result Excel table.

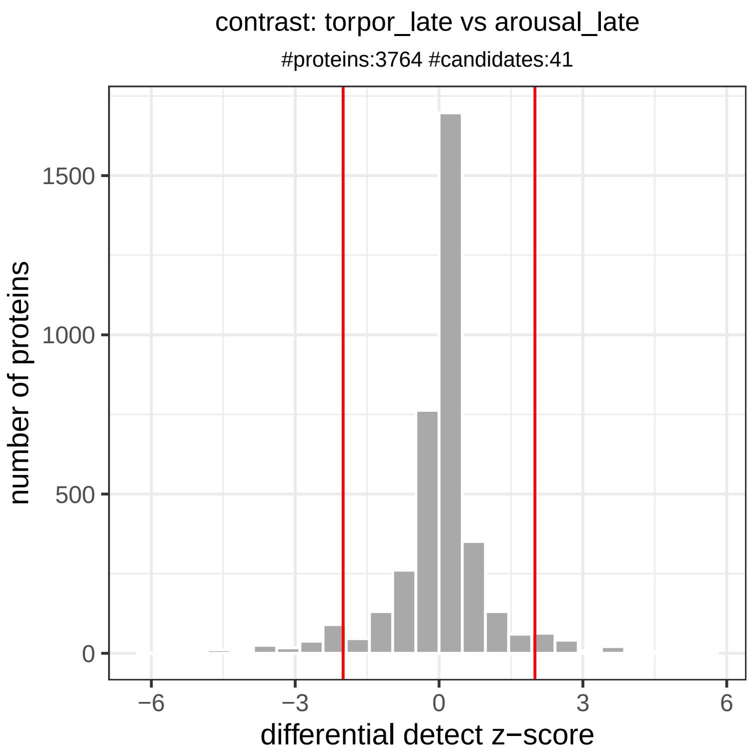

2.2 torpor_late vs eutherm

- **user setting:** using 'filter by contrast' peptide filtering approach
- 22607 peptides in 3764 proteins remain in the current contrast after peptide filters and are used for the statistical analysis in this section
- qvalue threshold: 0.05
- log2 foldchange threshold: 0

2.2.1 volcano

The plot title shows the statistical model and contrast (sample groups in the comparison). Left- and right-side figure panels on each row represent the same figure without and with labels for the 25 proteins with lowest p-value.

Bottom figure panels have limited x- and y-axis. For datasets with a small number of strong outliers in p-value or fold-change, which may have a profound effect on the plot scales, this allows inspection of the remainder of the volcano plot without disproportionate influence by 'extreme' values.

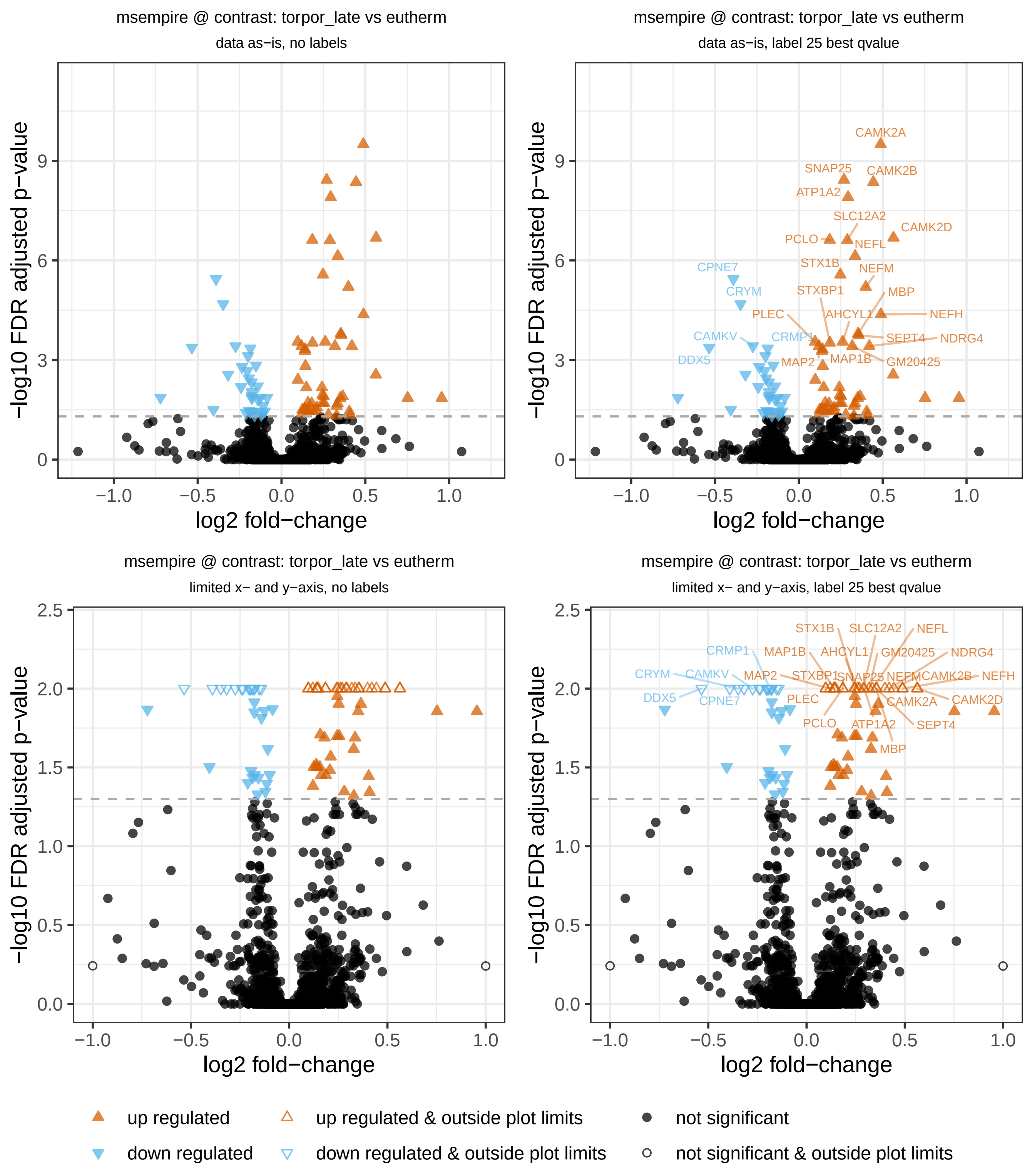

msempire @ contrast: torpor_late vs eutherm
data as−is, no labels
−log10 FDR adjusted p−value
log2 fold−change
msempire @ contrast: torpor_late vs eutherm
data as−is, label 25 best qvalue
CAMK2A
SNAP25
CAMK2B
ATP1A2
SLC12A2
CAMK2D
PCLO
NEFL
CPNE7
STX1B
NEFM
CRYM
STXBP1
MBP
PLEC
AHCYL1
NEFH
CAMKV
CRMP1
SEPT4
NDRG4
DDX5
MAP2
MAP1B
GM20425
msempire @ contrast: torpor_late vs eutherm
limited x− and y−axis, no labels
msempire @ contrast: torpor_late vs eutherm
limited x− and y−axis, label 25 best qvalue
up regulated
up regulated & outside plot limits
not significant
down regulated
down regulated & outside plot limits
not significant & outside plot limits

2.2.2 foldchange distribution

Distributions of estimated foldchanges produced by the statistical models. If the mode is far from 0, consider alternative normalization strategies. Do note the scale on the x-axis, for some experiments the foldchanges are very low which in turn may exaggerate this figure.

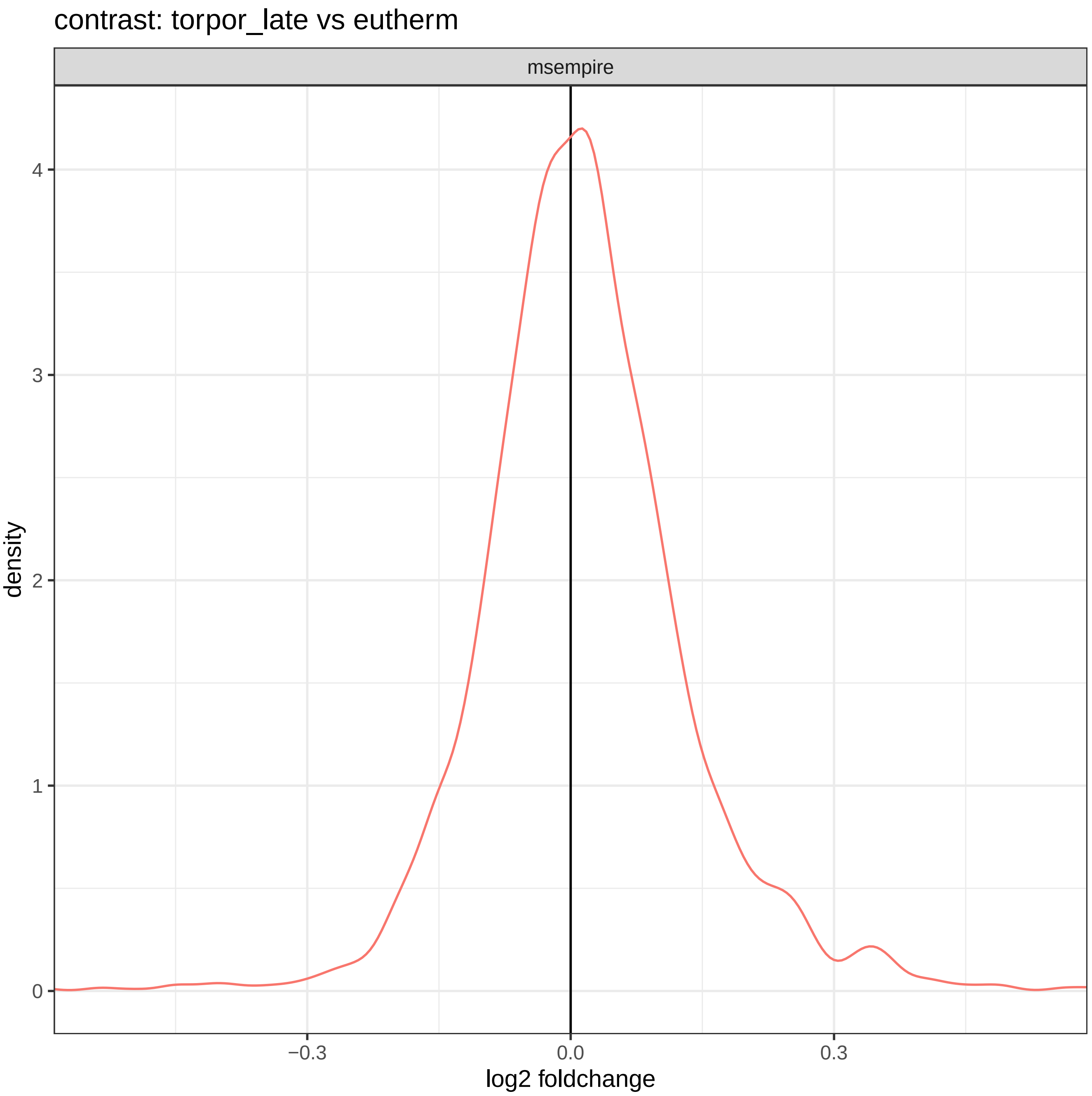

2.2.3 p-value distribution

Histogram of p-values computed by differential expression analysis algorithms, as-is, for quality-control inspection. The horizontal line indicates the expected counts assuming a uniform distribution (total number of p-values divided by number of histogram bins)

See further: https://www.ncbi.nlm.nih.gov/pmc/articles/PMC6164648/

See further: http://varianceexplained.org/statistics/interpreting-pvalue-histogram/

note; the MSqRob and MS-EmpiRe models often yield p-value distributions that show a large peak at p-value 1, these are typically proteins with estimated log foldchanges at/near zero where these models are very sure the null hypothesis cannot be rejected

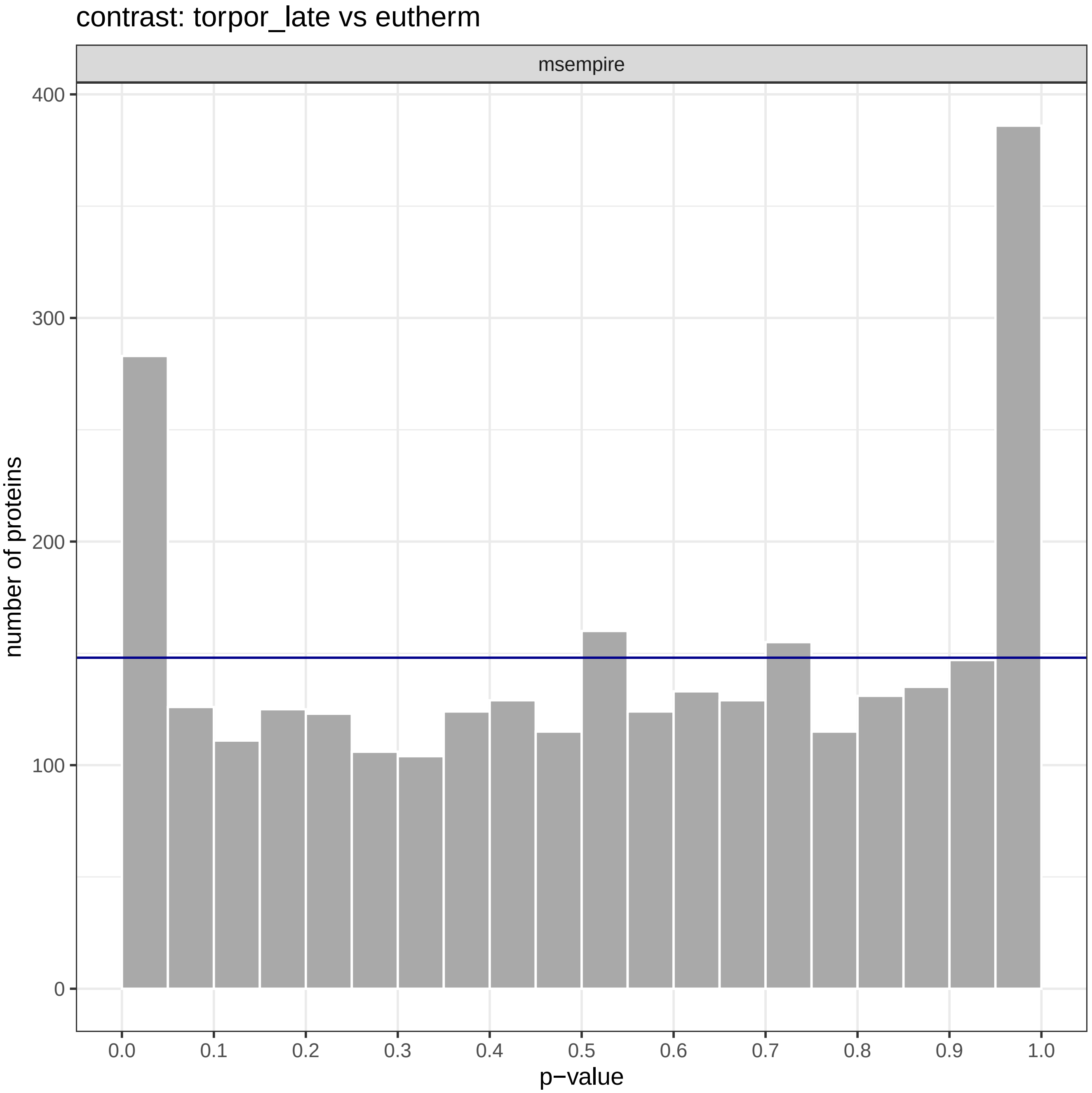

2.2.4 differential detect

Some proteins may not have peptides with sufficient abundance values for differential expression analysis, but do show strong differences in the number of detected peptides between sample groups. A simple z-score is computed for each protein based on the number of detected peptides per sample group. DEA is much more powerful for differential testing, but theze z-scores can offer complementary information in some experimental designs (e.g. a wildtype-knockout APMS study).

Below figure shows the distribution of these scores with thresholds at 2 std. Both the z-scores and the counts these are based upon are available in the statistical result Excel table.

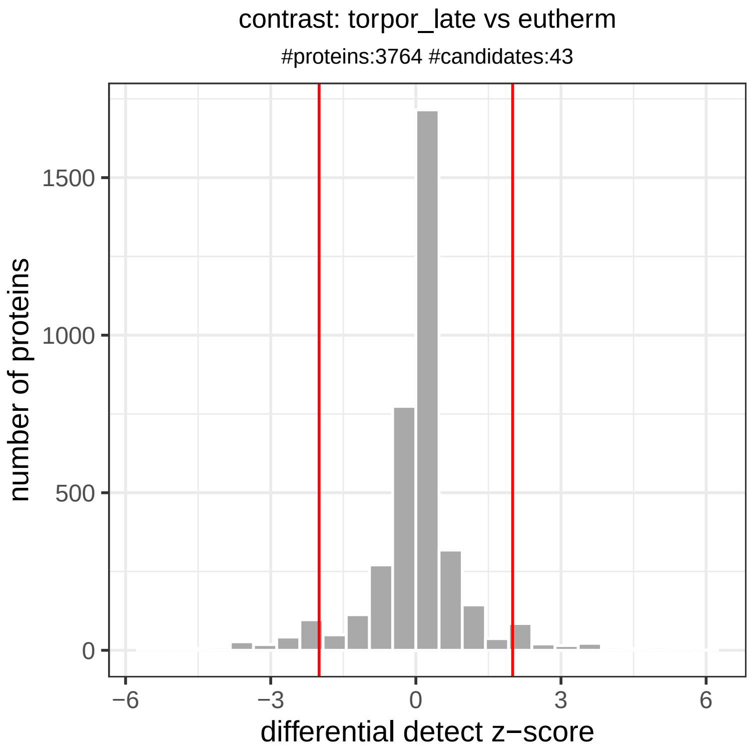

2.3 arousal_late vs eutherm

- **user setting:** using 'filter by contrast' peptide filtering approach
- 22607 peptides in 3764 proteins remain in the current contrast after peptide filters and are used for the statistical analysis in this section
- qvalue threshold: 0.05
- log2 foldchange threshold: 0

2.3.1 volcano

The plot title shows the statistical model and contrast (sample groups in the comparison). Left- and right-side figure panels on each row represent the same figure without and with labels for the 25 proteins with lowest p-value.

Bottom figure panels have limited x- and y-axis. For datasets with a small number of strong outliers in p-value or fold-change, which may have a profound effect on the plot scales, this allows inspection of the remainder of the volcano plot without disproportionate influence by 'extreme' values.

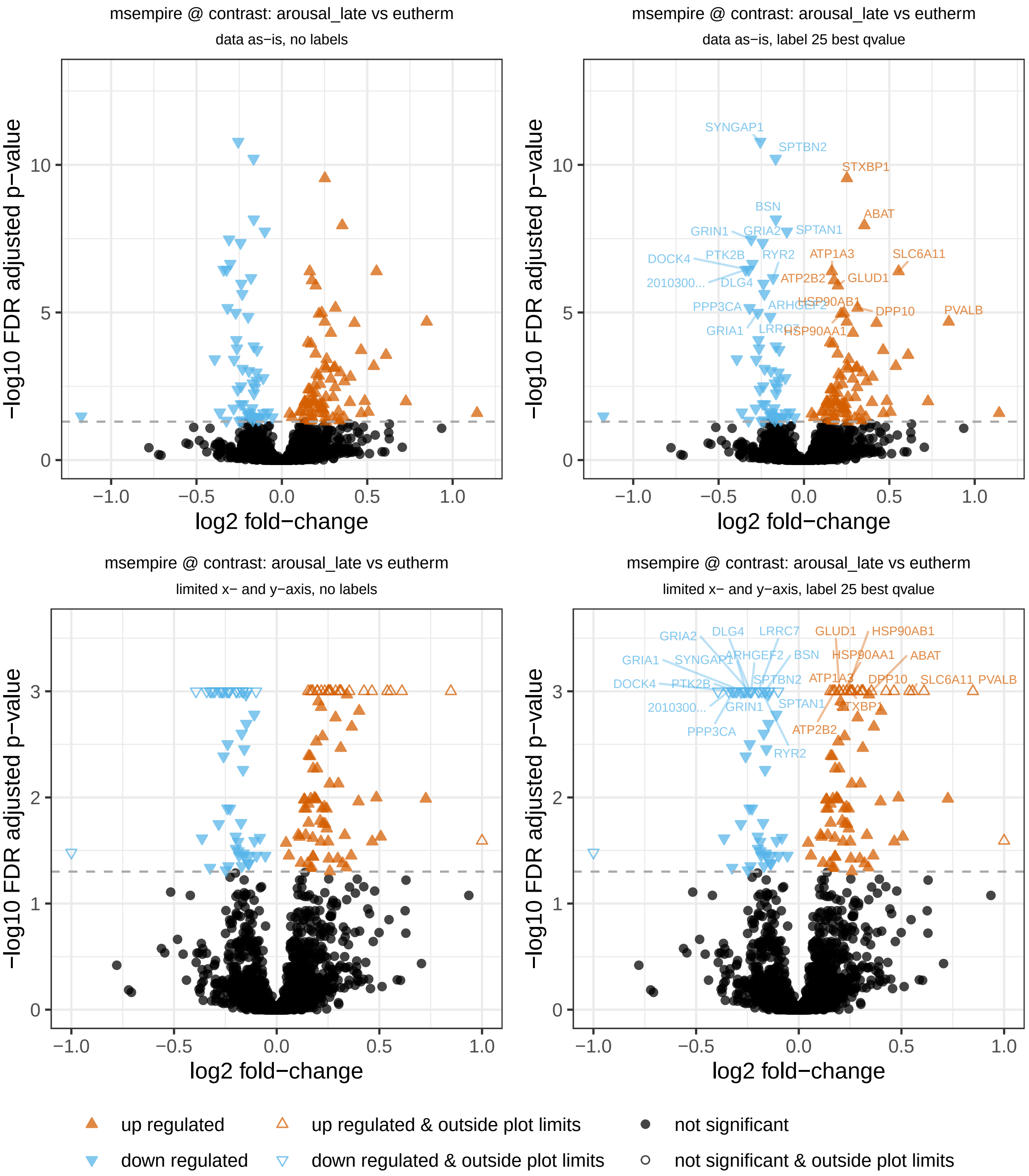
msempire @ contrast: arousal_late vs eutherm
data as−is, no labels
−log10 FDR adjusted p−value
log2 fold−change
msempire @ contrast: arousal_late vs eutherm
data as−is, label 25 best qvalue
SYNGAP1
SPTBN2
STXBP1
BSN
ABAT
GRIN1
GRIA2
SPTAN1
DOCK4
PTK2B
RYR2
ATP1A3
SLC6A11
2010300...
DLG4
ATP2B2
GLUD1
PPP3CA
ARHGEF2
HSP90AB1
DPP10
PVALB
GRIA1
LRRC7
HSP90AA1
msempire @ contrast: arousal_late vs eutherm
limited x− and y−axis, no labels
msempire @ contrast: arousal_late vs eutherm
limited x− and y−axis, label 25 best qvalue
up regulated
up regulated & outside plot limits
not significant
down regulated
down regulated & outside plot limits
not significant & outside plot limits

2.3.2 foldchange distribution

Distributions of estimated foldchanges produced by the statistical models. If the mode is far from 0, consider alternative normalization strategies. Do note the scale on the x-axis, for some experiments the foldchanges are very low which in turn may exaggerate this figure.

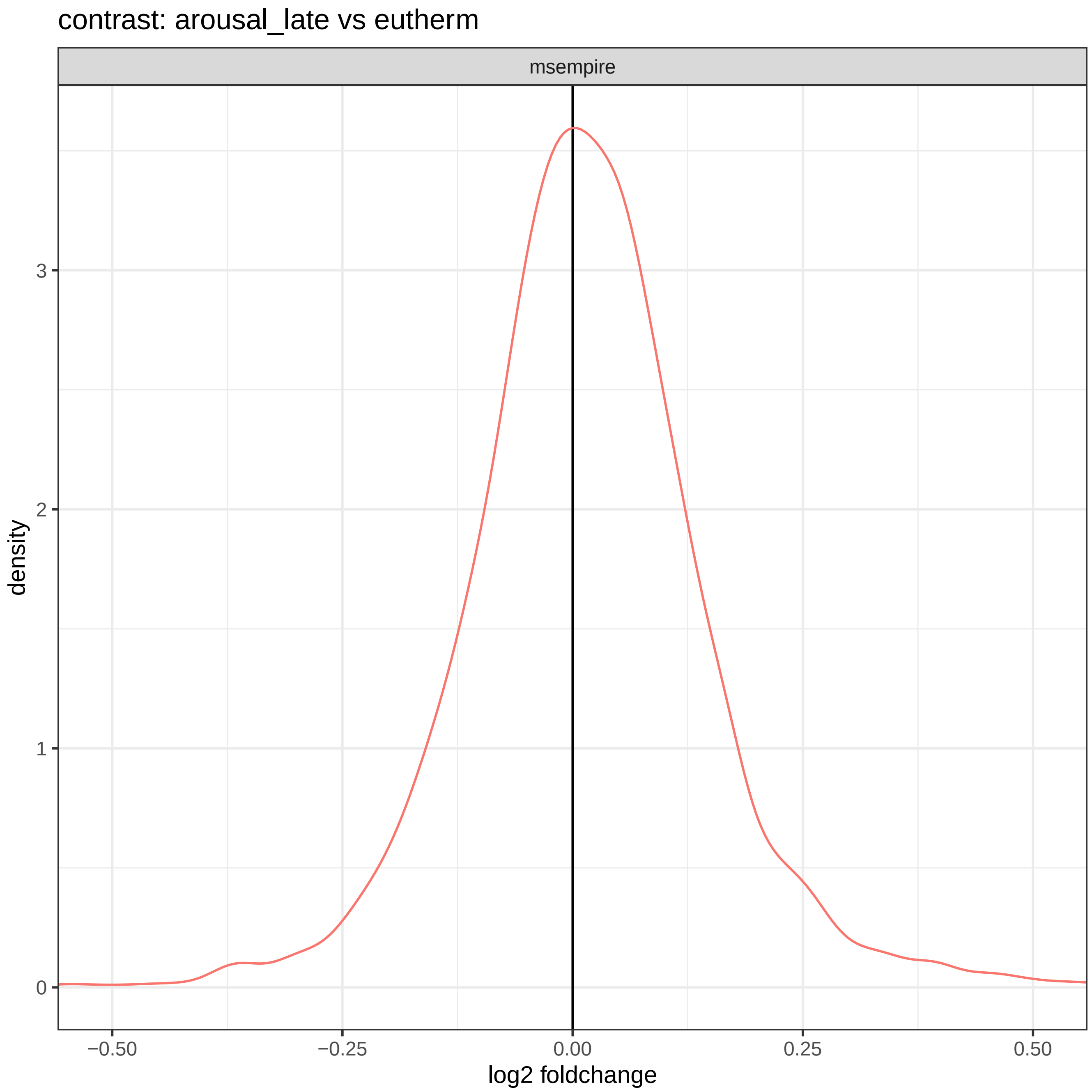

2.3.3 p-value distribution

Histogram of p-values computed by differential expression analysis algorithms, as-is, for quality-control inspection. The horizontal line indicates the expected counts assuming a uniform distribution (total number of p-values divided by number of histogram bins)

See further: https://www.ncbi.nlm.nih.gov/pmc/articles/PMC6164648/

See further: http://varianceexplained.org/statistics/interpreting-pvalue-histogram/

note; the MSqRob and MS-EmpiRe models often yield p-value distributions that show a large peak at p-value 1, these are typically proteins with estimated log foldchanges at/near zero where these models are very sure the null hypothesis cannot be rejected

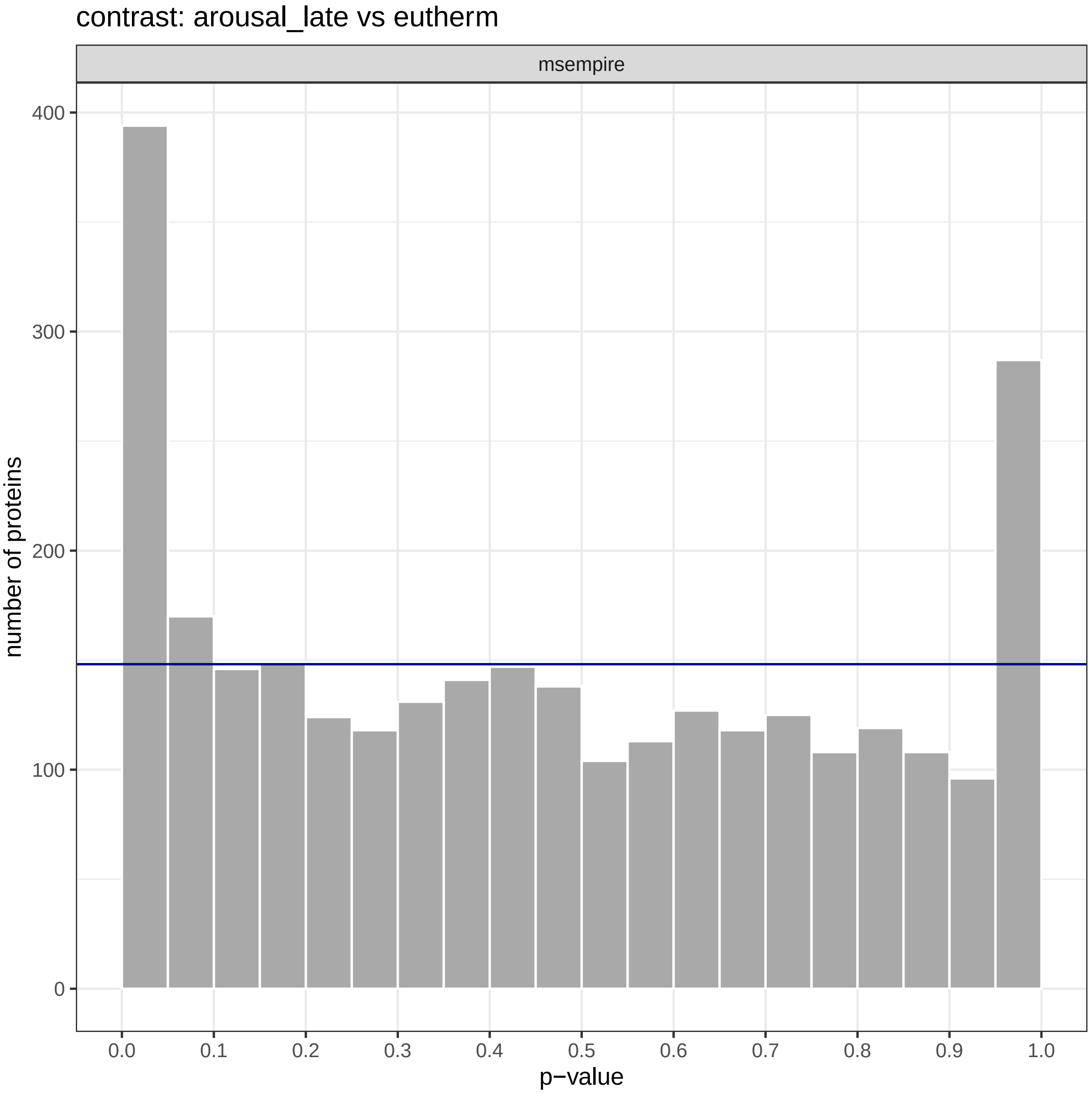

2.3.4 differential detect

Some proteins may not have peptides with sufficient abundance values for differential expression analysis, but do show strong differences in the number of detected peptides between sample groups. A simple z-score is computed for each protein based on the number of detected peptides per sample group. DEA is much more powerful for differential testing, but theze z-scores can offer complementary information in some experimental designs (e.g. a wildtype-knockout APMS study).

Below figure shows the distribution of these scores with thresholds at 2 std. Both the z-scores and the counts these are based upon are available in the statistical result Excel table.

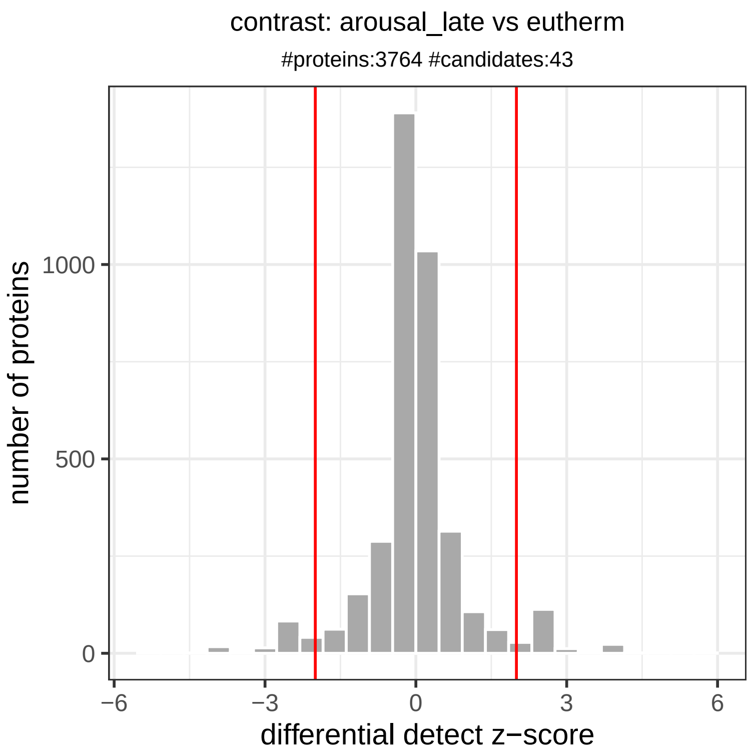

3 Summary of differential testing

Differential Expression Analysis: number of proteins found statistically significant.

contrast	algorithm	#test	#hits	top10 significant
arousal_late vs eutherm	msempire	2963	138	syngap1, sptbn2, stxbp1, bsn, abat, sptan1, grin1, gria2, ptk2b, 2010300...
torpor_late vs arousal_late	msempire	2953	194	map1a, sptan1, bsn, camk2a, map2, snap25, sptbn1, plec, pclo, 2010300...
torpor_late vs eutherm	msempire	2961	83	camk2a, snap25, camk2b, atp1a2, camk2d, pclo, slc12a2, nefl, stx1b, cpne7

Differential Detection: prioritize proteins with more peptide detections in some group. A simple metric to complement results from DEA, which is the main result (eg; consider proteins with too few data points for DEA).

contrast	#test	#hits	top10 candidates
arousal_late vs eutherm	3764	43	bmpr2, rcn1, lchn, arl6ip1, hectd4, apool, abcb10, sbds, sirt3, htra1
torpor_late vs arousal_late	3764	41	mdga1, mlycd, mrpl24, c1qa, abcb5, pycr3, golgb1, ccdc93, prkg2, gpaa1
torpor_late vs eutherm	3764	43	gpaa1, mdga1, lrrc10b, mfge8, enpp4, cntnap4, znf706, matr3, rragc, epb41l1

4 log

[info] reading Spectronaut report...

[info] 22639/24043 precursors remain after selecting the 'best' precursor for each modified sequence

[info] Spectronaut report contains malformed protein IDs (pipe symbol replaced by whitespace), fixing now...

[info] 22 / 21293 unique target (plain)sequences were not in the provided spectral library and thus removed

[info] 9522/9523 protein accessions and 3764/3765 protein groups were mapped to provided fasta file(s)

[progress] filtered proteins: tr|IRT|IRT_KITBiognosysiRTKitFusion

[info] 1/3765 proteins matching your filters were removed from the dataset

[info] contrast: torpor_late vs arousal_late

[info] contrast: torpor_late vs eutherm

[info] contrast: arousal_late vs eutherm

[progress] caching filter data took 5 seconds

[progress] peptide filtering and normalization took 57 seconds

[info] differential abundance analysis for contrast: torpor_late vs arousal_late

[info] using data from peptide filter: filter by contrast

[progress] MS-EmpiRe took 1.9 minutes

[info] differential abundance analysis for contrast: torpor_late vs eutherm

[info] using data from peptide filter: filter by contrast

[progress] MS-EmpiRe took 1.7 minutes

[info] differential abundance analysis for contrast: arousal_late vs eutherm

[info] using data from peptide filter: filter by contrast

[progress] MS-EmpiRe took 1.7 minutes

[progress] creating PDF report...

[progress] report: constructing plots specific for each contrast

[progress] report: rendering report (this may take a while depending on dataset size)

[progress] RT plots: preparing data took 17 seconds

[progress] RT plots: creating plots took 3 seconds

[progress] leave-one-out CoV plot computations took 11 seconds

5 R command history

This shows the history commands from your R script that starts this pipeline, thereby automatically documenting the parameters/settings used. All lines of executed code since (last) importing data using this R package are shown.

Using this feature

Do not use RStudio's `source` option to execute our pipeline since it will only write `source(...yourscript.R)` to the session history, and consequentially that is all you see in this 'code log'. Instead, select all lines in your script (`control + A`) and then "run" the selected code (either click the run button in RStudio, or use `control + enter`). All lines shown in this section are the same as shown in the RStudio 'History' pane (a tab on the top-right of its UI).

```
dataset = import_dataset_spectronaut(
  filename = "C:/DATA/Christina_torpor/spectronaut/20200106_132945_20190321_20170216_Christina_HC_P2_Full mouse proteome 201804_Report.t
  confidence_threshold = 0.01,
  use_normalized_intensities = FALSE,
  do_plot = F
)
dataset = update_protein_mapping_from_maxquant(dataset,
  path_maxquant = "C:/DATA/DIA_spectral_libraries/remco_speclib_20190301_HC_P2_Full_mouse_proteome_201804/maxquant",
  remove_shared = TRUE
)
dataset = import_fasta(dataset,
  files = c(
    "C:/DATA/DIA_spectral_libraries/remco_speclib_20190301_HC_P2_Full_mouse_proteome_201804/fasta/UP000000589_10090.fasta",
    "C:/DATA/DIA_spectral_libraries/remco_speclib_20190301_HC_P2_Full_mouse_proteome_201804/fasta/UP000000589_10090_additional.fasta"
  )
)
dataset = remove_proteins_by_name(dataset,
  remove_irt_peptides = TRUE
)
dataset = import_sample_metadata(dataset,
  filename = "C:/DATA/Christina_torpor/sample-metadata_outliers.xlsx"
)
dataset = setup_contrasts(dataset,
  contrast_list = list(
    c(
      "torpor_late",
      "arousal_late"
    ),
    c(
      "torpor_late",
      "eutherm"
    ),
    c(
      "arousal_late",
      "eutherm"
    )
  )
)
dataset = analysis_quickstart(dataset,
  filter_min_detect = 3,
  filter_topn_peptides = 0,
  filter_by_contrast = TRUE,
  norm_algorithm = c(
    "vsn",
    "modebetween_protein"
  ),
  dea_algorithm = "msempire",
  dea_qvalue_threshold = 0.05,
  output_qc_report = TRUE,
  output_dir = "C:/DATA/Christina_torpor/data_analysis_results",
  output_within_timestamped_subdirectory = TRUE
)
```

6 R session info

The computer system and versioning of all R packages used to run this analysis are shown below to facilitate, in combination with the previous section, reproducibility.

setting	value
version	R version 3.6.3 (2020-02-29)
os	Windows 10 x64
system	x86_64, mingw32
ui	RStudio
language	(EN)
collate	English_United States.1252
ctype	English_United States.1252
tz	Europe/Berlin
date	2020-07-10

System

package	loadedversion	source
dplyr	1.0.0	CRAN (R 3.6.3)
ggplot2	3.3.2	CRAN (R 3.6.3)
msdap	0.2.5	Github (ftwkoopmans/msdap@b77d02f)
rlang	0.4.6	CRAN (R 3.6.3)
testthat	2.3.2	CRAN (R 3.6.3)
tibble	3.0.1	CRAN (R 3.6.3)
tidyr	1.1.0	CRAN (R 3.6.3)

Attached packages

package	loadedversion	source
abind	1.4-5	CRAN (R 3.6.0)
affy	1.64.0	Bioconductor
affyio	1.56.0	Bioconductor
arrangements	1.1.8	CRAN (R 3.6.3)
askpass	1.1	CRAN (R 3.6.3)
assertthat	0.2.1	CRAN (R 3.6.3)
backports	1.1.7	CRAN (R 3.6.3)
Biobase	2.46.0	Bioconductor
BiocGenerics	0.32.0	Bioconductor
BiocManager	1.30.10	CRAN (R 3.6.3)
BiocParallel	1.20.1	Bioconductor
bit	1.1-15.2	CRAN (R 3.6.2)
bit64	0.9-7	CRAN (R 3.6.2)
bitops	1.0-6	CRAN (R 3.6.0)
blob	1.2.1	CRAN (R 3.6.3)
boot	1.3-24	CRAN (R 3.6.3)
broom	0.5.6	CRAN (R 3.6.3)
callr	3.4.3	CRAN (R 3.6.3)
car	3.0-8	CRAN (R 3.6.3)
carData	3.0-4	CRAN (R 3.6.3)
caTools	1.18.0	CRAN (R 3.6.3)
cellranger	1.1.0	CRAN (R 3.6.3)
cli	2.0.2	CRAN (R 3.6.3)
codetools	0.2-16	CRAN (R 3.6.3)
colorspace	1.4-1	CRAN (R 3.6.3)
cowplot	1.0.0	CRAN (R 3.6.3)
crayon	1.3.4	CRAN (R 3.6.3)
curl	4.3	CRAN (R 3.6.3)
data.table	1.12.8	CRAN (R 3.6.3)
DBI	1.1.0	CRAN (R 3.6.3)
desc	1.2.0	CRAN (R 3.6.3)
devtools	2.3.0	CRAN (R 3.6.3)
digest	0.6.25	CRAN (R 3.6.3)
doParallel	1.0.15	CRAN (R 3.6.3)
ellipsis	0.3.1	CRAN (R 3.6.3)
evaluate	0.14	CRAN (R 3.6.3)
fansi	0.4.1	CRAN (R 3.6.3)
farver	2.0.3	CRAN (R 3.6.3)
fastmap	1.0.1	CRAN (R 3.6.3)
forcats	0.5.0	CRAN (R 3.6.3)
foreach	1.5.0	CRAN (R 3.6.3)
foreign	0.8-75	CRAN (R 3.6.3)
formatR	1.7	CRAN (R 3.6.3)
fs	1.4.1	CRAN (R 3.6.3)
gdata	2.18.0	CRAN (R 3.6.3)
generics	0.0.2	CRAN (R 3.6.3)
ggpubr	0.4.0	CRAN (R 3.6.3)
ggrepel	0.8.2	CRAN (R 3.6.3)
ggsignif	0.6.0	CRAN (R 3.6.3)
glue	1.4.1	CRAN (R 3.6.3)

package	loadedversion	source
gmp	0.6-0	CRAN (R 3.6.3)
gplots	3.0.4	CRAN (R 3.6.3)
gridExtra	2.3	CRAN (R 3.6.3)
gtable	0.3.0	CRAN (R 3.6.3)
gtools	3.8.2	CRAN (R 3.6.3)
haven	2.3.1	CRAN (R 3.6.3)
hms	0.5.3	CRAN (R 3.6.3)
htmltools	0.5.0	CRAN (R 3.6.3)
htmlwidgets	1.5.1	CRAN (R 3.6.3)
httpuv	1.5.4	CRAN (R 3.6.3)
impute	1.60.0	Bioconductor
IRanges	2.20.2	Bioconductor
iterators	1.0.12	CRAN (R 3.6.3)
jsonlite	1.7.0	CRAN (R 3.6.3)
KernSmooth	2.23-16	CRAN (R 3.6.3)
knitr	1.29	CRAN (R 3.6.3)
labeling	0.3	CRAN (R 3.6.0)
later	1.1.0.1	CRAN (R 3.6.3)
lattice	0.20-38	CRAN (R 3.6.3)
learnr	0.10.1	CRAN (R 3.6.3)
lifecycle	0.2.0	CRAN (R 3.6.3)
limma	3.42.2	Bioconductor
lme4	1.1-23	CRAN (R 3.6.3)
magrittr	1.5	CRAN (R 3.6.3)
MALDIquant	1.19.3	CRAN (R 3.6.3)
markdown	1.1	CRAN (R 3.6.3)
MASS	7.3-51.5	CRAN (R 3.6.3)
Matrix	1.2-18	CRAN (R 3.6.3)
matrixStats	0.56.0	CRAN (R 3.6.3)
memoise	1.1.0	CRAN (R 3.6.3)
mime	0.9	CRAN (R 3.6.2)
minqa	1.2.4	CRAN (R 3.6.3)
msEmpiRe	0.1.0	Github (zimmerlab/MS-EmpiRe@8a85757)
MSnbase	2.12.0	Bioconductor
munsell	0.5.0	CRAN (R 3.6.3)
mzID	1.24.0	Bioconductor
mzR	2.20.0	Bioconductor
ncdf4	1.17	CRAN (R 3.6.1)
nlme	3.1-144	CRAN (R 3.6.3)
nloptr	1.2.2.1	CRAN (R 3.6.3)
openxlsx	4.1.5	CRAN (R 3.6.3)
patchwork	1.0.1	CRAN (R 3.6.3)
pcaMethods	1.78.0	Bioconductor
pdftools	2.3.1	CRAN (R 3.6.3)
pillar	1.4.4	CRAN (R 3.6.3)
pkgbuild	1.0.8	CRAN (R 3.6.3)
pkgconfig	2.0.3	CRAN (R 3.6.3)
pkgload	1.1.0	CRAN (R 3.6.3)
plyr	1.8.6	CRAN (R 3.6.3)
preprocessCore	1.48.0	Bioconductor

package	loadedversion	source
prettyunits	1.1.1	CRAN (R 3.6.3)
pROC	1.16.2	CRAN (R 3.6.3)
processx	3.4.2	CRAN (R 3.6.3)
promises	1.1.1	CRAN (R 3.6.3)
ProtGenerics	1.18.0	Bioconductor
ps	1.3.3	CRAN (R 3.6.3)
purrr	0.3.4	CRAN (R 3.6.3)
qpdf	1.1	CRAN (R 3.6.3)
R6	2.4.1	CRAN (R 3.6.3)
RColorBrewer	1.1-2	CRAN (R 3.6.0)
Rcpp	1.0.4.6	CRAN (R 3.6.3)
readr	1.3.1	CRAN (R 3.6.3)
readxl	1.3.1	CRAN (R 3.6.3)
remotes	2.1.1	CRAN (R 3.6.3)
rio	0.5.16	CRAN (R 3.6.3)
rmarkdown	2.3	CRAN (R 3.6.3)
rprojroot	1.3-2	CRAN (R 3.6.3)
RSQLite	2.2.0	CRAN (R 3.6.3)
rstatix	0.6.0	CRAN (R 3.6.3)
rstudioapi	0.11	CRAN (R 3.6.3)
S4Vectors	0.24.4	Bioconductor
scales	1.1.1	CRAN (R 3.6.3)
sessioninfo	1.1.1	CRAN (R 3.6.3)
shiny	1.5.0	CRAN (R 3.6.3)
shinyFiles	0.8.0	CRAN (R 3.6.3)
shinyjs	1.1	CRAN (R 3.6.3)
sortable	0.4.2	CRAN (R 3.6.3)
statmod	1.4.34	CRAN (R 3.6.3)
stringi	1.4.6	CRAN (R 3.6.2)
stringr	1.4.0	CRAN (R 3.6.3)
styler	1.3.2	CRAN (R 3.6.3)
tidyselect	1.1.0	CRAN (R 3.6.3)
tinytex	0.24	CRAN (R 3.6.3)
usethis	1.6.1	CRAN (R 3.6.3)
utf8	1.1.4	CRAN (R 3.6.3)
vctrs	0.3.1	CRAN (R 3.6.3)
viridis	0.5.1	CRAN (R 3.6.3)
viridisLite	0.3.0	CRAN (R 3.6.3)
vsn	3.54.0	Bioconductor
withr	2.2.0	CRAN (R 3.6.3)
xfun	0.15	CRAN (R 3.6.3)
XML	3.99-0.3	CRAN (R 3.6.3)
xtable	1.8-4	CRAN (R 3.6.3)
yaml	2.2.1	CRAN (R 3.6.3)
zip	2.0.4	CRAN (R 3.6.3)
zlibbioc	1.32.0	Bioconductor

Packages that are not attached
